# Supplementary figures and images for: Evaluation and application of summary statistic imputation to discover new height-associated loci
Source: PLoS Genet. 2018 May 21;14(5):e1007371. doi: 10.1371/journal.pgen.1007371 (PMC5983877; doi:10.1371/journal.pgen.1007371)

Number of SNVs imputed

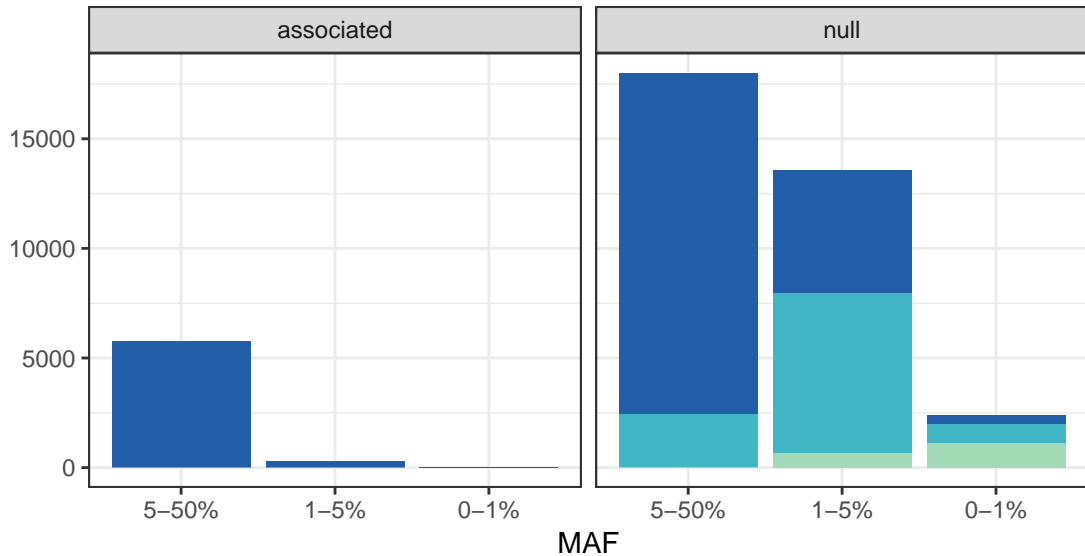

Supplement: S1 Fig — This figure shows how many of the null and associated SNVs were categorised into common, low-frequency and rare MAF subgroups, and into well-imputed, medium imputed and badly imputed imputation subgroups. Associated SNVs are presented in the left window, and null SNVs are presented in the right window. MAF category (x-axis), # of SNVs on the y-axis, colour refers to imputation quality category. (PDF) [file pgen.1007371.s001.pdf]

Fraction of SNVs imputed

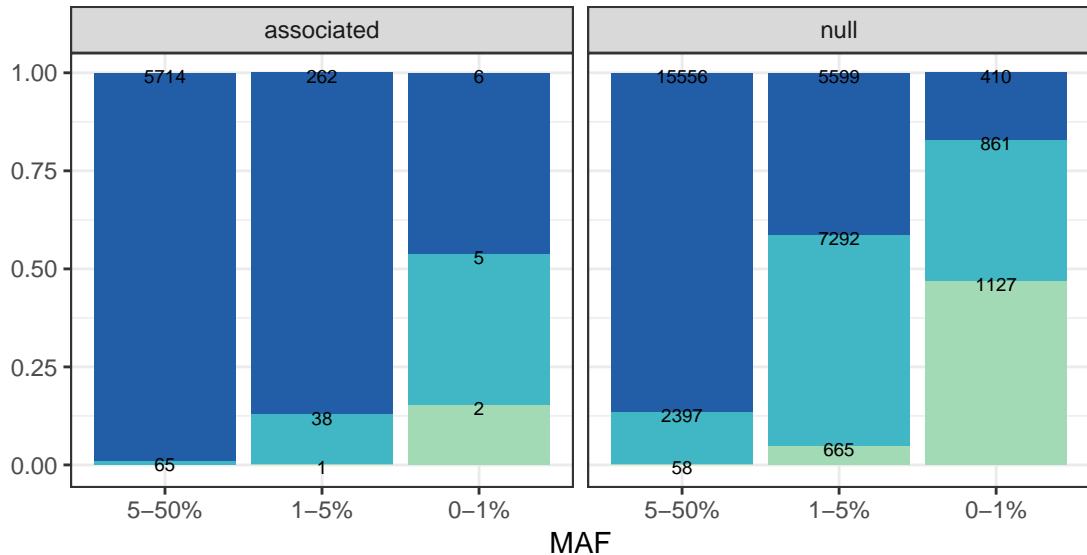

Supplement: S2 Fig — This figure shows the fraction of badly-, medium- and well-imputed SNVs within each MAF subgroup. Null and associated SNVs were categorised into common, low-frequency and rare MAF subgroup, and into well-imputed, medium imputed and badly imputed imputation subgroup. Associated SNVs are presented in the left window, and null SNVs are presented in the right window. MAF category (x-axis), fraction of SNVs on the y-axis, colour refers to imputation quality category. Numbers within the stacked barplot refer to the number of SNVs imputed in each subgroup. (PDF) [file pgen.1007371.s002.pdf]

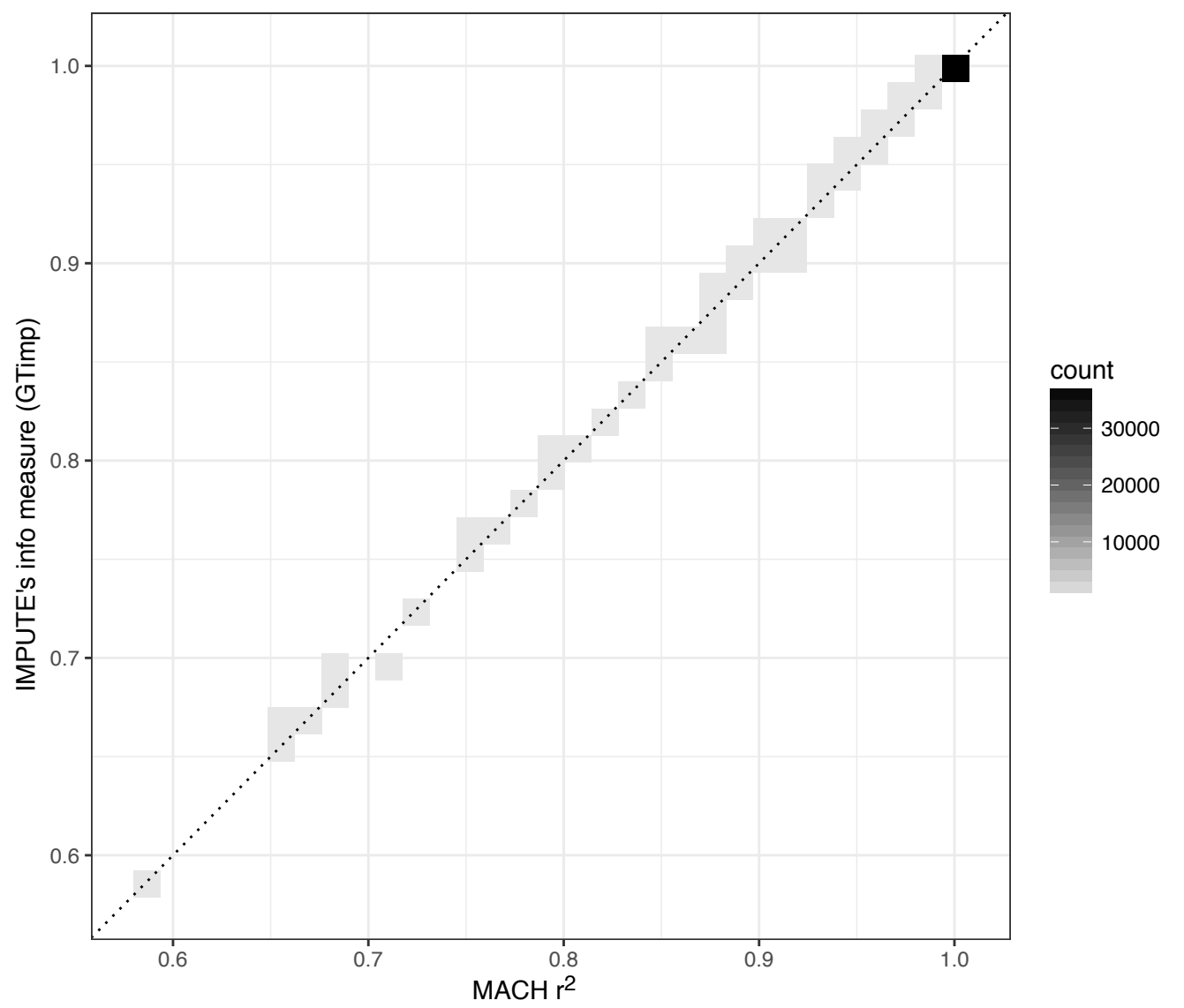

Supplement: S3 Fig — MACH r^2 [46] (x-axis) versus IMPUTE’s info measure used by genotype imputation (y-axis). To avoid clumping of dots, we used tiles varying from grey (few dots) to black (many dots). The identity line is dotted. (PDF) [file pgen.1007371.s003.pdf]

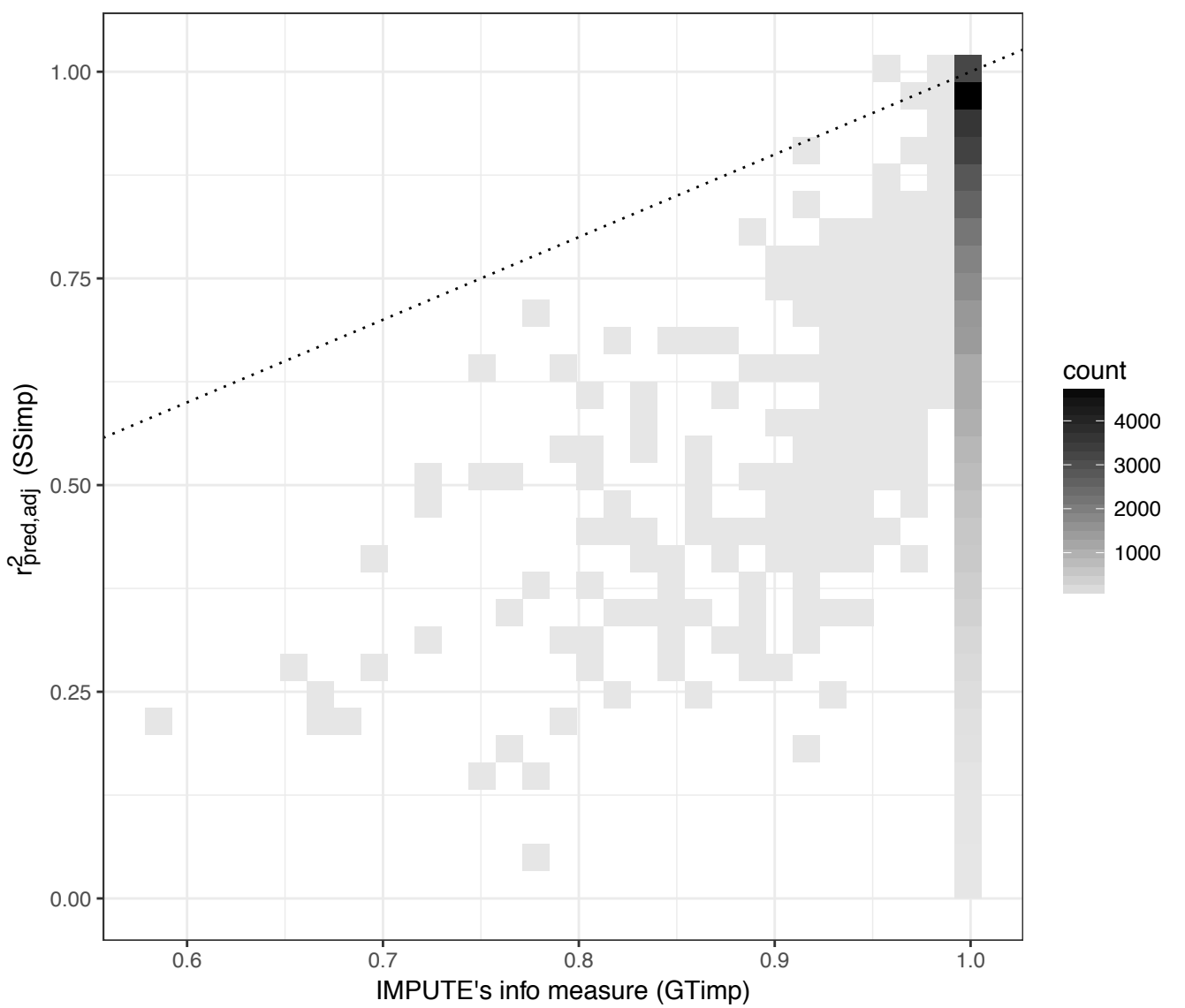

Supplement: S4 Fig — IMPUTE’s info measure used by genotype imputation (x-axis) vs r^pred,adj2 used by summary statistics imputation (y-axis). To avoid clumping of dots, we used tiles varying from grey (few dots) to black (many dots). The identity line is dotted. (PDF) [file pgen.1007371.s004.pdf]

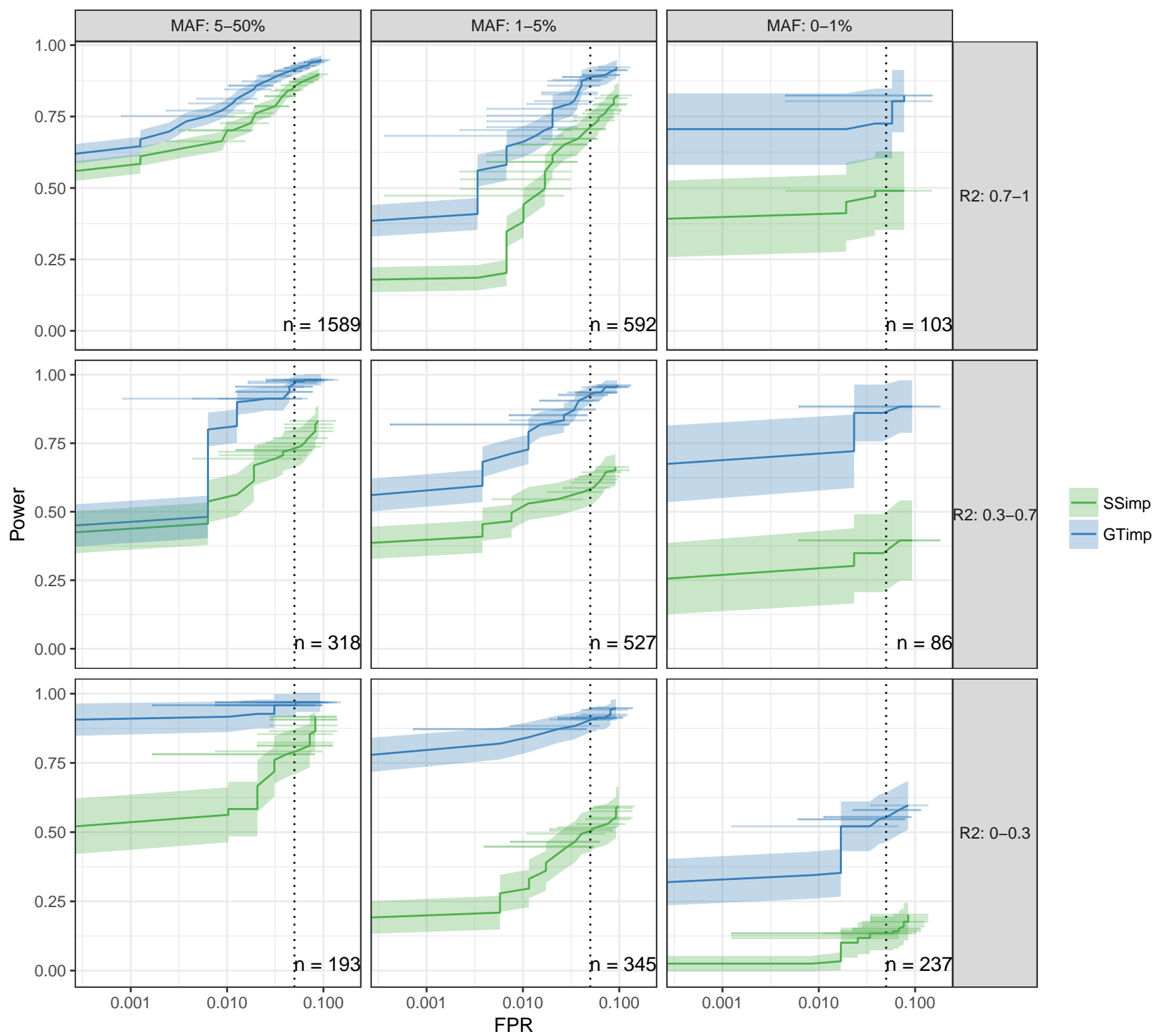

Supplement: S5 Fig — This figure compares false positive rate (FPR) (x-axis on log10-scale) versus power (y-axis) for genotype imputation (blue) and summary statistics imputation (green) for different significance thresholds (α). It includes 95%-confidence intervals in both directions (vertically as a ribbon and horizontally as lines). This figure is a zoom into the bottom-left area of Fig 7 and shows FPR between 0 and 0.1. The coloured dots represent the α = 0.05. The vertical, dashed line represents FPR = 0.05. Results are grouped according to MAF (columns) and imputation quality (rows) categories. (PDF) [file pgen.1007371.s005.pdf]

Only if LD > 0.3 with height associated variant

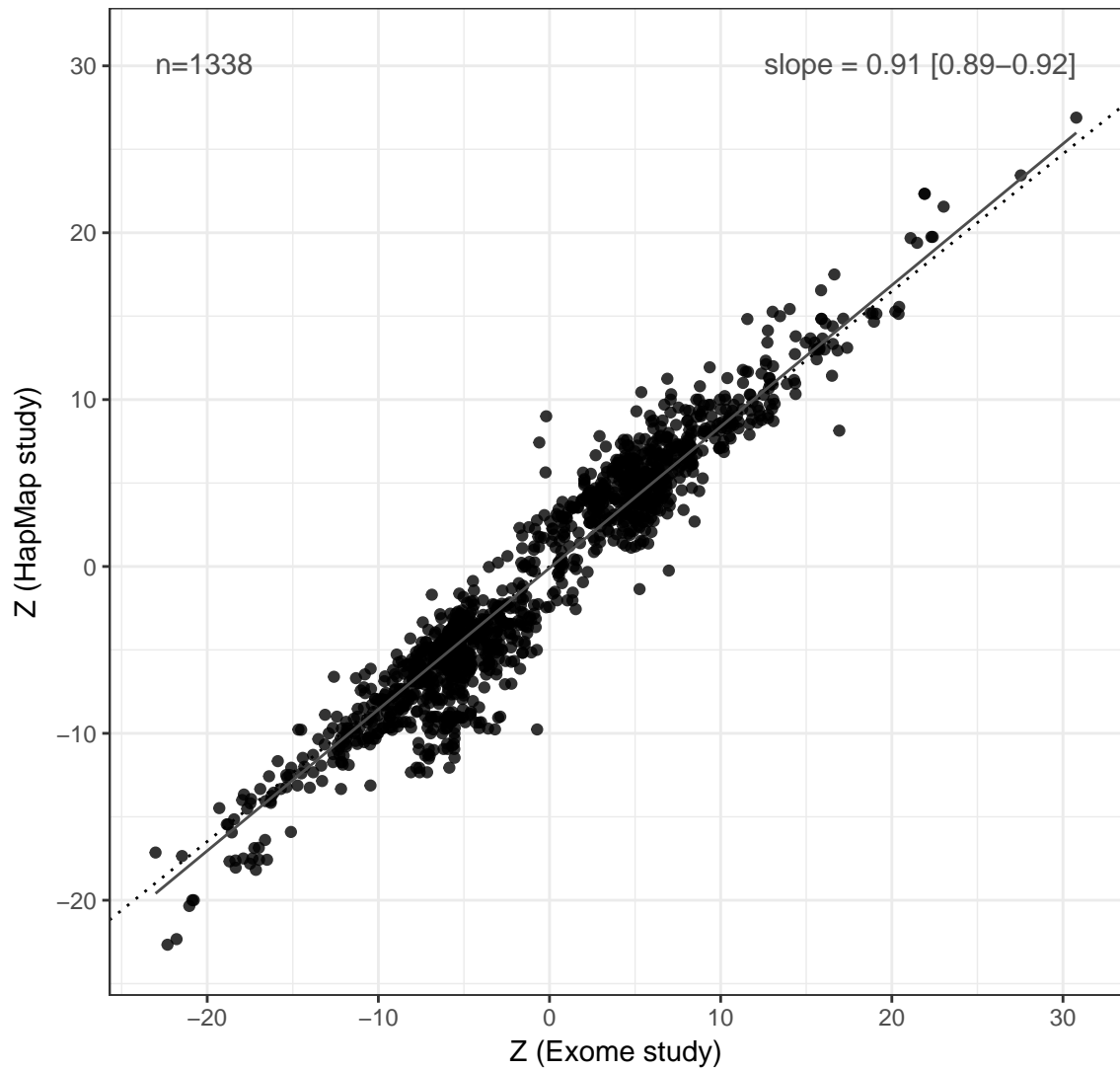

Supplement: S6 Fig — This graph shows the Z-statistics of the exome chip study on the x-axis versus the Z-statistics of SNP-array study on the y-axis. Each dot shows one of the 2′601 variants that had LDmax > 0.1 (LD with one of the top variants in the exome [13] or HapMap study [12]). To make the density more visible, dots have been made transparent. The solid line indicates a linear regression fit, with the slope in the top right corner (including the 95%-confidence interval in brackets). The dashed line represents the ratio between the two median sample sizes 0.82=NHapMap-studyNexome-study=251′647370′529. (PDF) [file pgen.1007371.s006.pdf]

## Candidate locus # 6

## HapMap and imputation results

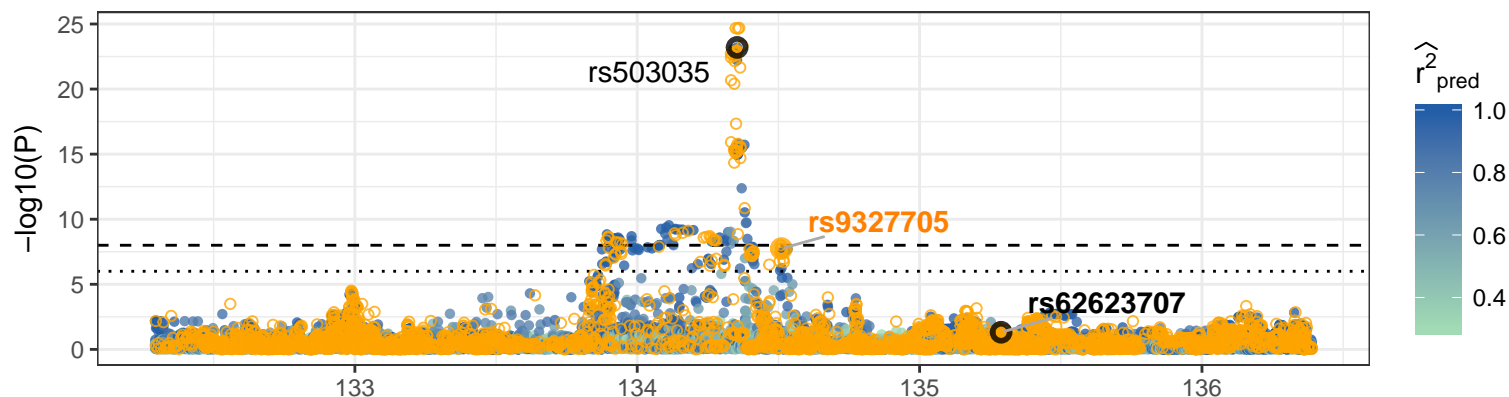

## Genes

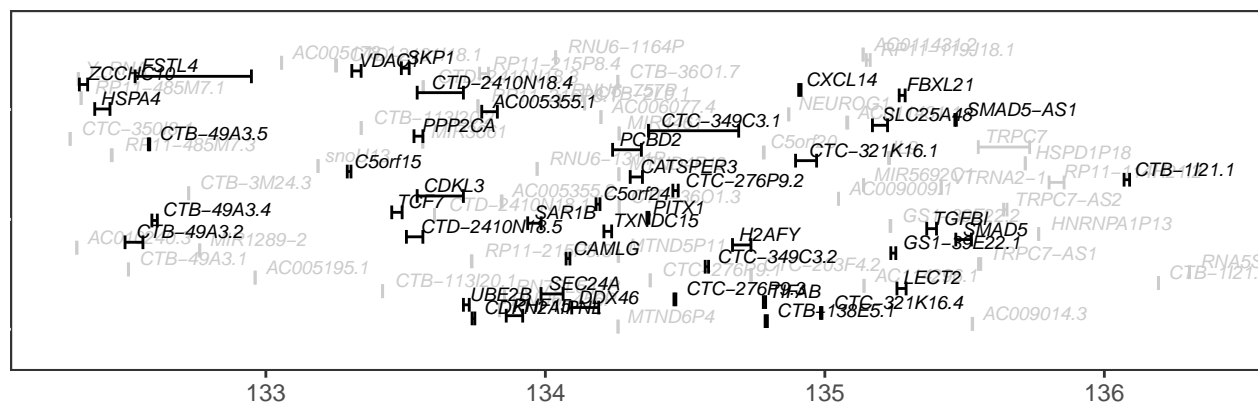

## Exome results

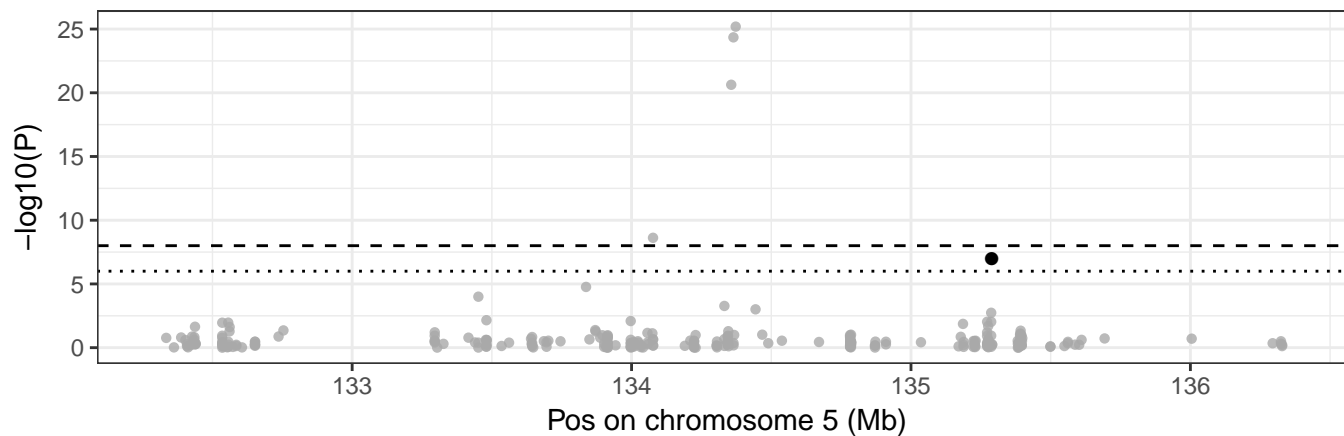

Supplement: S7 Fig — This figure shows three datasets: Results from the HapMap and the exome chip study, and imputed summary statistics. The top window shows HapMap P-values as orange circles and the imputed P-values (using summary statistics imputation) as solid circles, with the colour representing the imputation quality (only r^pred,adj2≥0.3 shown). The bottom window shows exome chip study results as solid, grey dots. Each dot represents the summary statistics of one variant. The x-axis shows the position (in Mb) on a ≥ 2 Mb range and the y-axis the −log10(P)-value. The horizontal line shows the P-value threshold of 10−6 (dotted) and 10−8 (dashed). Top and bottom window have annotated summary statistics: In the bottom window we mark dots as black if it is are part of the 122 reported hits of [13]. In the top window we mark the rs-id of variants that are part of the 122 reported variants of [13] in bold black, and if they are part of the 697 variants of [12] in bold orange font. Variants that are black (plain) are imputed variants (that had the lowest conditional P-value). Variants in orange (plain) are HapMap variants, but were not among the 697 reported hits. Each of the annotated variants is marked for clarity with a bold circle in the respective colour. The genes annotated in the middle window are printed in grey if the gene has a length < 5′000 bp or is an unrecognised gene (RP-). (ZIP) [file pgen.1007371.s007.zip › locuszoomplot/LOCUSZOOM_locusnbr-6_5-134353734_rs503035.pdf]

# Candidate locus # 1

## HapMap and imputation results

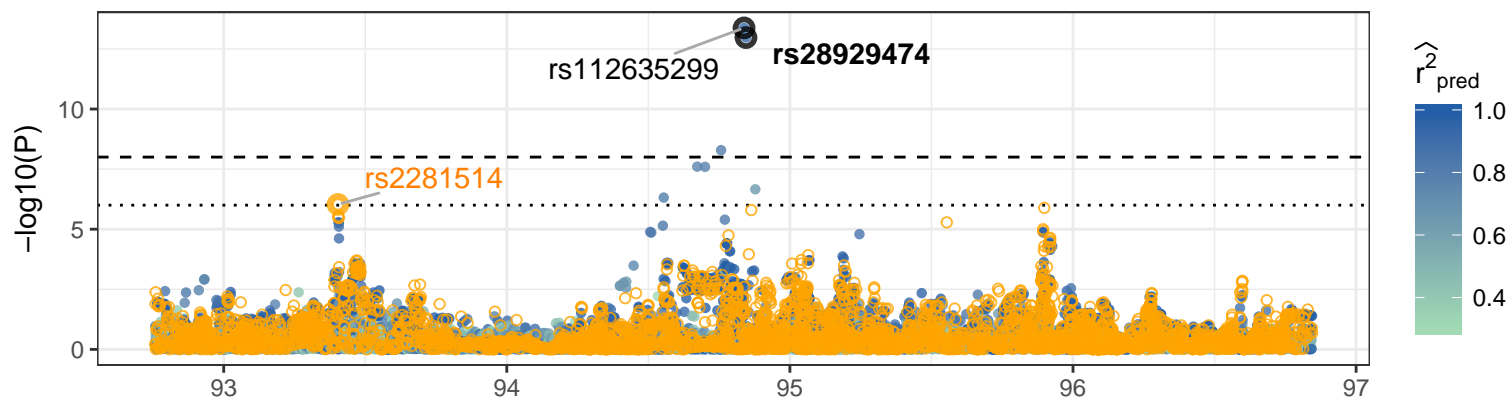

## Genes

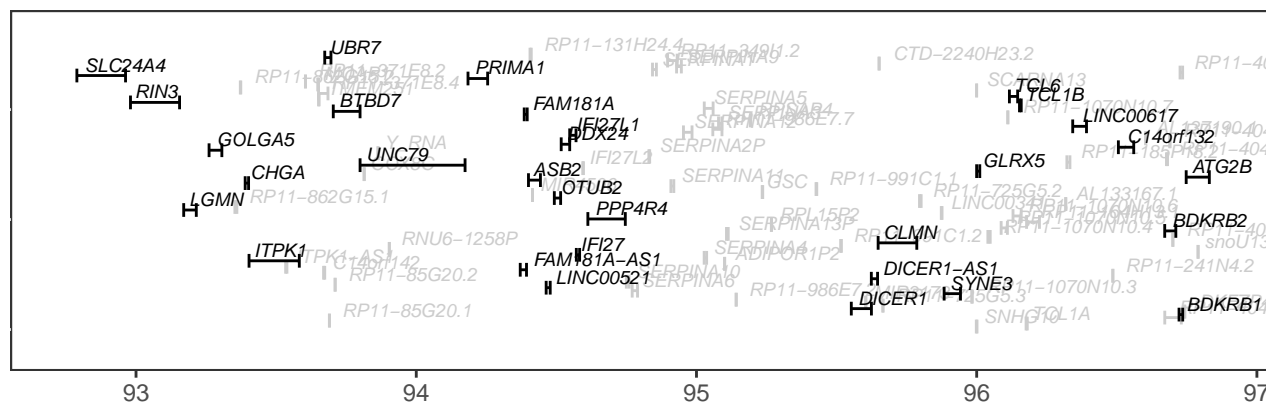

## Exome results

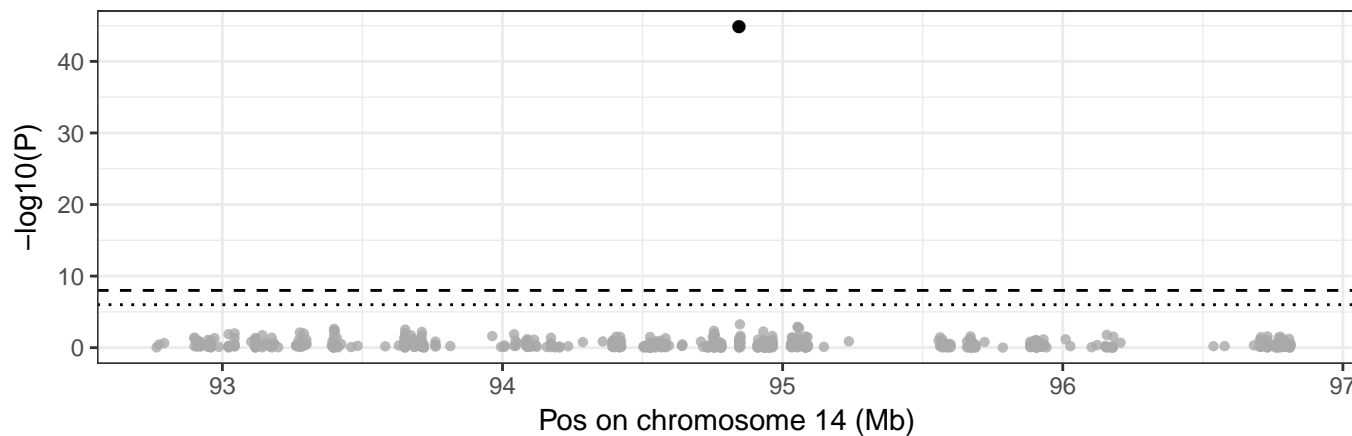

Supplement: S7 Fig — This figure shows three datasets: Results from the HapMap and the exome chip study, and imputed summary statistics. The top window shows HapMap P-values as orange circles and the imputed P-values (using summary statistics imputation) as solid circles, with the colour representing the imputation quality (only r^pred,adj2≥0.3 shown). The bottom window shows exome chip study results as solid, grey dots. Each dot represents the summary statistics of one variant. The x-axis shows the position (in Mb) on a ≥ 2 Mb range and the y-axis the −log10(P)-value. The horizontal line shows the P-value threshold of 10−6 (dotted) and 10−8 (dashed). Top and bottom window have annotated summary statistics: In the bottom window we mark dots as black if it is are part of the 122 reported hits of [13]. In the top window we mark the rs-id of variants that are part of the 122 reported variants of [13] in bold black, and if they are part of the 697 variants of [12] in bold orange font. Variants that are black (plain) are imputed variants (that had the lowest conditional P-value). Variants in orange (plain) are HapMap variants, but were not among the 697 reported hits. Each of the annotated variants is marked for clarity with a bold circle in the respective colour. The genes annotated in the middle window are printed in grey if the gene has a length < 5′000 bp or is an unrecognised gene (RP-). (ZIP) [file pgen.1007371.s007.zip › locuszoomplot/LOCUSZOOM_locusnbr-1_14-94838142_rs112635299.pdf]

## HapMap and imputation results

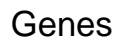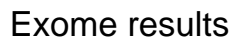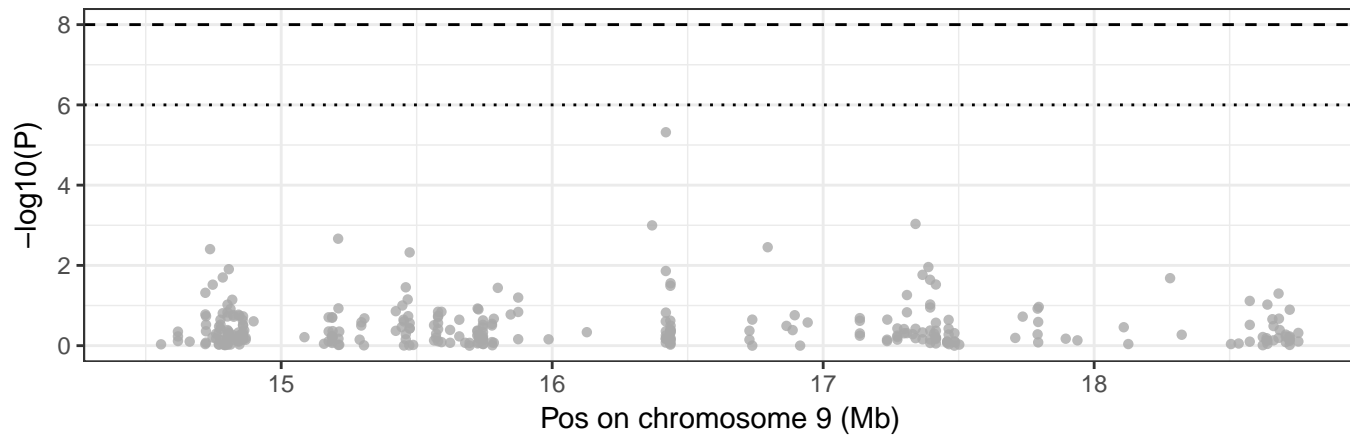

Supplement: S7 Fig — This figure shows three datasets: Results from the HapMap and the exome chip study, and imputed summary statistics. The top window shows HapMap P-values as orange circles and the imputed P-values (using summary statistics imputation) as solid circles, with the colour representing the imputation quality (only r^pred,adj2≥0.3 shown). The bottom window shows exome chip study results as solid, grey dots. Each dot represents the summary statistics of one variant. The x-axis shows the position (in Mb) on a ≥ 2 Mb range and the y-axis the −log10(P)-value. The horizontal line shows the P-value threshold of 10−6 (dotted) and 10−8 (dashed). Top and bottom window have annotated summary statistics: In the bottom window we mark dots as black if it is are part of the 122 reported hits of [13]. In the top window we mark the rs-id of variants that are part of the 122 reported variants of [13] in bold black, and if they are part of the 697 variants of [12] in bold orange font. Variants that are black (plain) are imputed variants (that had the lowest conditional P-value). Variants in orange (plain) are HapMap variants, but were not among the 697 reported hits. Each of the annotated variants is marked for clarity with a bold circle in the respective colour. The genes annotated in the middle window are printed in grey if the gene has a length < 5′000 bp or is an unrecognised gene (RP-). (ZIP) [file pgen.1007371.s007.zip › locuszoomplot/LOCUSZOOM_locusnbr-30_9-16485109_rs61595406;rs79089471;rs58432278.pdf]

# Candidate locus # 26

## HapMap and imputation results

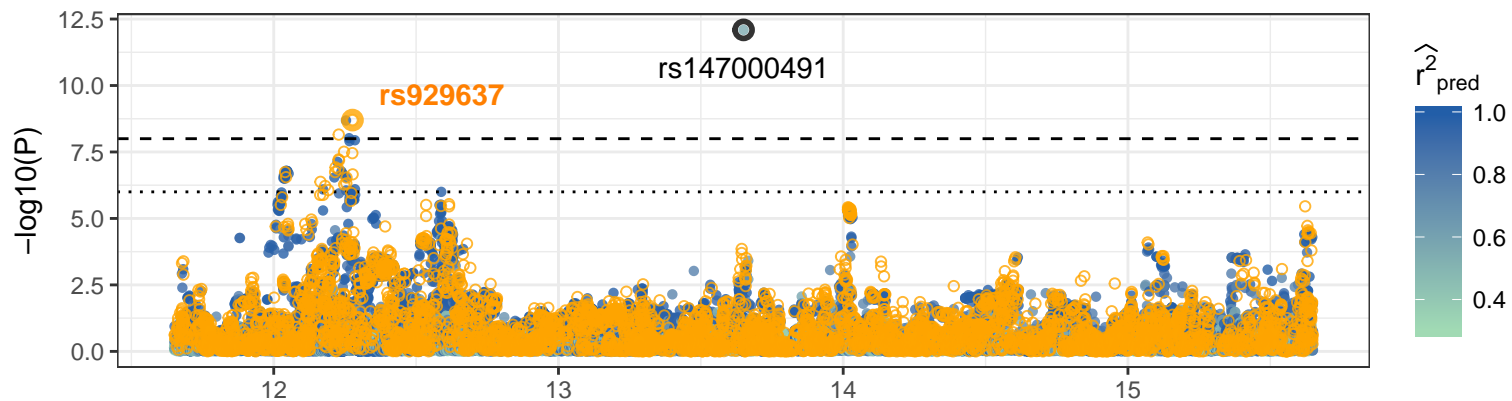

## Genes

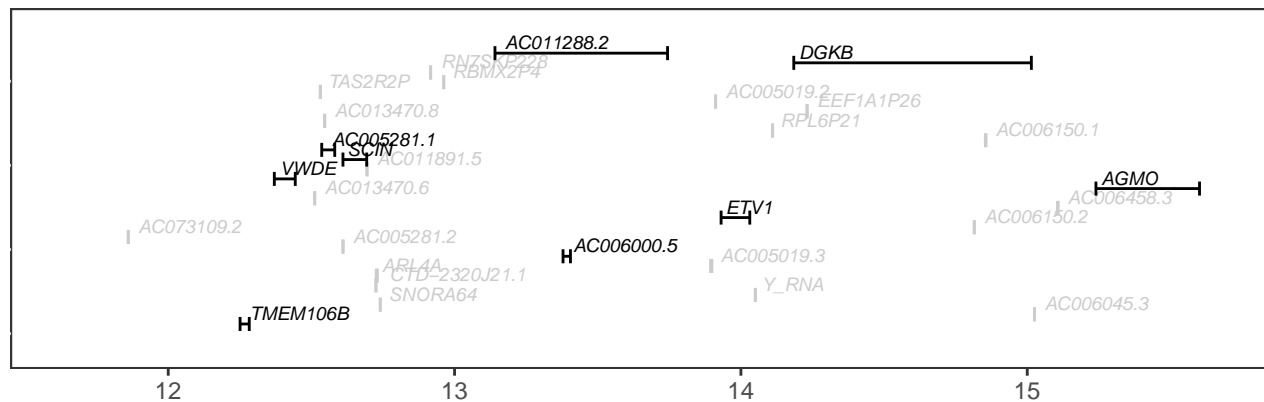

## Exome results

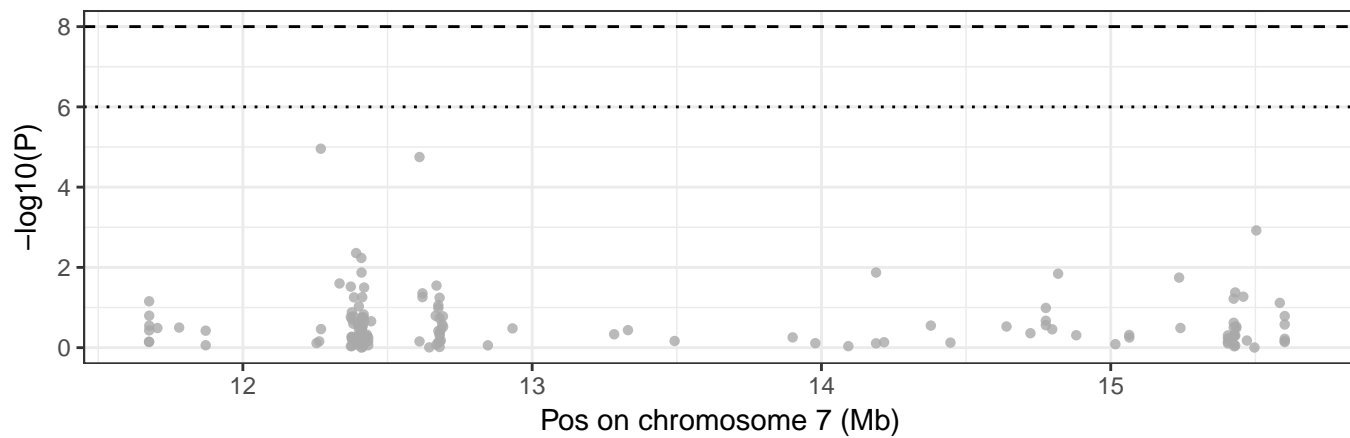

Supplement: S7 Fig — This figure shows three datasets: Results from the HapMap and the exome chip study, and imputed summary statistics. The top window shows HapMap P-values as orange circles and the imputed P-values (using summary statistics imputation) as solid circles, with the colour representing the imputation quality (only r^pred,adj2≥0.3 shown). The bottom window shows exome chip study results as solid, grey dots. Each dot represents the summary statistics of one variant. The x-axis shows the position (in Mb) on a ≥ 2 Mb range and the y-axis the −log10(P)-value. The horizontal line shows the P-value threshold of 10−6 (dotted) and 10−8 (dashed). Top and bottom window have annotated summary statistics: In the bottom window we mark dots as black if it is are part of the 122 reported hits of [13]. In the top window we mark the rs-id of variants that are part of the 122 reported variants of [13] in bold black, and if they are part of the 697 variants of [12] in bold orange font. Variants that are black (plain) are imputed variants (that had the lowest conditional P-value). Variants in orange (plain) are HapMap variants, but were not among the 697 reported hits. Each of the annotated variants is marked for clarity with a bold circle in the respective colour. The genes annotated in the middle window are printed in grey if the gene has a length < 5′000 bp or is an unrecognised gene (RP-). (ZIP) [file pgen.1007371.s007.zip › locuszoomplot/LOCUSZOOM_locusnbr-26_7-13650036_rs147000491.pdf]

# Candidate locus # 9

## HapMap and imputation results

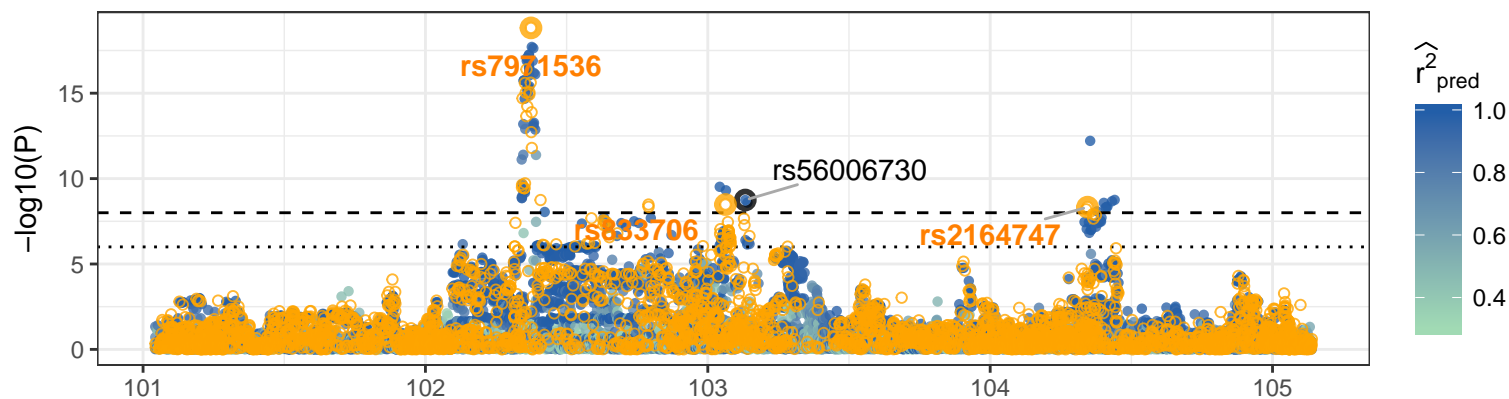

## Genes

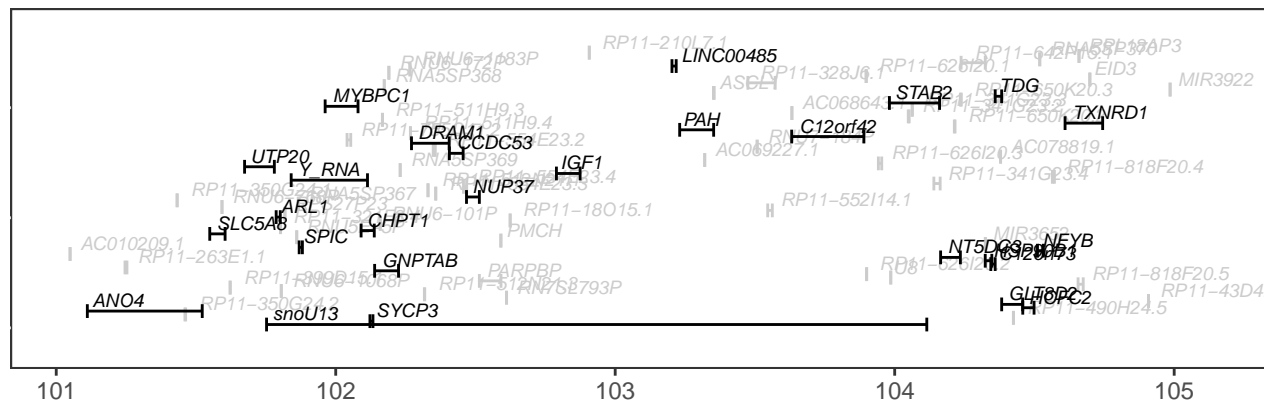

## Exome results

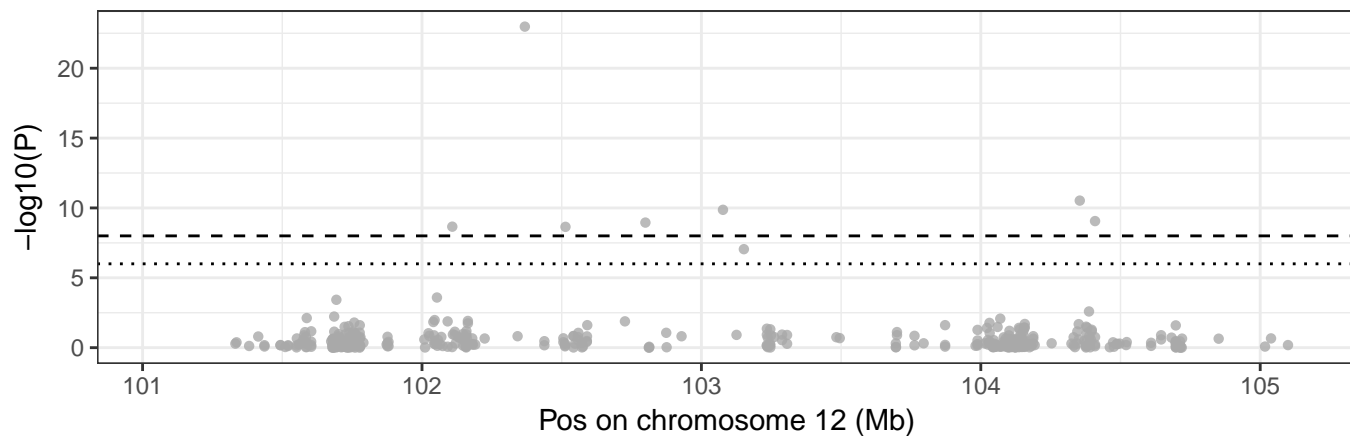

Supplement: S7 Fig — This figure shows three datasets: Results from the HapMap and the exome chip study, and imputed summary statistics. The top window shows HapMap P-values as orange circles and the imputed P-values (using summary statistics imputation) as solid circles, with the colour representing the imputation quality (only r^pred,adj2≥0.3 shown). The bottom window shows exome chip study results as solid, grey dots. Each dot represents the summary statistics of one variant. The x-axis shows the position (in Mb) on a ≥ 2 Mb range and the y-axis the −log10(P)-value. The horizontal line shows the P-value threshold of 10−6 (dotted) and 10−8 (dashed). Top and bottom window have annotated summary statistics: In the bottom window we mark dots as black if it is are part of the 122 reported hits of [13]. In the top window we mark the rs-id of variants that are part of the 122 reported variants of [13] in bold black, and if they are part of the 697 variants of [12] in bold orange font. Variants that are black (plain) are imputed variants (that had the lowest conditional P-value). Variants in orange (plain) are HapMap variants, but were not among the 697 reported hits. Each of the annotated variants is marked for clarity with a bold circle in the respective colour. The genes annotated in the middle window are printed in grey if the gene has a length < 5′000 bp or is an unrecognised gene (RP-). (ZIP) [file pgen.1007371.s007.zip › locuszoomplot/LOCUSZOOM_locusnbr-9_12-103132740_rs56006730.pdf]

## Candidate locus # 8

## HapMap and imputation results

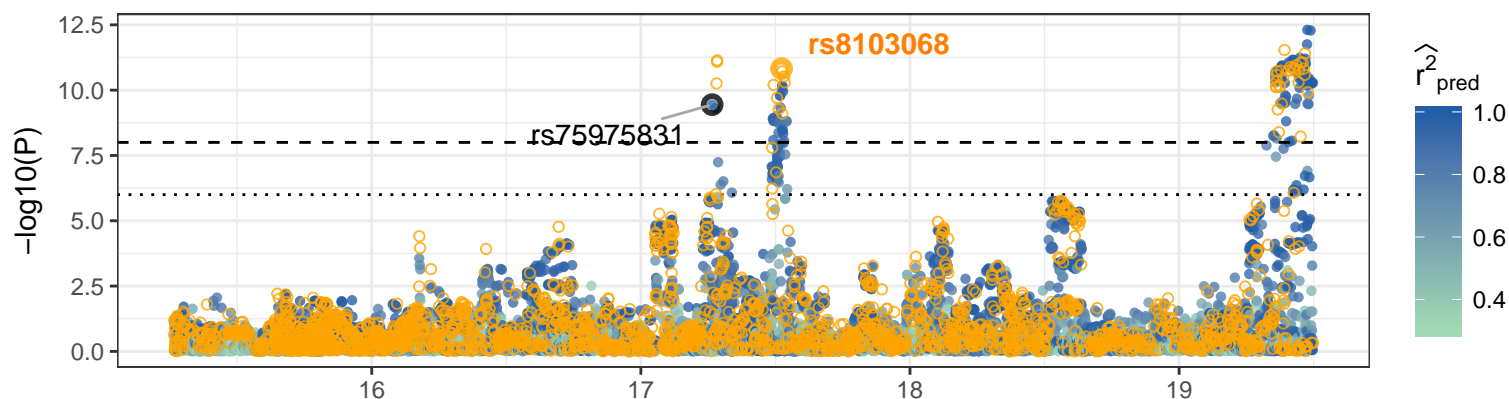

## Genes

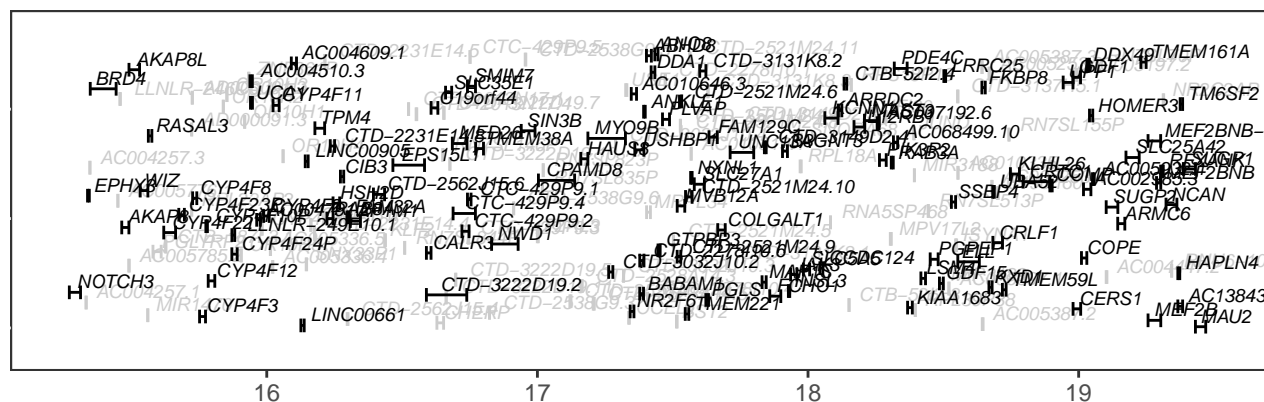

## Exome results

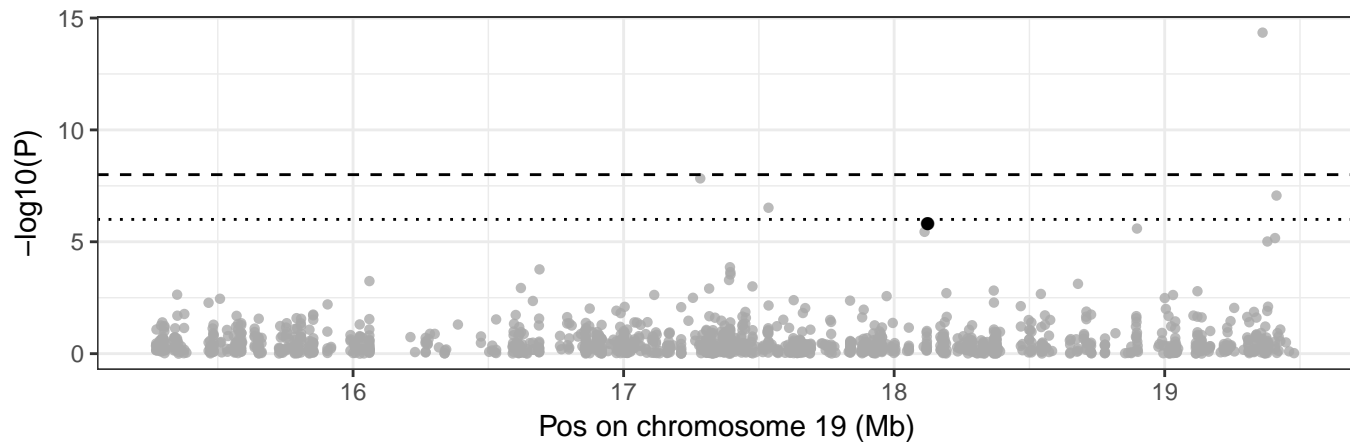

Supplement: S7 Fig — This figure shows three datasets: Results from the HapMap and the exome chip study, and imputed summary statistics. The top window shows HapMap P-values as orange circles and the imputed P-values (using summary statistics imputation) as solid circles, with the colour representing the imputation quality (only r^pred,adj2≥0.3 shown). The bottom window shows exome chip study results as solid, grey dots. Each dot represents the summary statistics of one variant. The x-axis shows the position (in Mb) on a ≥ 2 Mb range and the y-axis the −log10(P)-value. The horizontal line shows the P-value threshold of 10−6 (dotted) and 10−8 (dashed). Top and bottom window have annotated summary statistics: In the bottom window we mark dots as black if it is are part of the 122 reported hits of [13]. In the top window we mark the rs-id of variants that are part of the 122 reported variants of [13] in bold black, and if they are part of the 697 variants of [12] in bold orange font. Variants that are black (plain) are imputed variants (that had the lowest conditional P-value). Variants in orange (plain) are HapMap variants, but were not among the 697 reported hits. Each of the annotated variants is marked for clarity with a bold circle in the respective colour. The genes annotated in the middle window are printed in grey if the gene has a length < 5′000 bp or is an unrecognised gene (RP-). (ZIP) [file pgen.1007371.s007.zip › locuszoomplot/LOCUSZOOM_locusnbr-8_19-17264961_rs75975831.pdf]

# Candidate locus # 14

## HapMap and imputation results

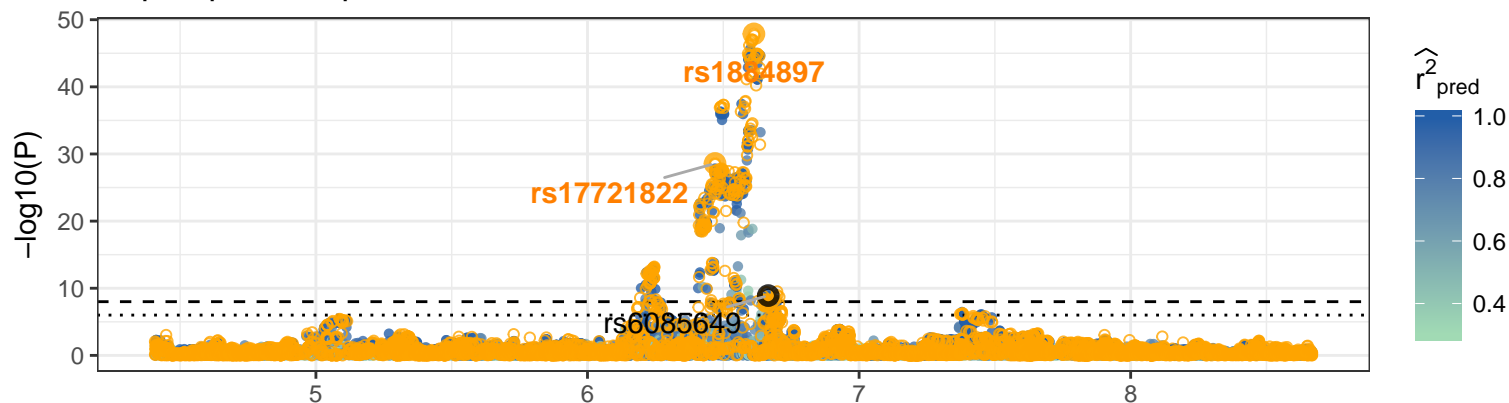

## Genes

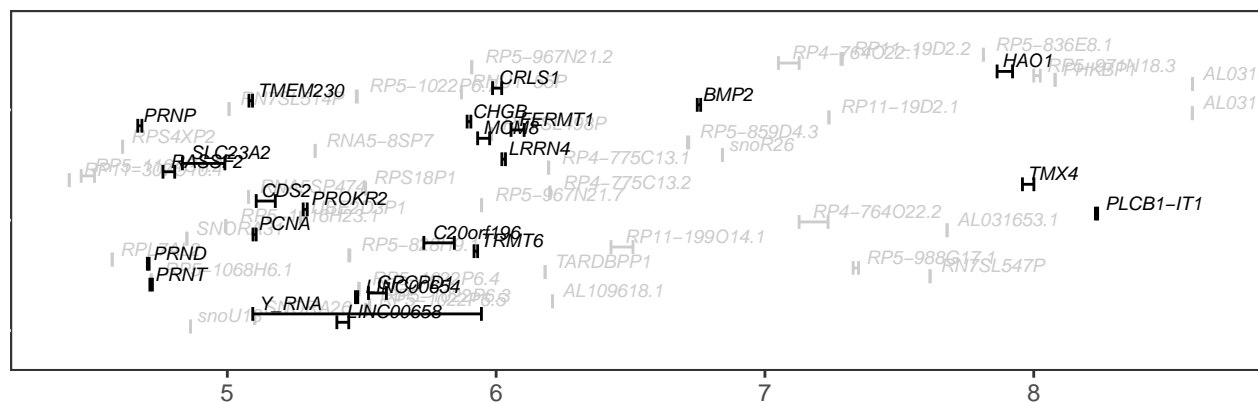

## Exome results

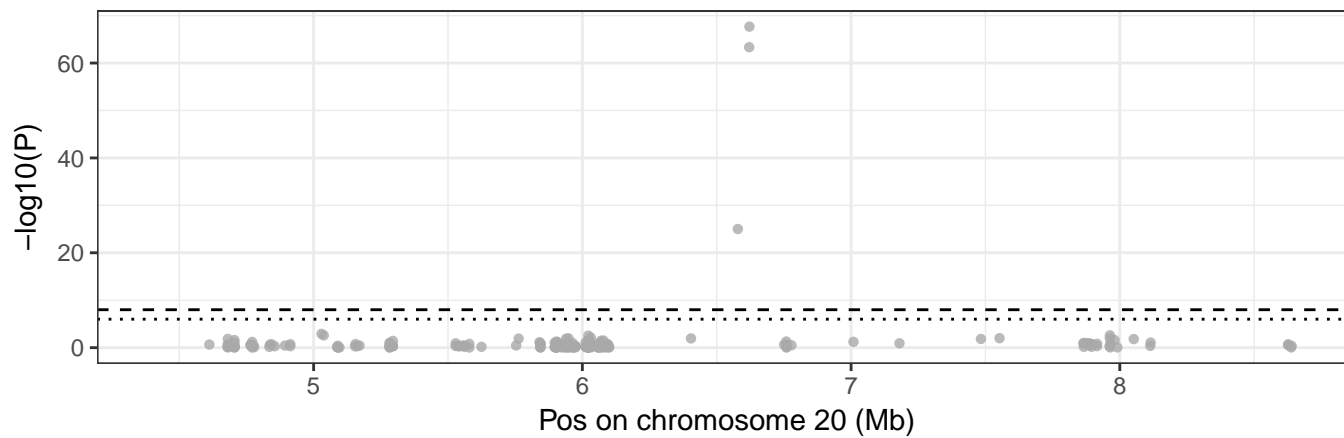

Supplement: S7 Fig — This figure shows three datasets: Results from the HapMap and the exome chip study, and imputed summary statistics. The top window shows HapMap P-values as orange circles and the imputed P-values (using summary statistics imputation) as solid circles, with the colour representing the imputation quality (only r^pred,adj2≥0.3 shown). The bottom window shows exome chip study results as solid, grey dots. Each dot represents the summary statistics of one variant. The x-axis shows the position (in Mb) on a ≥ 2 Mb range and the y-axis the −log10(P)-value. The horizontal line shows the P-value threshold of 10−6 (dotted) and 10−8 (dashed). Top and bottom window have annotated summary statistics: In the bottom window we mark dots as black if it is are part of the 122 reported hits of [13]. In the top window we mark the rs-id of variants that are part of the 122 reported variants of [13] in bold black, and if they are part of the 697 variants of [12] in bold orange font. Variants that are black (plain) are imputed variants (that had the lowest conditional P-value). Variants in orange (plain) are HapMap variants, but were not among the 697 reported hits. Each of the annotated variants is marked for clarity with a bold circle in the respective colour. The genes annotated in the middle window are printed in grey if the gene has a length < 5′000 bp or is an unrecognised gene (RP-). (ZIP) [file pgen.1007371.s007.zip › locuszoomplot/LOCUSZOOM_locusnbr-14_20-6665532_rs6085649.pdf]

# Candidate locus # 22

## HapMap and imputation results

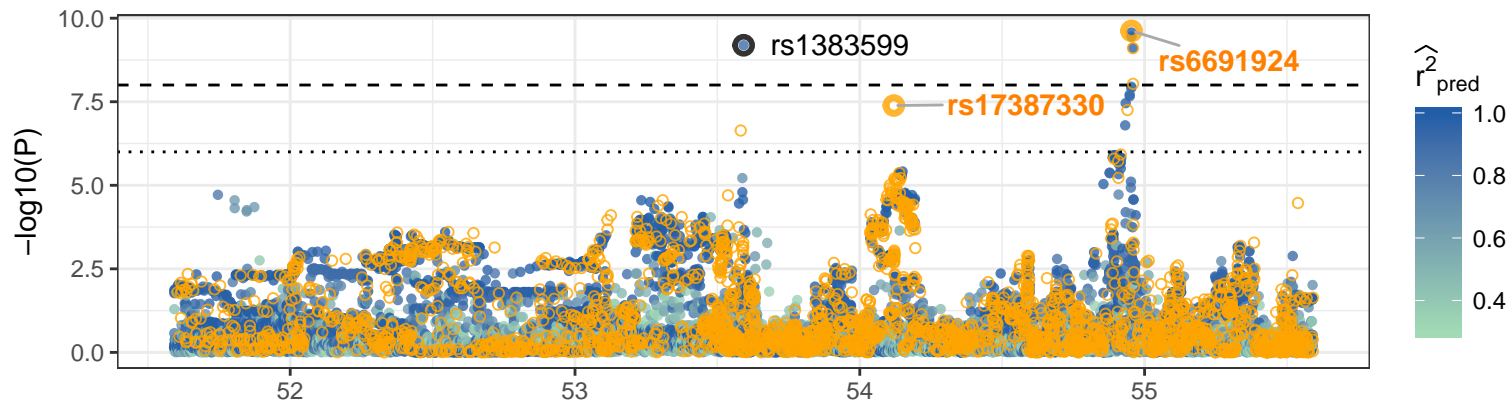

## Genes

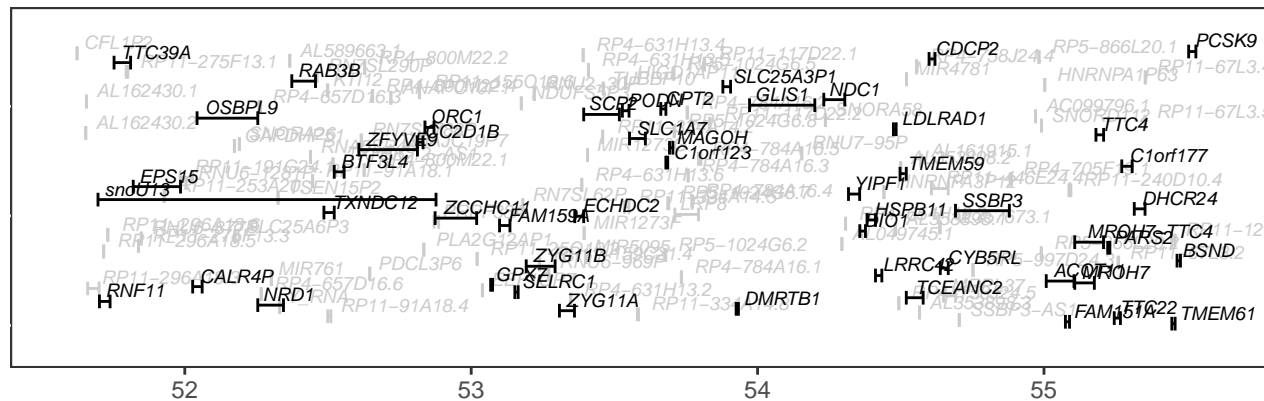

## Exome results

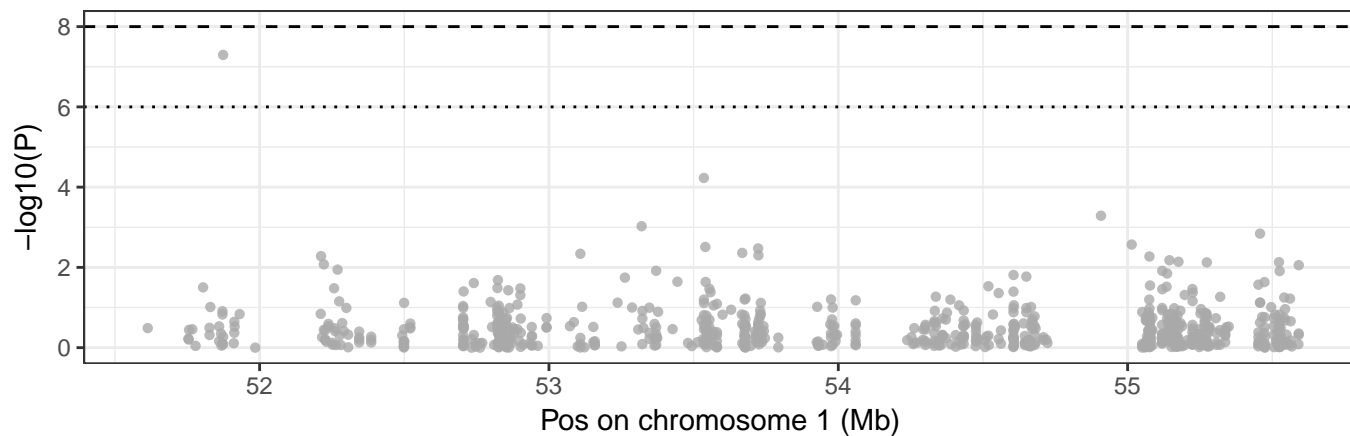

Supplement: S7 Fig — This figure shows three datasets: Results from the HapMap and the exome chip study, and imputed summary statistics. The top window shows HapMap P-values as orange circles and the imputed P-values (using summary statistics imputation) as solid circles, with the colour representing the imputation quality (only r^pred,adj2≥0.3 shown). The bottom window shows exome chip study results as solid, grey dots. Each dot represents the summary statistics of one variant. The x-axis shows the position (in Mb) on a ≥ 2 Mb range and the y-axis the −log10(P)-value. The horizontal line shows the P-value threshold of 10−6 (dotted) and 10−8 (dashed). Top and bottom window have annotated summary statistics: In the bottom window we mark dots as black if it is are part of the 122 reported hits of [13]. In the top window we mark the rs-id of variants that are part of the 122 reported variants of [13] in bold black, and if they are part of the 697 variants of [12] in bold orange font. Variants that are black (plain) are imputed variants (that had the lowest conditional P-value). Variants in orange (plain) are HapMap variants, but were not among the 697 reported hits. Each of the annotated variants is marked for clarity with a bold circle in the respective colour. The genes annotated in the middle window are printed in grey if the gene has a length < 5′000 bp or is an unrecognised gene (RP-). (ZIP) [file pgen.1007371.s007.zip › locuszoomplot/LOCUSZOOM_locusnbr-22_1-53592123_rs1383599.pdf]

## Candidate locus # 16

## HapMap and imputation results

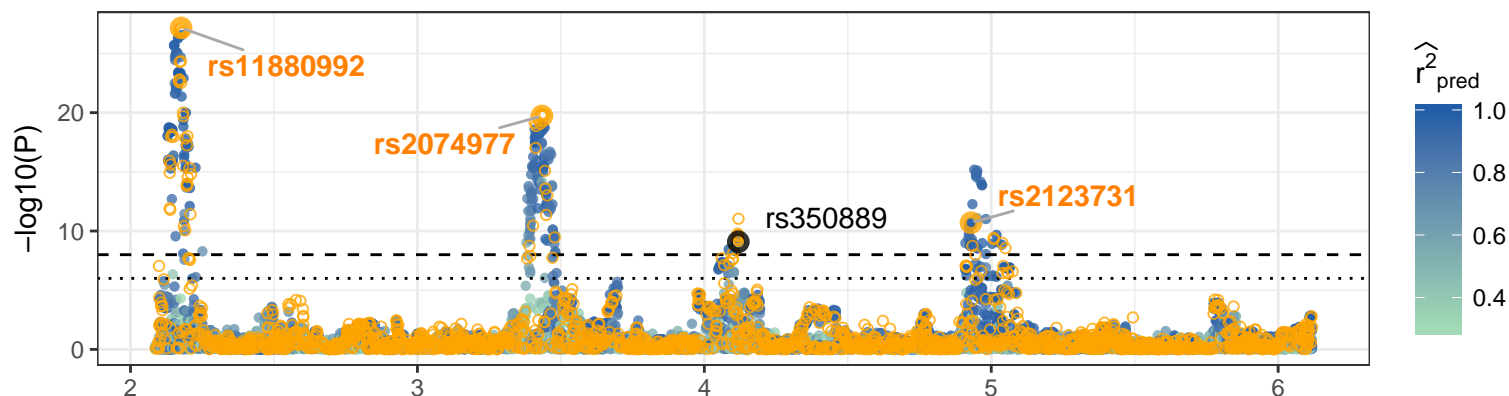

## Genes

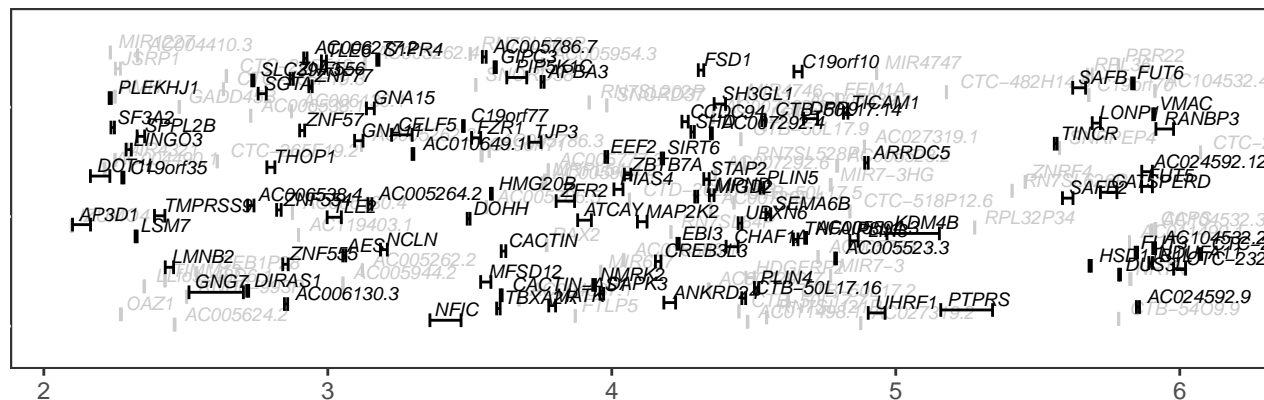

## Exome results

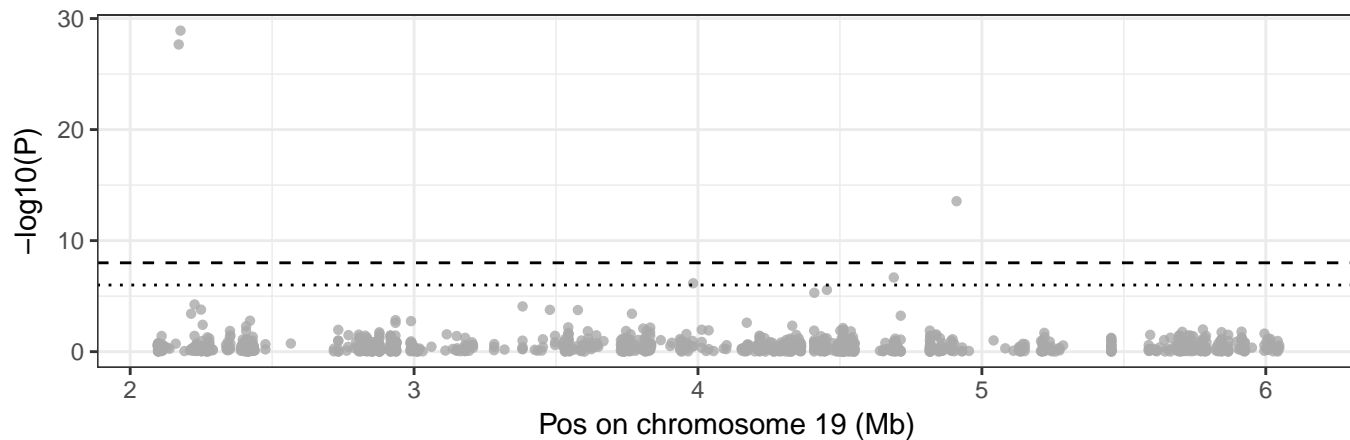

Supplement: S7 Fig — This figure shows three datasets: Results from the HapMap and the exome chip study, and imputed summary statistics. The top window shows HapMap P-values as orange circles and the imputed P-values (using summary statistics imputation) as solid circles, with the colour representing the imputation quality (only r^pred,adj2≥0.3 shown). The bottom window shows exome chip study results as solid, grey dots. Each dot represents the summary statistics of one variant. The x-axis shows the position (in Mb) on a ≥ 2 Mb range and the y-axis the −log10(P)-value. The horizontal line shows the P-value threshold of 10−6 (dotted) and 10−8 (dashed). Top and bottom window have annotated summary statistics: In the bottom window we mark dots as black if it is are part of the 122 reported hits of [13]. In the top window we mark the rs-id of variants that are part of the 122 reported variants of [13] in bold black, and if they are part of the 697 variants of [12] in bold orange font. Variants that are black (plain) are imputed variants (that had the lowest conditional P-value). Variants in orange (plain) are HapMap variants, but were not among the 697 reported hits. Each of the annotated variants is marked for clarity with a bold circle in the respective colour. The genes annotated in the middle window are printed in grey if the gene has a length < 5′000 bp or is an unrecognised gene (RP-). (ZIP) [file pgen.1007371.s007.zip › locuszoomplot/LOCUSZOOM_locusnbr-16_19-4118481_rs350889.pdf]

# Candidate locus # 10

## HapMap and imputation results

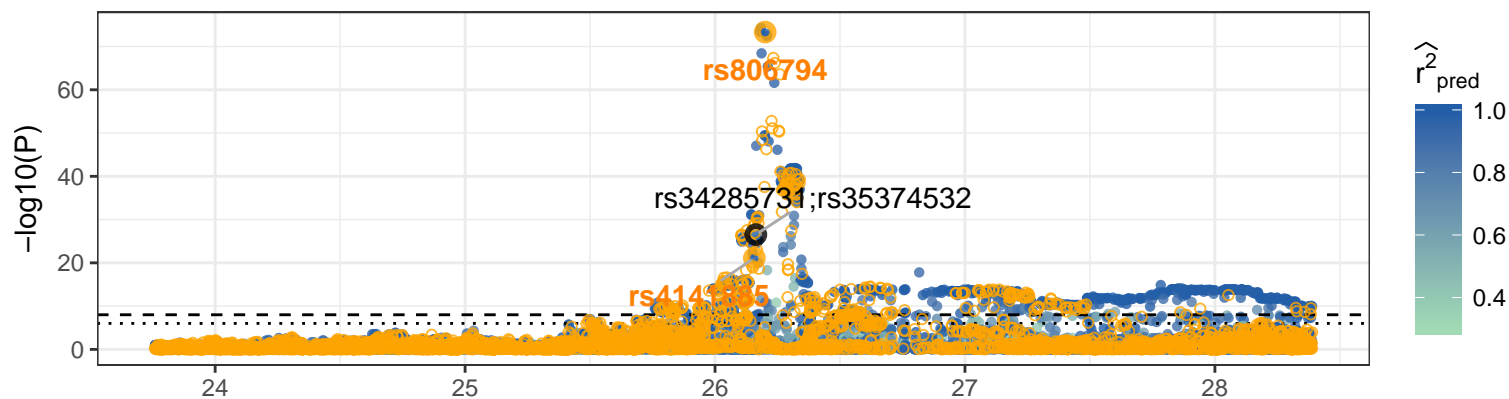

## Genes

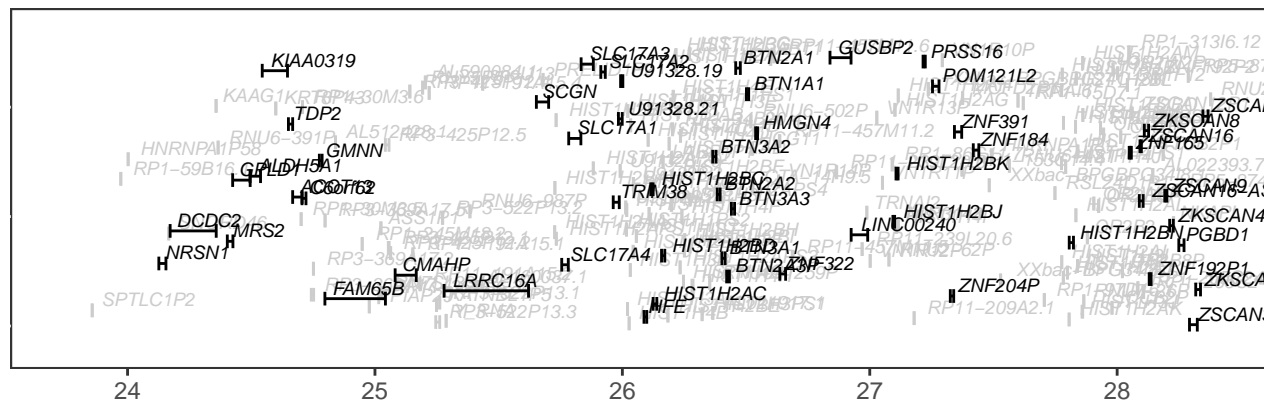

## Exome results

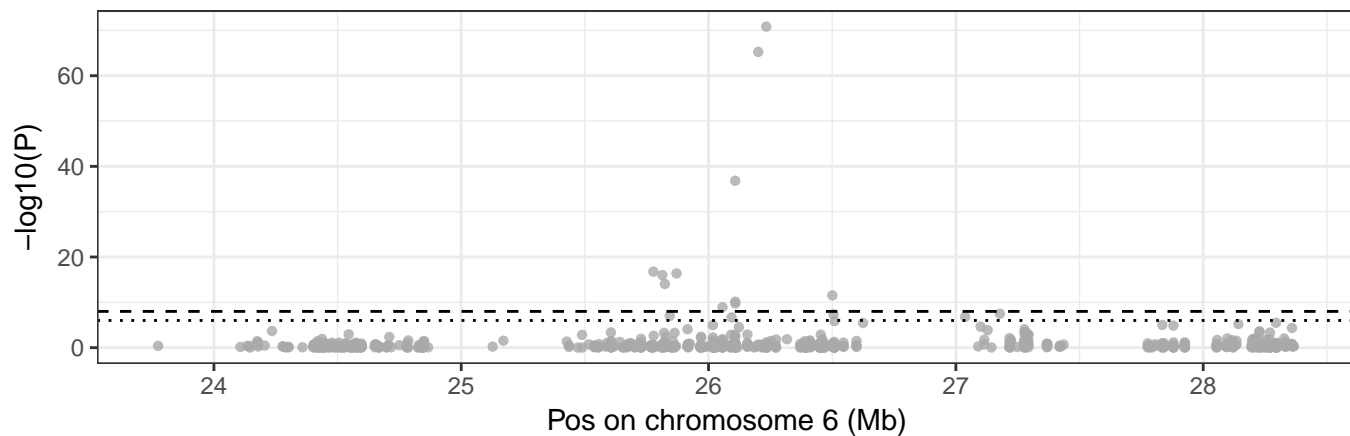

Supplement: S7 Fig — This figure shows three datasets: Results from the HapMap and the exome chip study, and imputed summary statistics. The top window shows HapMap P-values as orange circles and the imputed P-values (using summary statistics imputation) as solid circles, with the colour representing the imputation quality (only r^pred,adj2≥0.3 shown). The bottom window shows exome chip study results as solid, grey dots. Each dot represents the summary statistics of one variant. The x-axis shows the position (in Mb) on a ≥ 2 Mb range and the y-axis the −log10(P)-value. The horizontal line shows the P-value threshold of 10−6 (dotted) and 10−8 (dashed). Top and bottom window have annotated summary statistics: In the bottom window we mark dots as black if it is are part of the 122 reported hits of [13]. In the top window we mark the rs-id of variants that are part of the 122 reported variants of [13] in bold black, and if they are part of the 697 variants of [12] in bold orange font. Variants that are black (plain) are imputed variants (that had the lowest conditional P-value). Variants in orange (plain) are HapMap variants, but were not among the 697 reported hits. Each of the annotated variants is marked for clarity with a bold circle in the respective colour. The genes annotated in the middle window are printed in grey if the gene has a length < 5′000 bp or is an unrecognised gene (RP-). (ZIP) [file pgen.1007371.s007.zip › locuszoomplot/LOCUSZOOM_locusnbr-10_6-26163345_rs34285731;rs35374532.pdf]

## Candidate locus # 29

## HapMap and imputation results

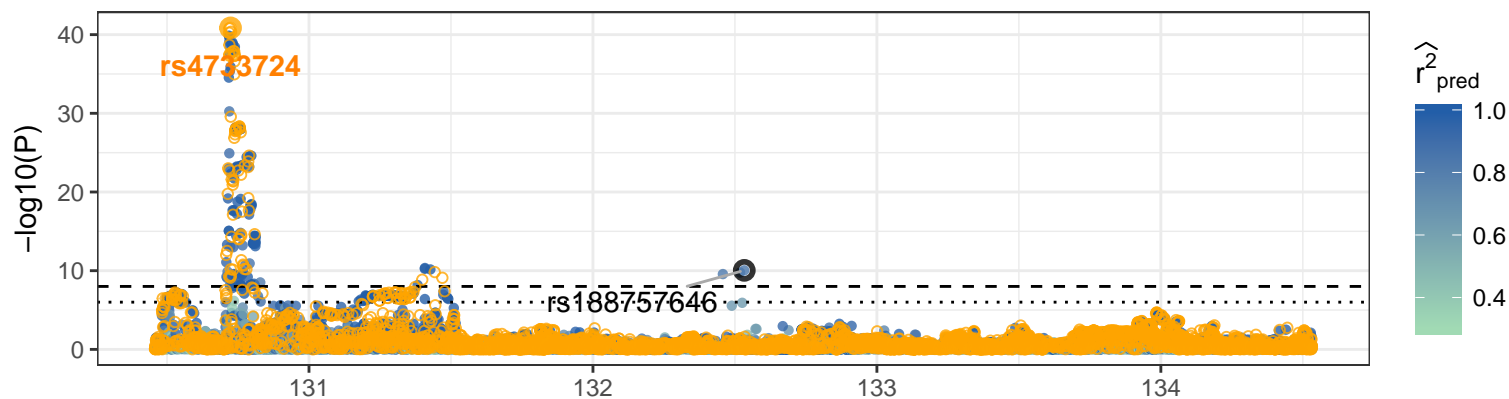

## Genes

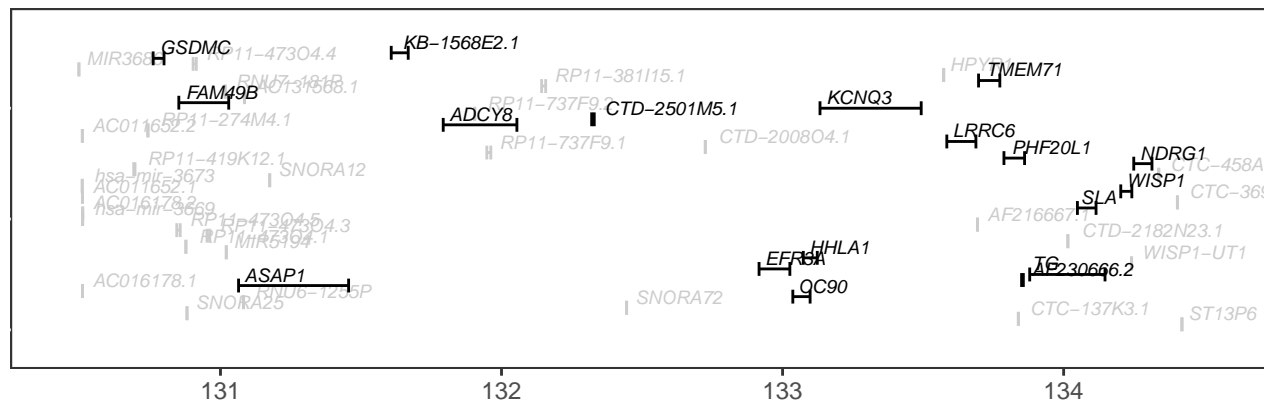

## Exome results

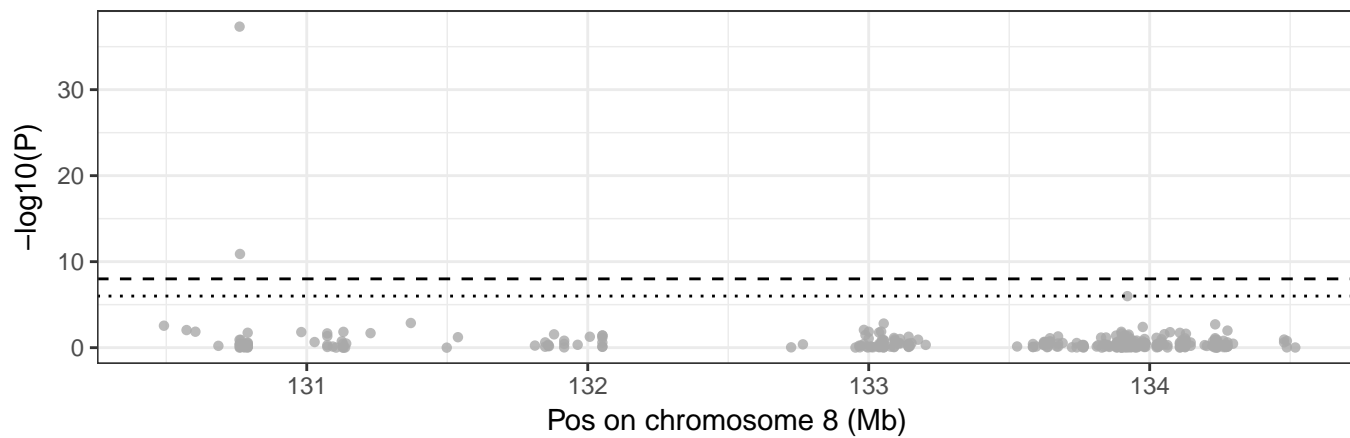

Supplement: S7 Fig — This figure shows three datasets: Results from the HapMap and the exome chip study, and imputed summary statistics. The top window shows HapMap P-values as orange circles and the imputed P-values (using summary statistics imputation) as solid circles, with the colour representing the imputation quality (only r^pred,adj2≥0.3 shown). The bottom window shows exome chip study results as solid, grey dots. Each dot represents the summary statistics of one variant. The x-axis shows the position (in Mb) on a ≥ 2 Mb range and the y-axis the −log10(P)-value. The horizontal line shows the P-value threshold of 10−6 (dotted) and 10−8 (dashed). Top and bottom window have annotated summary statistics: In the bottom window we mark dots as black if it is are part of the 122 reported hits of [13]. In the top window we mark the rs-id of variants that are part of the 122 reported variants of [13] in bold black, and if they are part of the 697 variants of [12] in bold orange font. Variants that are black (plain) are imputed variants (that had the lowest conditional P-value). Variants in orange (plain) are HapMap variants, but were not among the 697 reported hits. Each of the annotated variants is marked for clarity with a bold circle in the respective colour. The genes annotated in the middle window are printed in grey if the gene has a length < 5′000 bp or is an unrecognised gene (RP-). (ZIP) [file pgen.1007371.s007.zip › locuszoomplot/LOCUSZOOM_locusnbr-29_8-132532975_rs188757646.pdf]

## Candidate locus # 15

## HapMap and imputation results

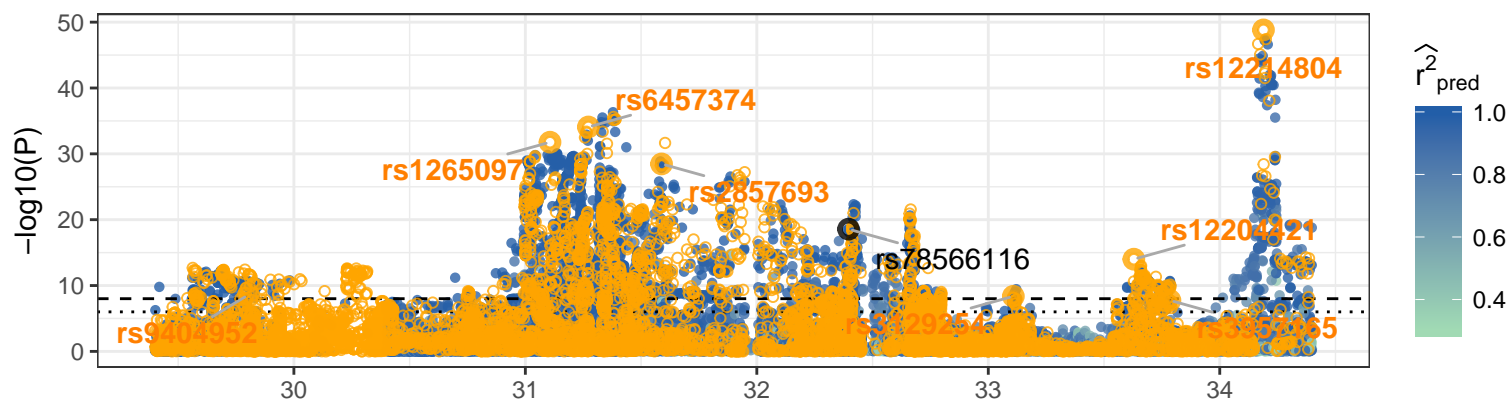

## Genes

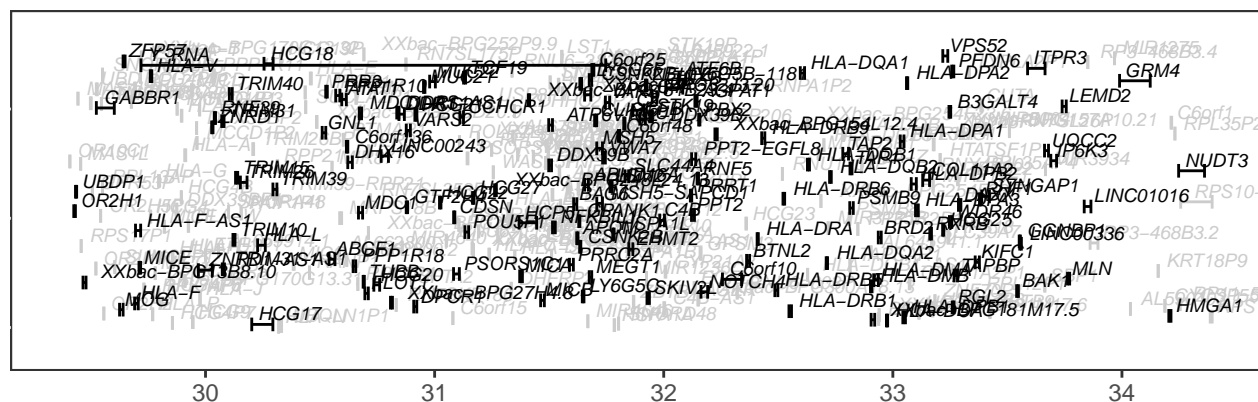

## Exome results

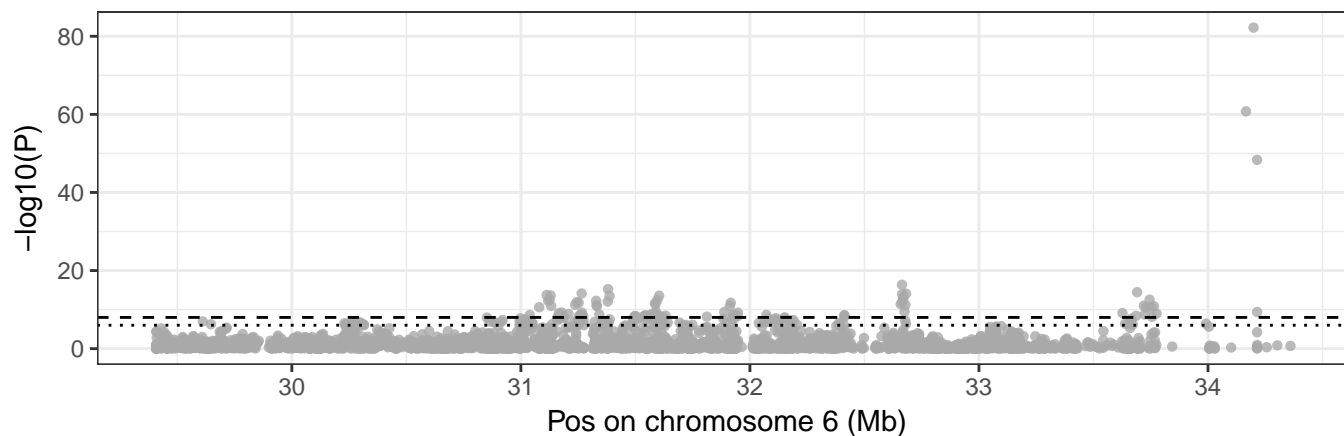

Supplement: S7 Fig — This figure shows three datasets: Results from the HapMap and the exome chip study, and imputed summary statistics. The top window shows HapMap P-values as orange circles and the imputed P-values (using summary statistics imputation) as solid circles, with the colour representing the imputation quality (only r^pred,adj2≥0.3 shown). The bottom window shows exome chip study results as solid, grey dots. Each dot represents the summary statistics of one variant. The x-axis shows the position (in Mb) on a ≥ 2 Mb range and the y-axis the −log10(P)-value. The horizontal line shows the P-value threshold of 10−6 (dotted) and 10−8 (dashed). Top and bottom window have annotated summary statistics: In the bottom window we mark dots as black if it is are part of the 122 reported hits of [13]. In the top window we mark the rs-id of variants that are part of the 122 reported variants of [13] in bold black, and if they are part of the 697 variants of [12] in bold orange font. Variants that are black (plain) are imputed variants (that had the lowest conditional P-value). Variants in orange (plain) are HapMap variants, but were not among the 697 reported hits. Each of the annotated variants is marked for clarity with a bold circle in the respective colour. The genes annotated in the middle window are printed in grey if the gene has a length < 5′000 bp or is an unrecognised gene (RP-). (ZIP) [file pgen.1007371.s007.zip › locuszoomplot/LOCUSZOOM_locusnbr-15_6-32396146_rs78566116.pdf]

# Candidate locus # 32

## HapMap and imputation results

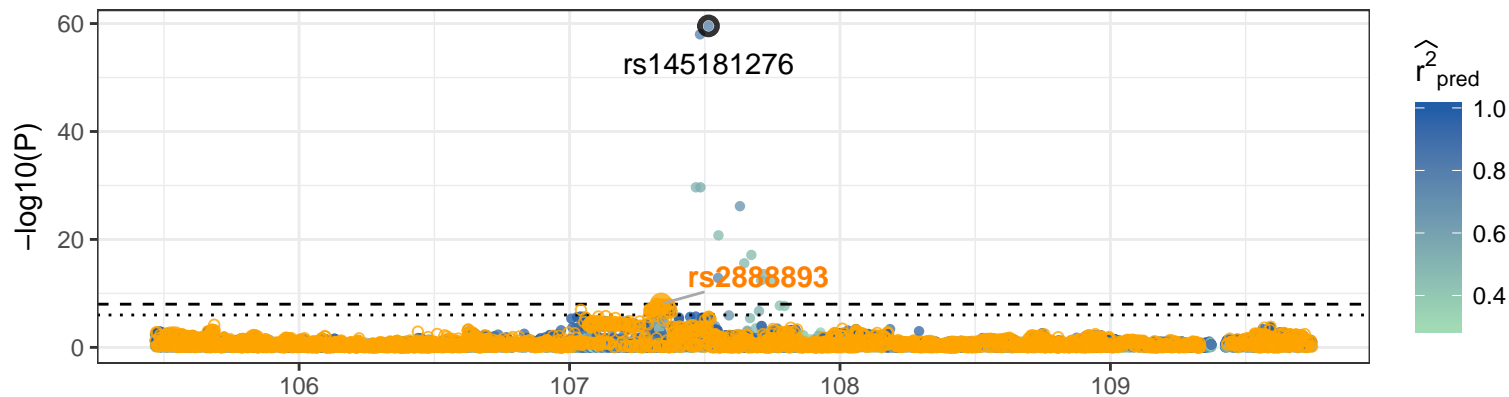

## Genes

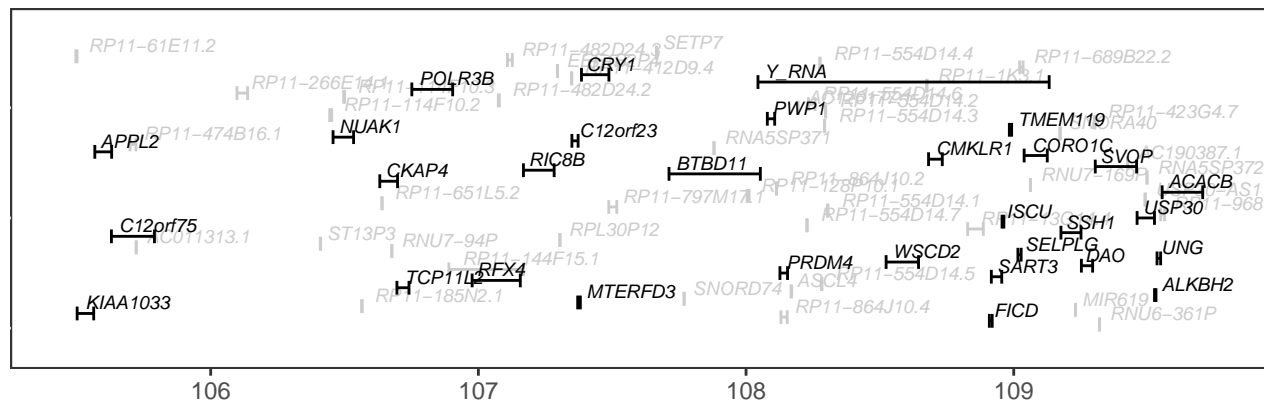

## Exome results

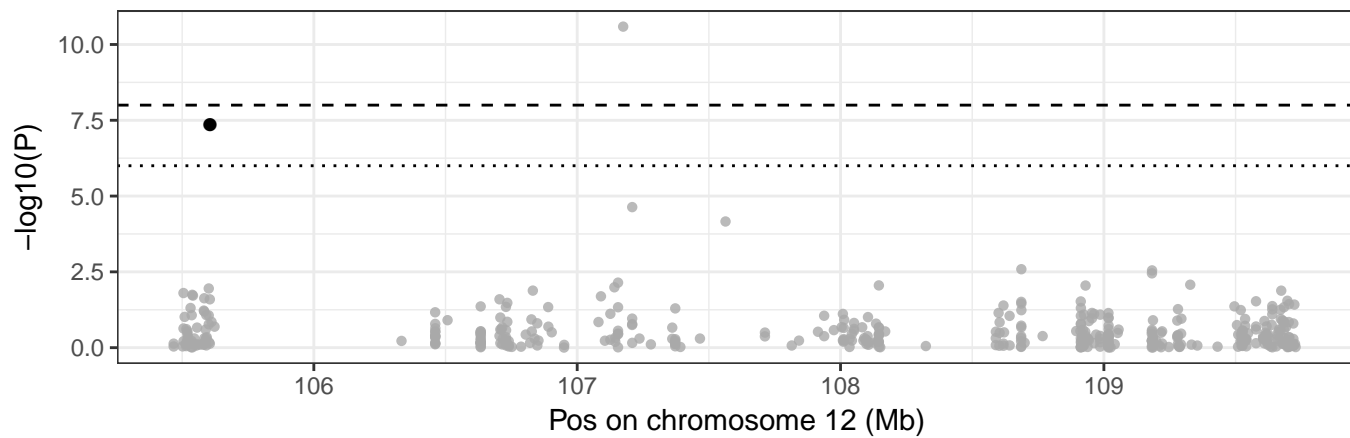

Supplement: S7 Fig — This figure shows three datasets: Results from the HapMap and the exome chip study, and imputed summary statistics. The top window shows HapMap P-values as orange circles and the imputed P-values (using summary statistics imputation) as solid circles, with the colour representing the imputation quality (only r^pred,adj2≥0.3 shown). The bottom window shows exome chip study results as solid, grey dots. Each dot represents the summary statistics of one variant. The x-axis shows the position (in Mb) on a ≥ 2 Mb range and the y-axis the −log10(P)-value. The horizontal line shows the P-value threshold of 10−6 (dotted) and 10−8 (dashed). Top and bottom window have annotated summary statistics: In the bottom window we mark dots as black if it is are part of the 122 reported hits of [13]. In the top window we mark the rs-id of variants that are part of the 122 reported variants of [13] in bold black, and if they are part of the 697 variants of [12] in bold orange font. Variants that are black (plain) are imputed variants (that had the lowest conditional P-value). Variants in orange (plain) are HapMap variants, but were not among the 697 reported hits. Each of the annotated variants is marked for clarity with a bold circle in the respective colour. The genes annotated in the middle window are printed in grey if the gene has a length < 5′000 bp or is an unrecognised gene (RP-). (ZIP) [file pgen.1007371.s007.zip › locuszoomplot/LOCUSZOOM_locusnbr-32_12-107513782_rs145181276.pdf]

## Candidate locus # 19

## HapMap and imputation results

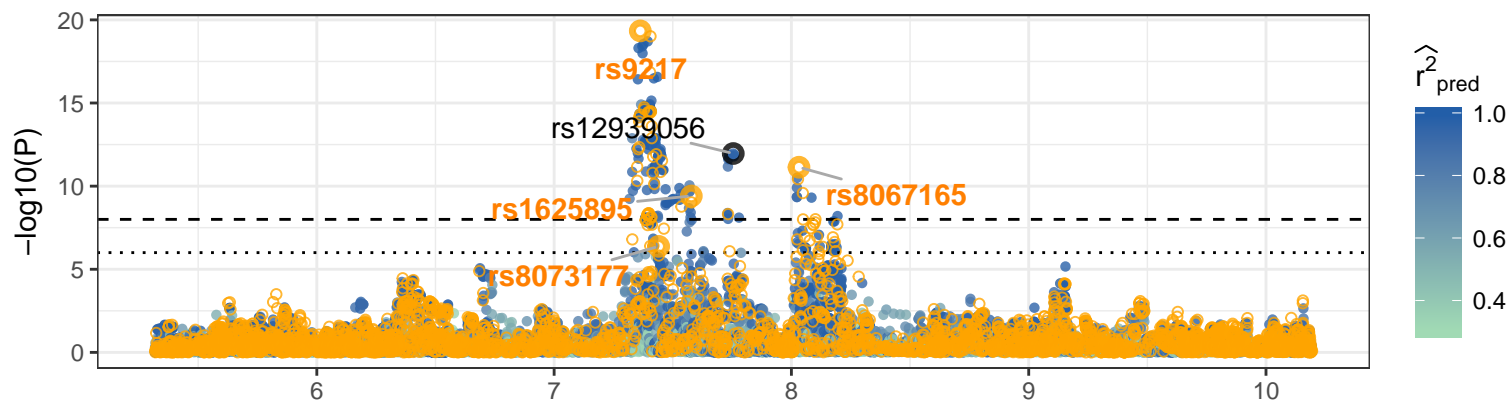

## Genes

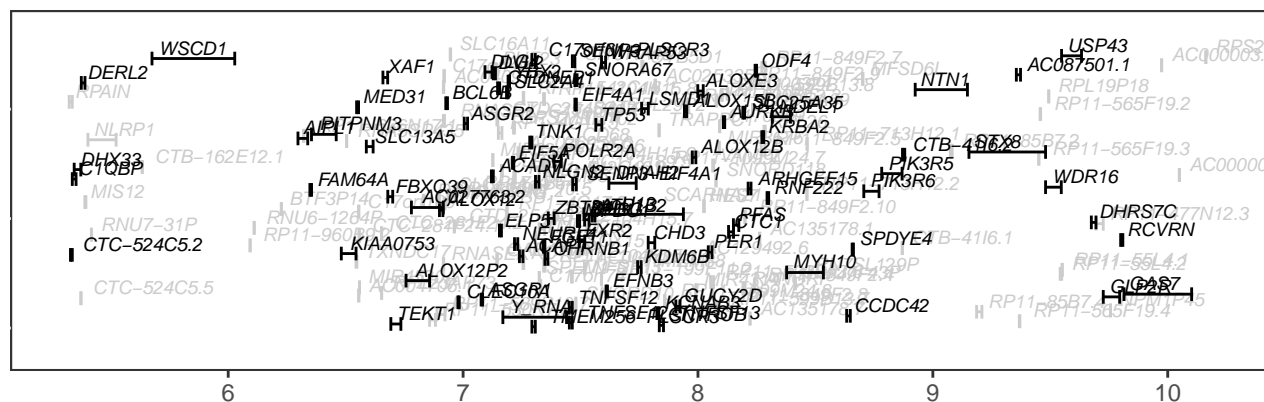

## Exome results

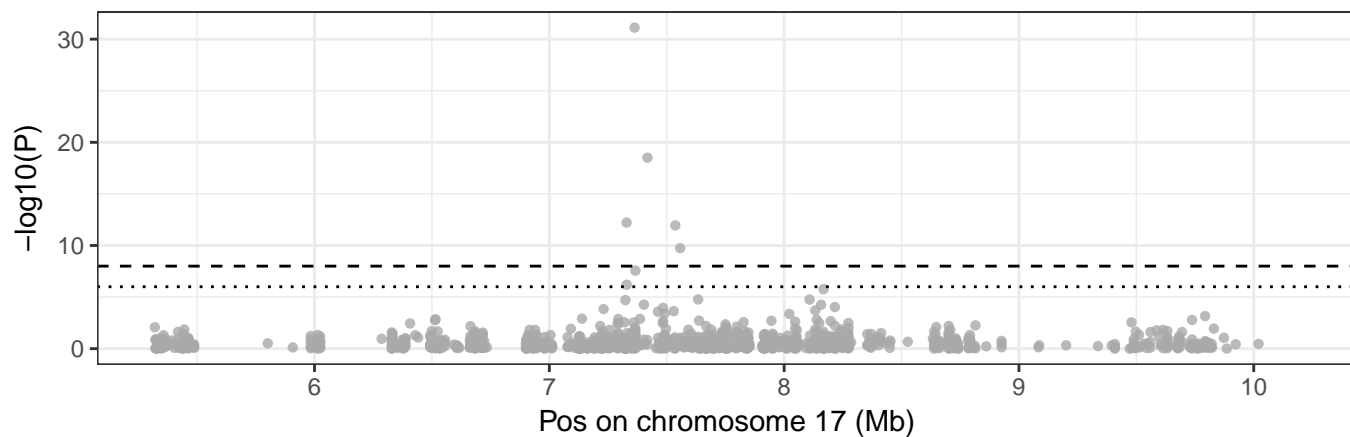

Supplement: S7 Fig — This figure shows three datasets: Results from the HapMap and the exome chip study, and imputed summary statistics. The top window shows HapMap P-values as orange circles and the imputed P-values (using summary statistics imputation) as solid circles, with the colour representing the imputation quality (only r^pred,adj2≥0.3 shown). The bottom window shows exome chip study results as solid, grey dots. Each dot represents the summary statistics of one variant. The x-axis shows the position (in Mb) on a ≥ 2 Mb range and the y-axis the −log10(P)-value. The horizontal line shows the P-value threshold of 10−6 (dotted) and 10−8 (dashed). Top and bottom window have annotated summary statistics: In the bottom window we mark dots as black if it is are part of the 122 reported hits of [13]. In the top window we mark the rs-id of variants that are part of the 122 reported variants of [13] in bold black, and if they are part of the 697 variants of [12] in bold orange font. Variants that are black (plain) are imputed variants (that had the lowest conditional P-value). Variants in orange (plain) are HapMap variants, but were not among the 697 reported hits. Each of the annotated variants is marked for clarity with a bold circle in the respective colour. The genes annotated in the middle window are printed in grey if the gene has a length < 5′000 bp or is an unrecognised gene (RP-). (ZIP) [file pgen.1007371.s007.zip › locuszoomplot/LOCUSZOOM_locusnbr-19_17-7754993_rs12939056.pdf]

# Candidate locus # 20

## HapMap and imputation results

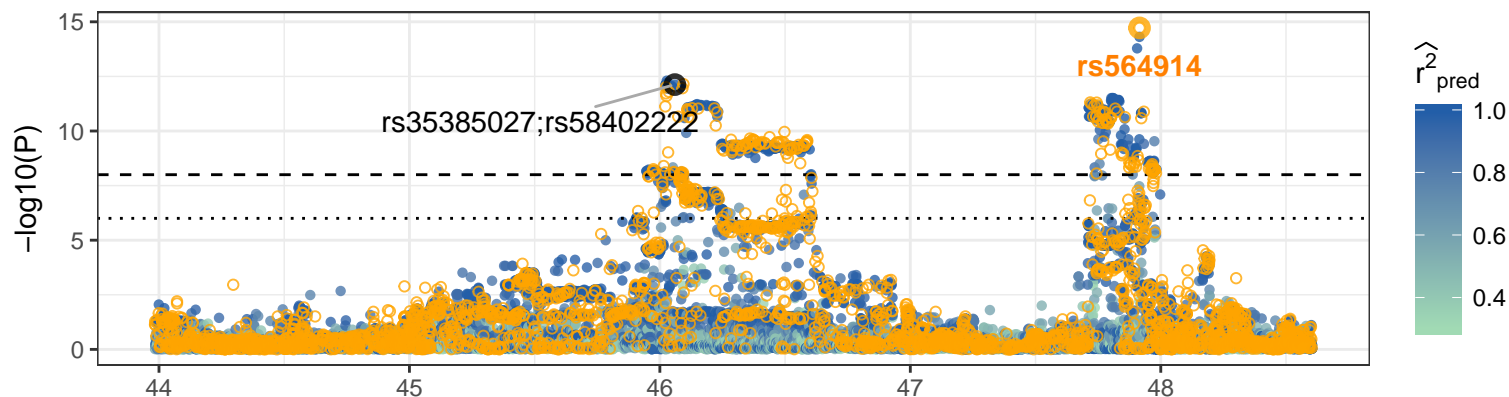

## Genes

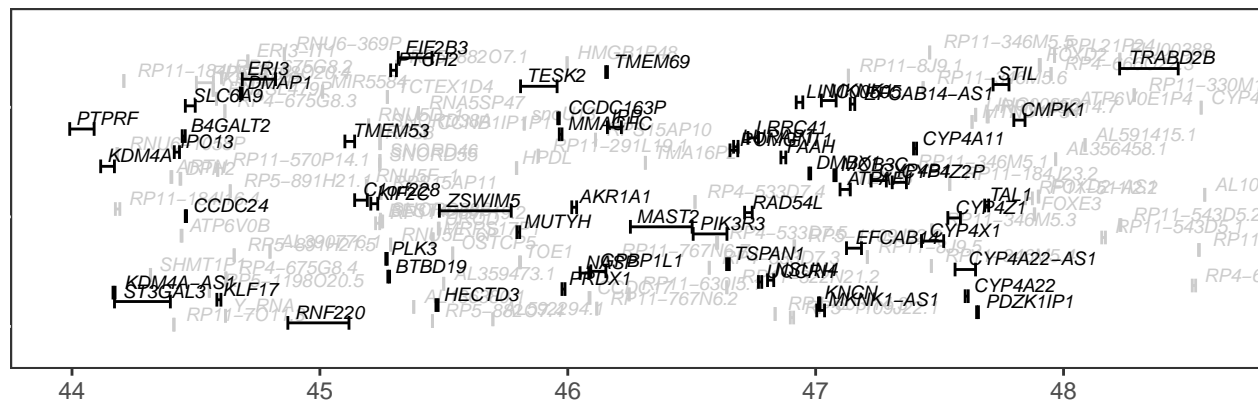

## Exome results

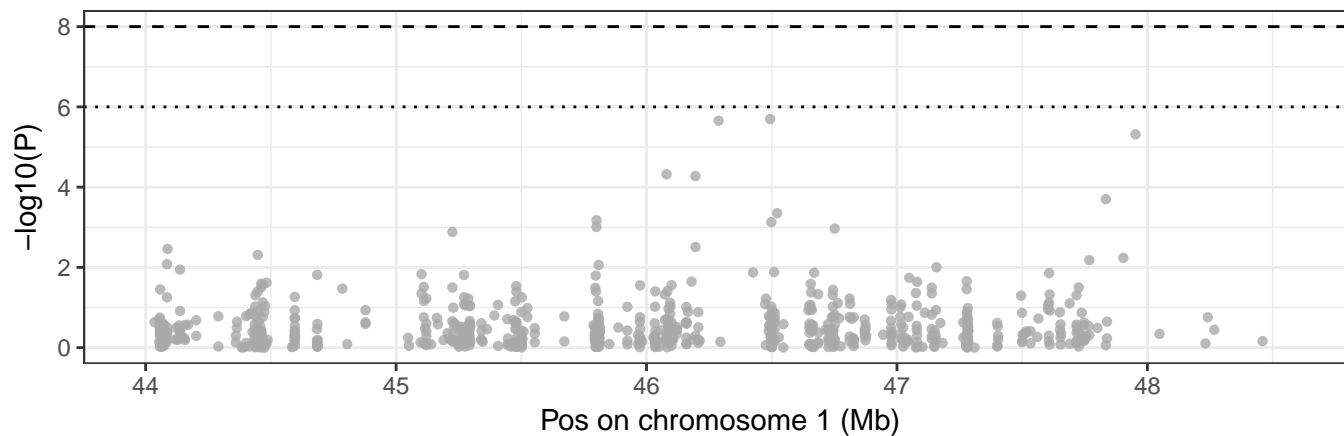

Supplement: S7 Fig — This figure shows three datasets: Results from the HapMap and the exome chip study, and imputed summary statistics. The top window shows HapMap P-values as orange circles and the imputed P-values (using summary statistics imputation) as solid circles, with the colour representing the imputation quality (only r^pred,adj2≥0.3 shown). The bottom window shows exome chip study results as solid, grey dots. Each dot represents the summary statistics of one variant. The x-axis shows the position (in Mb) on a ≥ 2 Mb range and the y-axis the −log10(P)-value. The horizontal line shows the P-value threshold of 10−6 (dotted) and 10−8 (dashed). Top and bottom window have annotated summary statistics: In the bottom window we mark dots as black if it is are part of the 122 reported hits of [13]. In the top window we mark the rs-id of variants that are part of the 122 reported variants of [13] in bold black, and if they are part of the 697 variants of [12] in bold orange font. Variants that are black (plain) are imputed variants (that had the lowest conditional P-value). Variants in orange (plain) are HapMap variants, but were not among the 697 reported hits. Each of the annotated variants is marked for clarity with a bold circle in the respective colour. The genes annotated in the middle window are printed in grey if the gene has a length < 5′000 bp or is an unrecognised gene (RP-). (ZIP) [file pgen.1007371.s007.zip › locuszoomplot/LOCUSZOOM_locusnbr-20_1-46059835_rs35385027;rs58402222.pdf]

# Candidate locus # 27

## HapMap and imputation results

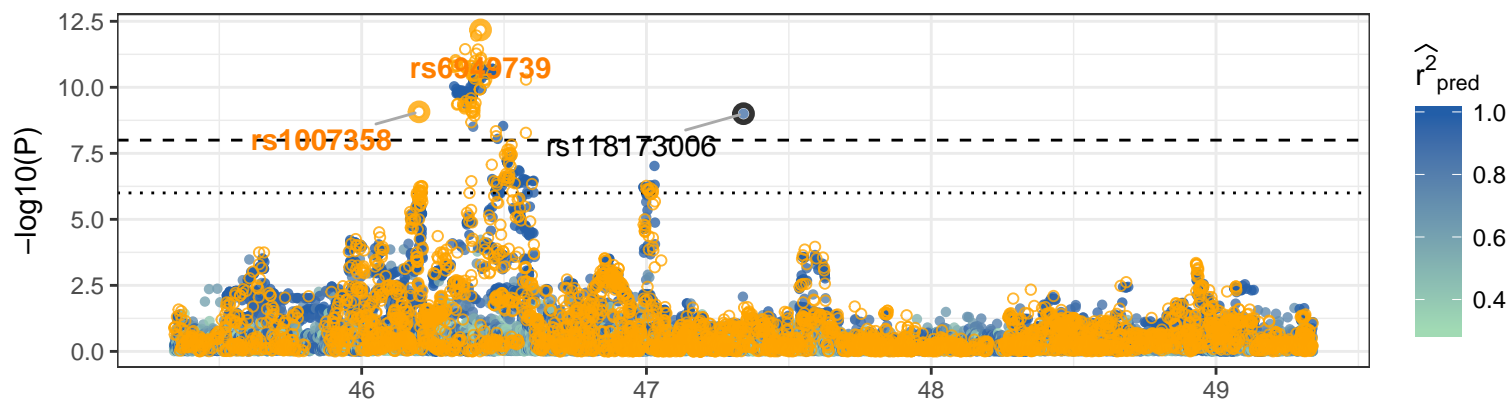

## Genes

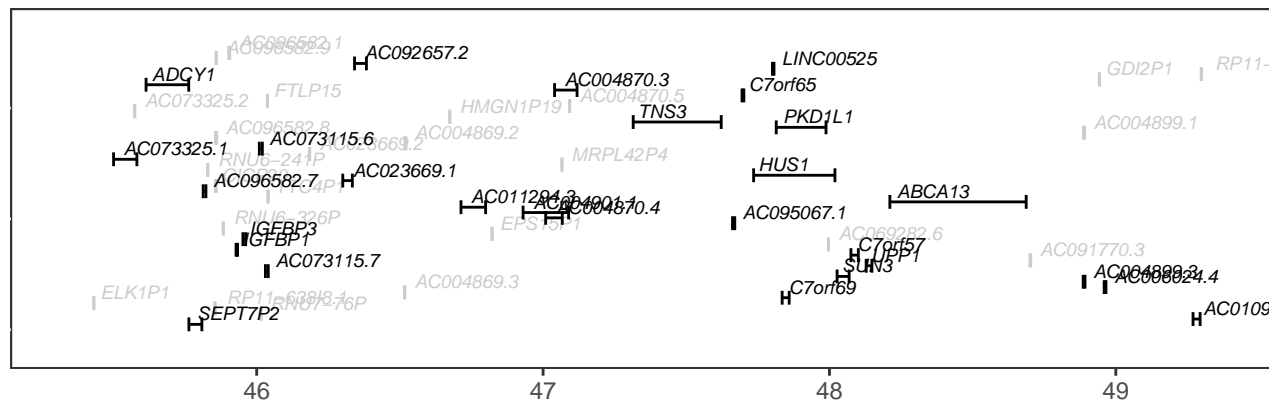

## Exome results

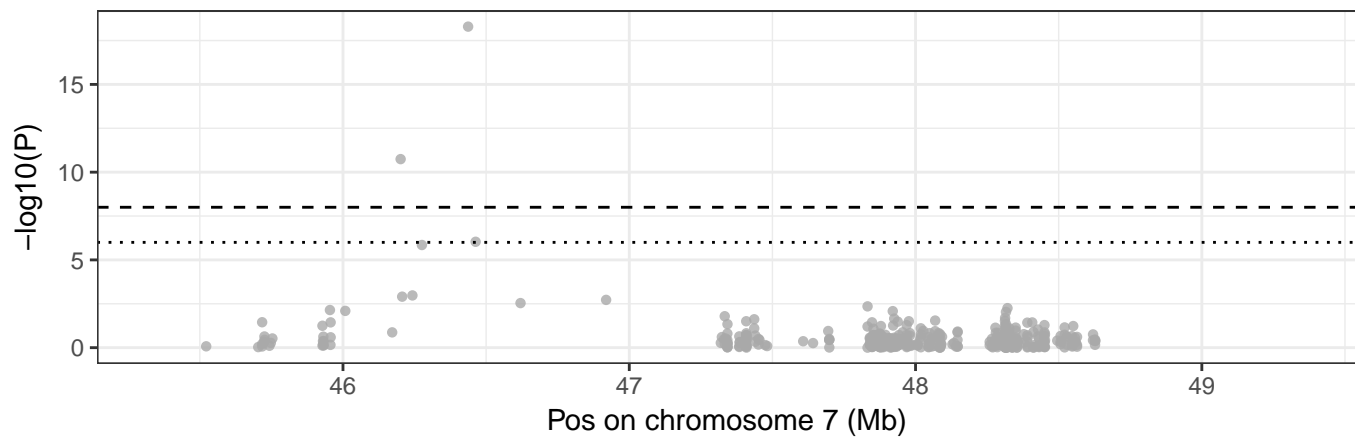

Supplement: S7 Fig — This figure shows three datasets: Results from the HapMap and the exome chip study, and imputed summary statistics. The top window shows HapMap P-values as orange circles and the imputed P-values (using summary statistics imputation) as solid circles, with the colour representing the imputation quality (only r^pred,adj2≥0.3 shown). The bottom window shows exome chip study results as solid, grey dots. Each dot represents the summary statistics of one variant. The x-axis shows the position (in Mb) on a ≥ 2 Mb range and the y-axis the −log10(P)-value. The horizontal line shows the P-value threshold of 10−6 (dotted) and 10−8 (dashed). Top and bottom window have annotated summary statistics: In the bottom window we mark dots as black if it is are part of the 122 reported hits of [13]. In the top window we mark the rs-id of variants that are part of the 122 reported variants of [13] in bold black, and if they are part of the 697 variants of [12] in bold orange font. Variants that are black (plain) are imputed variants (that had the lowest conditional P-value). Variants in orange (plain) are HapMap variants, but were not among the 697 reported hits. Each of the annotated variants is marked for clarity with a bold circle in the respective colour. The genes annotated in the middle window are printed in grey if the gene has a length < 5′000 bp or is an unrecognised gene (RP-). (ZIP) [file pgen.1007371.s007.zip › locuszoomplot/LOCUSZOOM_locusnbr-27_7-47340793_rs118173006.pdf]

# Candidate locus # 4

## HapMap and imputation results

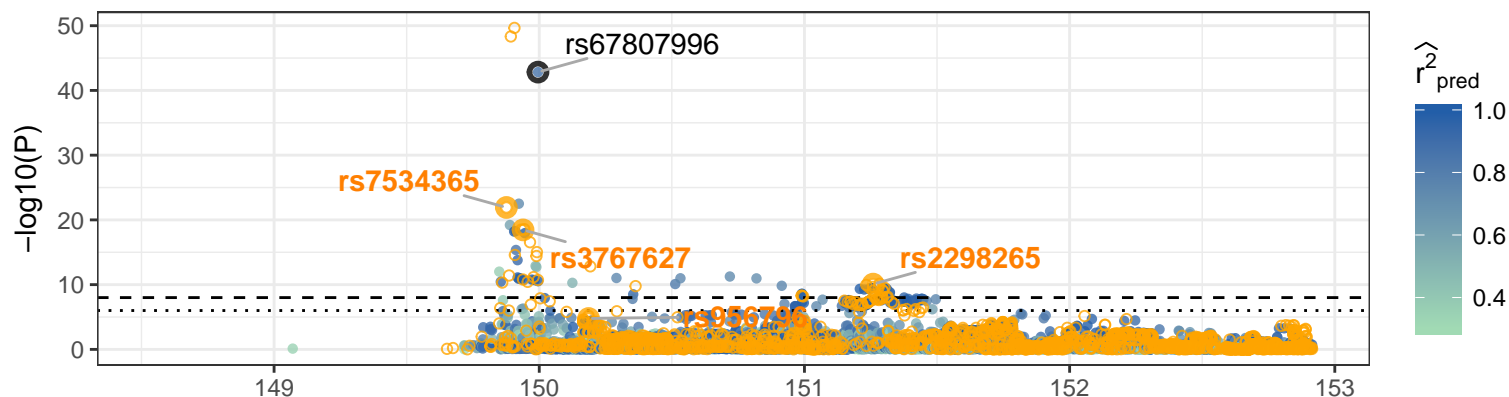

## Genes

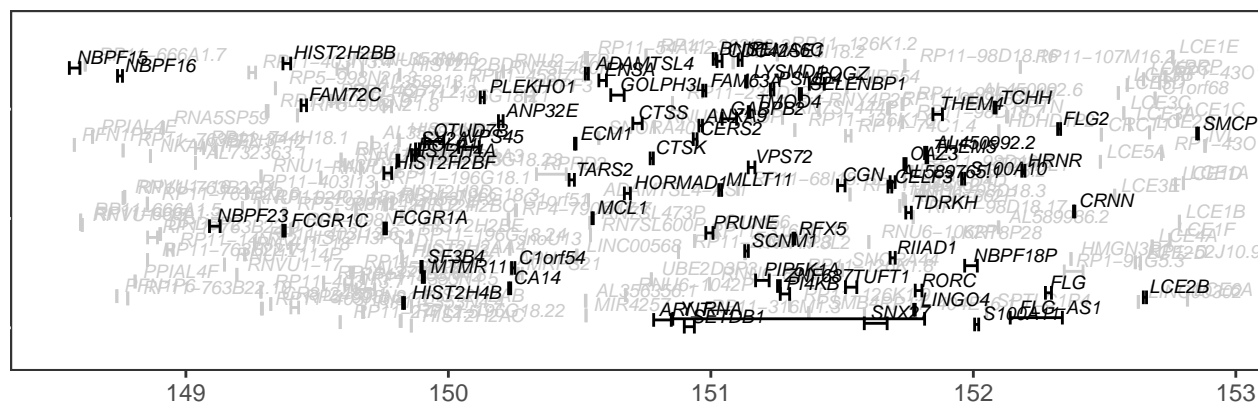

## Exome results

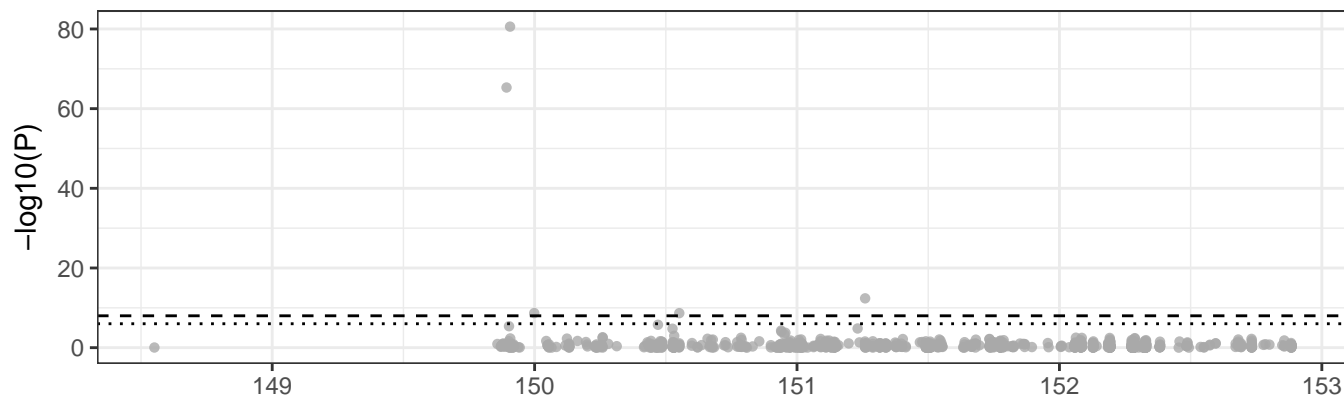

Supplement: S7 Fig — This figure shows three datasets: Results from the HapMap and the exome chip study, and imputed summary statistics. The top window shows HapMap P-values as orange circles and the imputed P-values (using summary statistics imputation) as solid circles, with the colour representing the imputation quality (only r^pred,adj2≥0.3 shown). The bottom window shows exome chip study results as solid, grey dots. Each dot represents the summary statistics of one variant. The x-axis shows the position (in Mb) on a ≥ 2 Mb range and the y-axis the −log10(P)-value. The horizontal line shows the P-value threshold of 10−6 (dotted) and 10−8 (dashed). Top and bottom window have annotated summary statistics: In the bottom window we mark dots as black if it is are part of the 122 reported hits of [13]. In the top window we mark the rs-id of variants that are part of the 122 reported variants of [13] in bold black, and if they are part of the 697 variants of [12] in bold orange font. Variants that are black (plain) are imputed variants (that had the lowest conditional P-value). Variants in orange (plain) are HapMap variants, but were not among the 697 reported hits. Each of the annotated variants is marked for clarity with a bold circle in the respective colour. The genes annotated in the middle window are printed in grey if the gene has a length < 5′000 bp or is an unrecognised gene (RP-). (ZIP) [file pgen.1007371.s007.zip › locuszoomplot/LOCUSZOOM_locusnbr-4_1-149995265_rs67807996.pdf]

## Candidate locus # 17

## HapMap and imputation results

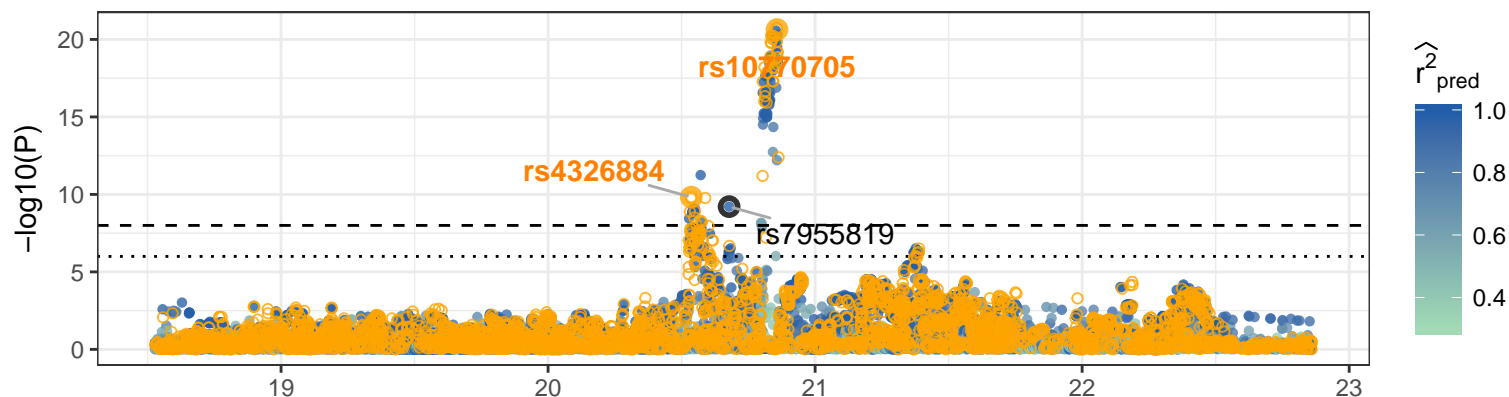

## Genes

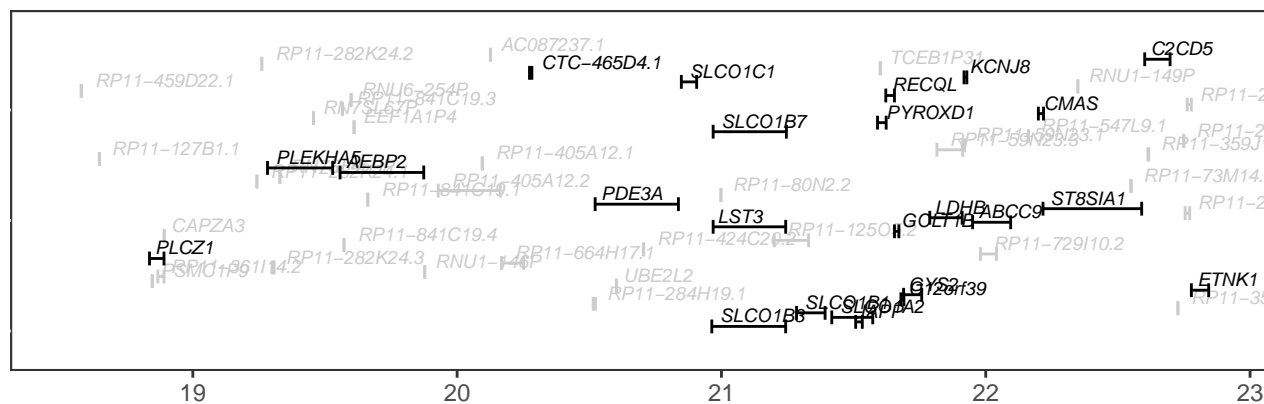

## Exome results

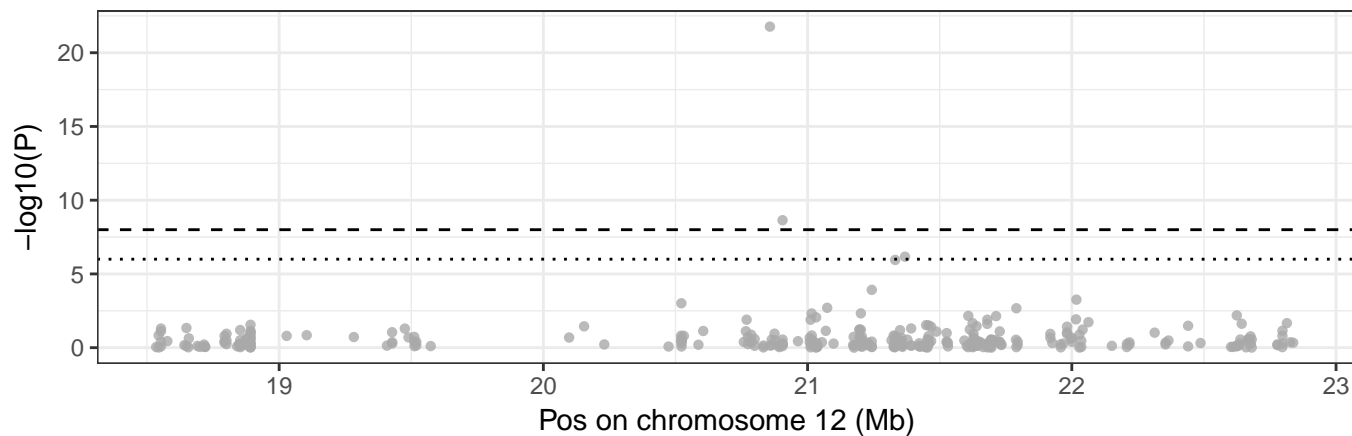

Supplement: S7 Fig — This figure shows three datasets: Results from the HapMap and the exome chip study, and imputed summary statistics. The top window shows HapMap P-values as orange circles and the imputed P-values (using summary statistics imputation) as solid circles, with the colour representing the imputation quality (only r^pred,adj2≥0.3 shown). The bottom window shows exome chip study results as solid, grey dots. Each dot represents the summary statistics of one variant. The x-axis shows the position (in Mb) on a ≥ 2 Mb range and the y-axis the −log10(P)-value. The horizontal line shows the P-value threshold of 10−6 (dotted) and 10−8 (dashed). Top and bottom window have annotated summary statistics: In the bottom window we mark dots as black if it is are part of the 122 reported hits of [13]. In the top window we mark the rs-id of variants that are part of the 122 reported variants of [13] in bold black, and if they are part of the 697 variants of [12] in bold orange font. Variants that are black (plain) are imputed variants (that had the lowest conditional P-value). Variants in orange (plain) are HapMap variants, but were not among the 697 reported hits. Each of the annotated variants is marked for clarity with a bold circle in the respective colour. The genes annotated in the middle window are printed in grey if the gene has a length < 5′000 bp or is an unrecognised gene (RP-). (ZIP) [file pgen.1007371.s007.zip › locuszoomplot/LOCUSZOOM_locusnbr-17_12-20677958_rs7955819.pdf]

## Candidate locus # 31

## HapMap and imputation results

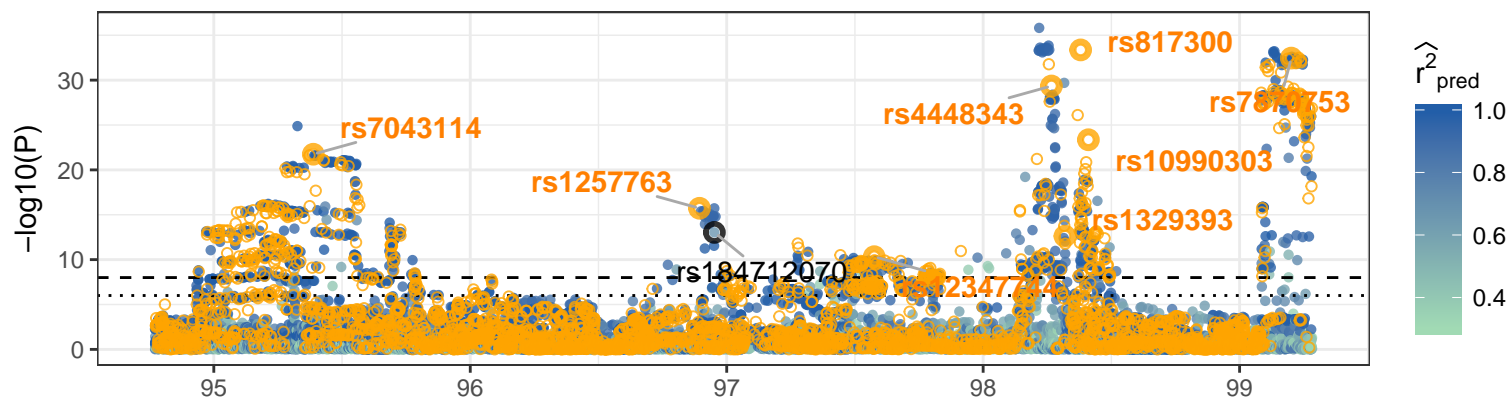

## Genes

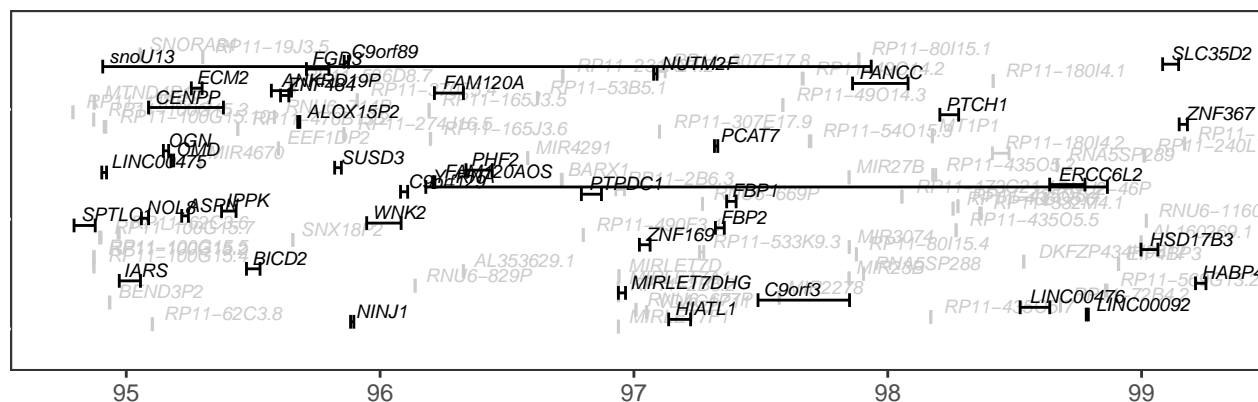

## Exome results

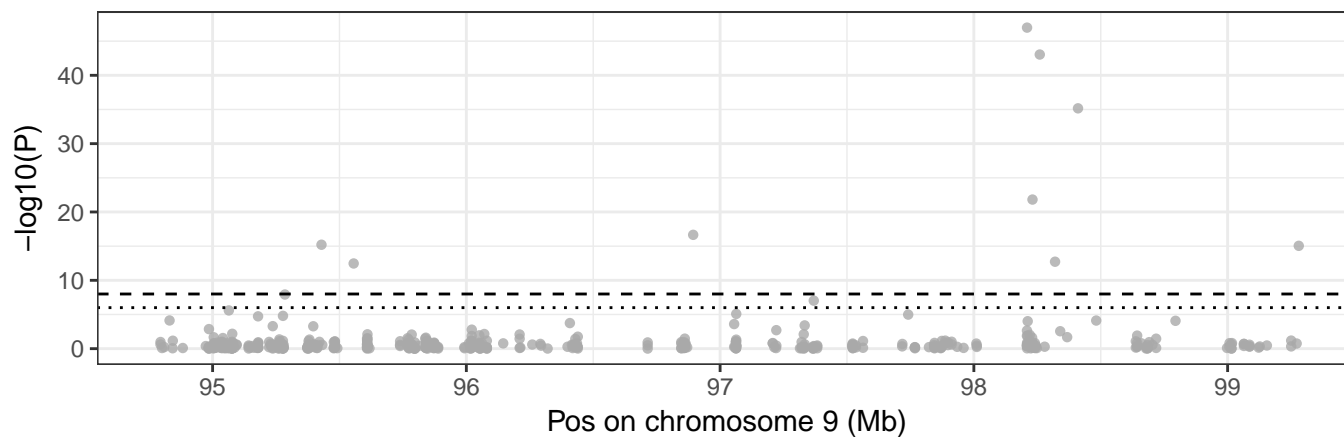

Supplement: S7 Fig — This figure shows three datasets: Results from the HapMap and the exome chip study, and imputed summary statistics. The top window shows HapMap P-values as orange circles and the imputed P-values (using summary statistics imputation) as solid circles, with the colour representing the imputation quality (only r^pred,adj2≥0.3 shown). The bottom window shows exome chip study results as solid, grey dots. Each dot represents the summary statistics of one variant. The x-axis shows the position (in Mb) on a ≥ 2 Mb range and the y-axis the −log10(P)-value. The horizontal line shows the P-value threshold of 10−6 (dotted) and 10−8 (dashed). Top and bottom window have annotated summary statistics: In the bottom window we mark dots as black if it is are part of the 122 reported hits of [13]. In the top window we mark the rs-id of variants that are part of the 122 reported variants of [13] in bold black, and if they are part of the 697 variants of [12] in bold orange font. Variants that are black (plain) are imputed variants (that had the lowest conditional P-value). Variants in orange (plain) are HapMap variants, but were not among the 697 reported hits. Each of the annotated variants is marked for clarity with a bold circle in the respective colour. The genes annotated in the middle window are printed in grey if the gene has a length < 5′000 bp or is an unrecognised gene (RP-). (ZIP) [file pgen.1007371.s007.zip › locuszoomplot/LOCUSZOOM_locusnbr-31_9-96951880_rs184712070.pdf]

## HapMap and imputation results

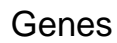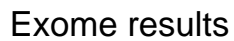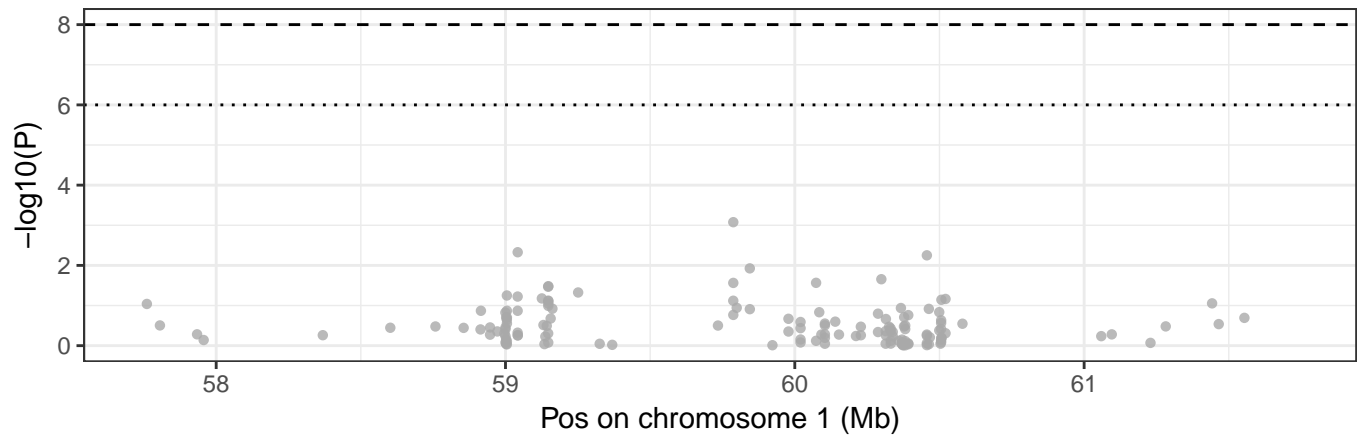

Supplement: S7 Fig — This figure shows three datasets: Results from the HapMap and the exome chip study, and imputed summary statistics. The top window shows HapMap P-values as orange circles and the imputed P-values (using summary statistics imputation) as solid circles, with the colour representing the imputation quality (only r^pred,adj2≥0.3 shown). The bottom window shows exome chip study results as solid, grey dots. Each dot represents the summary statistics of one variant. The x-axis shows the position (in Mb) on a ≥ 2 Mb range and the y-axis the −log10(P)-value. The horizontal line shows the P-value threshold of 10−6 (dotted) and 10−8 (dashed). Top and bottom window have annotated summary statistics: In the bottom window we mark dots as black if it is are part of the 122 reported hits of [13]. In the top window we mark the rs-id of variants that are part of the 122 reported variants of [13] in bold black, and if they are part of the 697 variants of [12] in bold orange font. Variants that are black (plain) are imputed variants (that had the lowest conditional P-value). Variants in orange (plain) are HapMap variants, but were not among the 697 reported hits. Each of the annotated variants is marked for clarity with a bold circle in the respective colour. The genes annotated in the middle window are printed in grey if the gene has a length < 5′000 bp or is an unrecognised gene (RP-). (ZIP) [file pgen.1007371.s007.zip › locuszoomplot/LOCUSZOOM_locusnbr-21_1-59742379_rs187957016.pdf]

# Candidate locus # 35

## HapMap and imputation results

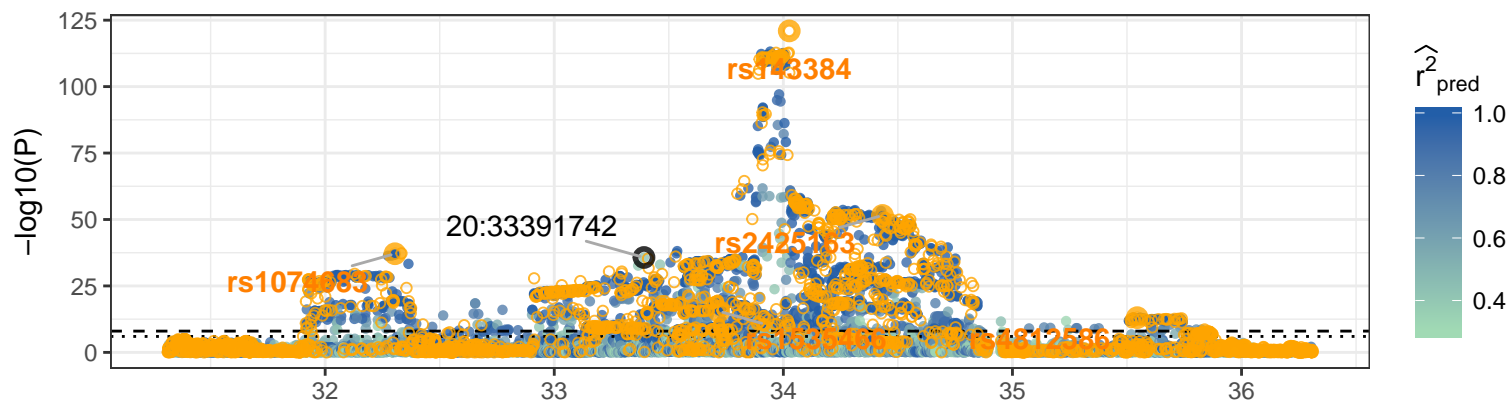

## Genes

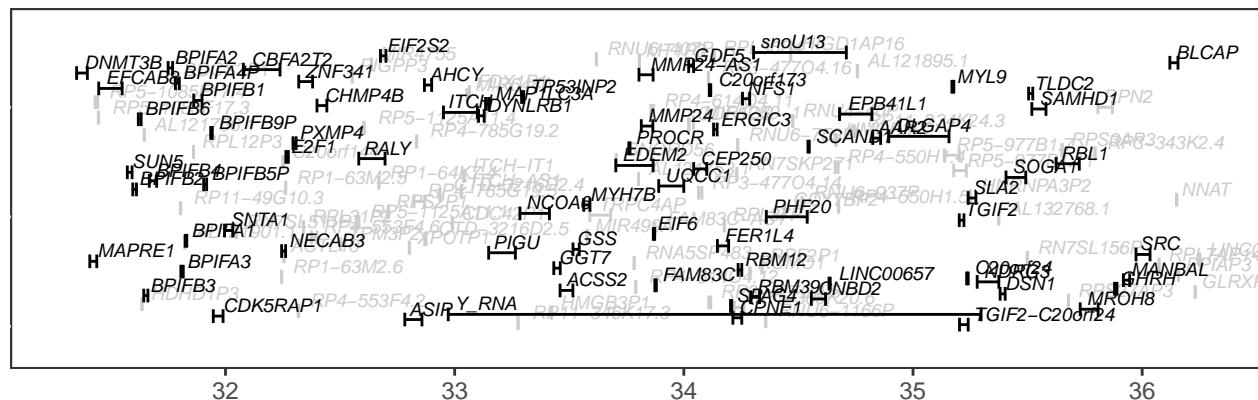

## Exome results

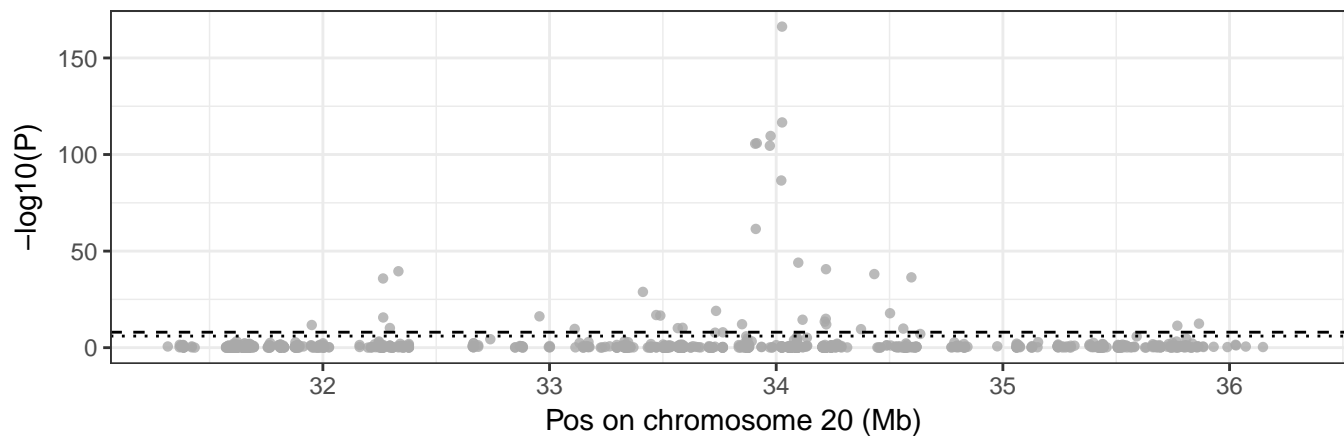

Supplement: S7 Fig — This figure shows three datasets: Results from the HapMap and the exome chip study, and imputed summary statistics. The top window shows HapMap P-values as orange circles and the imputed P-values (using summary statistics imputation) as solid circles, with the colour representing the imputation quality (only r^pred,adj2≥0.3 shown). The bottom window shows exome chip study results as solid, grey dots. Each dot represents the summary statistics of one variant. The x-axis shows the position (in Mb) on a ≥ 2 Mb range and the y-axis the −log10(P)-value. The horizontal line shows the P-value threshold of 10−6 (dotted) and 10−8 (dashed). Top and bottom window have annotated summary statistics: In the bottom window we mark dots as black if it is are part of the 122 reported hits of [13]. In the top window we mark the rs-id of variants that are part of the 122 reported variants of [13] in bold black, and if they are part of the 697 variants of [12] in bold orange font. Variants that are black (plain) are imputed variants (that had the lowest conditional P-value). Variants in orange (plain) are HapMap variants, but were not among the 697 reported hits. Each of the annotated variants is marked for clarity with a bold circle in the respective colour. The genes annotated in the middle window are printed in grey if the gene has a length < 5′000 bp or is an unrecognised gene (RP-). (ZIP) [file pgen.1007371.s007.zip › locuszoomplot/LOCUSZOOM_locusnbr-35_20-33391742_20:33391742.pdf]

# Candidate locus # 5

## HapMap and imputation results

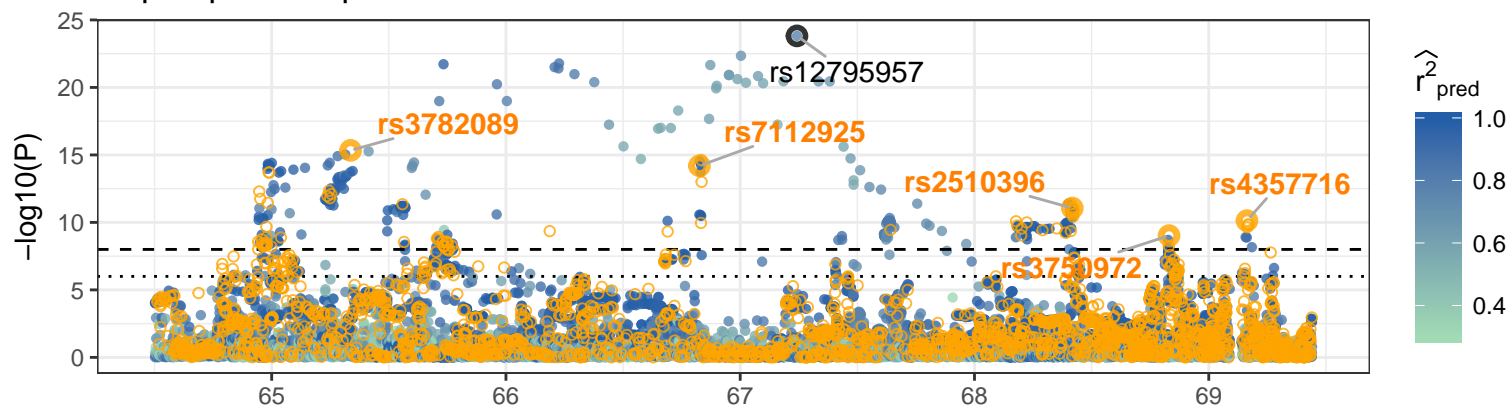

## Genes

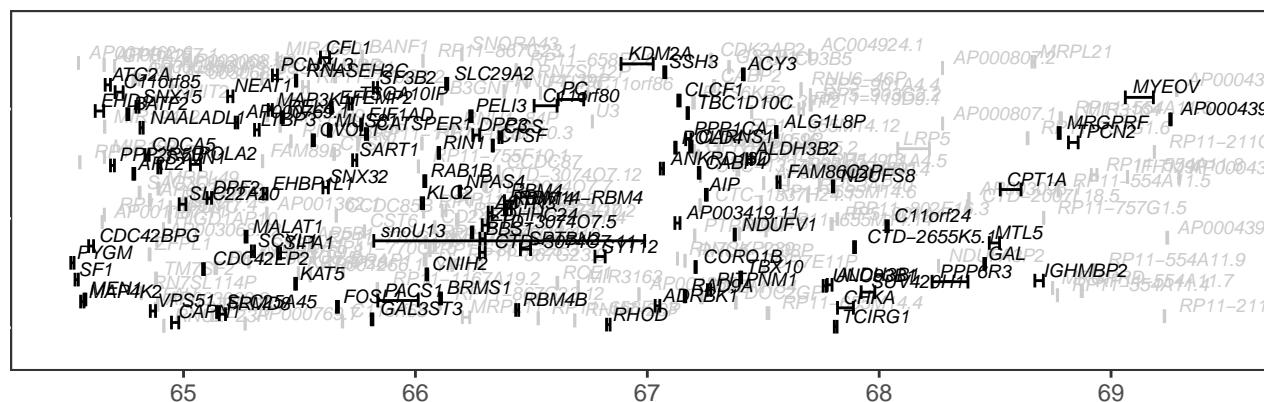

## Exome results

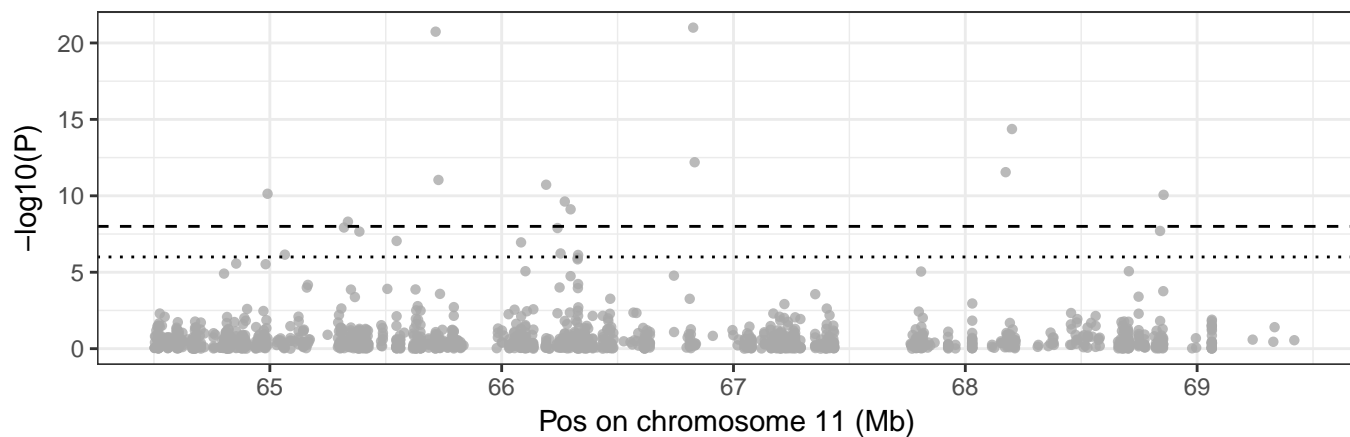

Supplement: S7 Fig — This figure shows three datasets: Results from the HapMap and the exome chip study, and imputed summary statistics. The top window shows HapMap P-values as orange circles and the imputed P-values (using summary statistics imputation) as solid circles, with the colour representing the imputation quality (only r^pred,adj2≥0.3 shown). The bottom window shows exome chip study results as solid, grey dots. Each dot represents the summary statistics of one variant. The x-axis shows the position (in Mb) on a ≥ 2 Mb range and the y-axis the −log10(P)-value. The horizontal line shows the P-value threshold of 10−6 (dotted) and 10−8 (dashed). Top and bottom window have annotated summary statistics: In the bottom window we mark dots as black if it is are part of the 122 reported hits of [13]. In the top window we mark the rs-id of variants that are part of the 122 reported variants of [13] in bold black, and if they are part of the 697 variants of [12] in bold orange font. Variants that are black (plain) are imputed variants (that had the lowest conditional P-value). Variants in orange (plain) are HapMap variants, but were not among the 697 reported hits. Each of the annotated variants is marked for clarity with a bold circle in the respective colour. The genes annotated in the middle window are printed in grey if the gene has a length < 5′000 bp or is an unrecognised gene (RP-). (ZIP) [file pgen.1007371.s007.zip › locuszoomplot/LOCUSZOOM_locusnbr-5_11-67242216_rs12795957.pdf]

## HapMap and imputation results

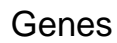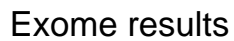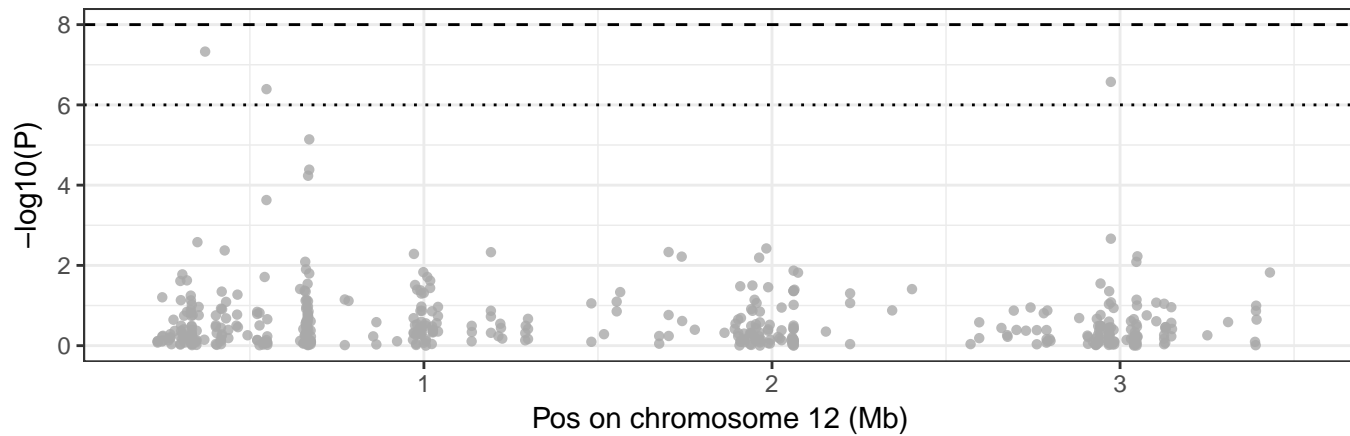

Supplement: S7 Fig — This figure shows three datasets: Results from the HapMap and the exome chip study, and imputed summary statistics. The top window shows HapMap P-values as orange circles and the imputed P-values (using summary statistics imputation) as solid circles, with the colour representing the imputation quality (only r^pred,adj2≥0.3 shown). The bottom window shows exome chip study results as solid, grey dots. Each dot represents the summary statistics of one variant. The x-axis shows the position (in Mb) on a ≥ 2 Mb range and the y-axis the −log10(P)-value. The horizontal line shows the P-value threshold of 10−6 (dotted) and 10−8 (dashed). Top and bottom window have annotated summary statistics: In the bottom window we mark dots as black if it is are part of the 122 reported hits of [13]. In the top window we mark the rs-id of variants that are part of the 122 reported variants of [13] in bold black, and if they are part of the 697 variants of [12] in bold orange font. Variants that are black (plain) are imputed variants (that had the lowest conditional P-value). Variants in orange (plain) are HapMap variants, but were not among the 697 reported hits. Each of the annotated variants is marked for clarity with a bold circle in the respective colour. The genes annotated in the middle window are printed in grey if the gene has a length < 5′000 bp or is an unrecognised gene (RP-). (ZIP) [file pgen.1007371.s007.zip › locuszoomplot/LOCUSZOOM_locusnbr-18_12-1513526_rs7971674.pdf]

## Candidate locus # 11

## HapMap and imputation results

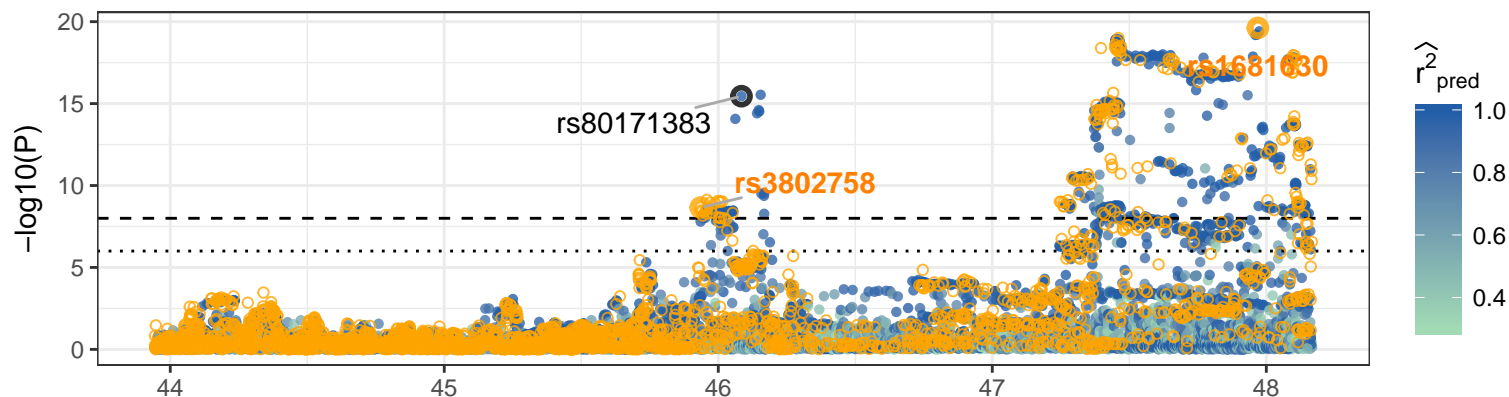

## Genes

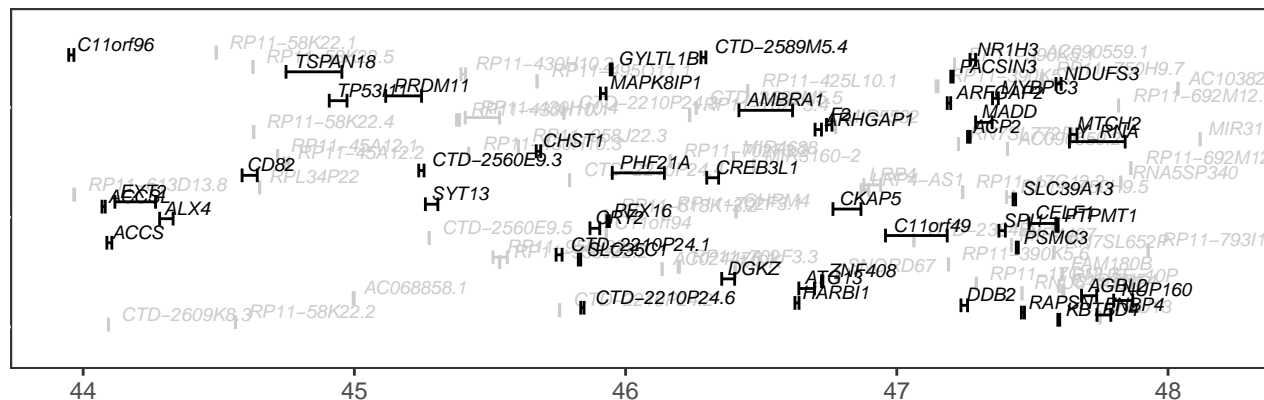

## Exome results

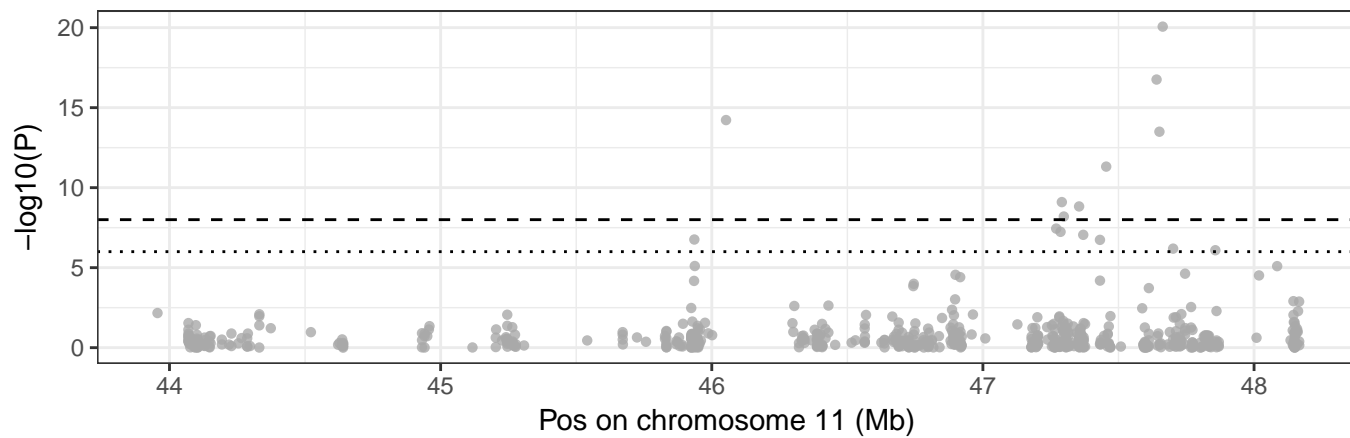

Supplement: S7 Fig — This figure shows three datasets: Results from the HapMap and the exome chip study, and imputed summary statistics. The top window shows HapMap P-values as orange circles and the imputed P-values (using summary statistics imputation) as solid circles, with the colour representing the imputation quality (only r^pred,adj2≥0.3 shown). The bottom window shows exome chip study results as solid, grey dots. Each dot represents the summary statistics of one variant. The x-axis shows the position (in Mb) on a ≥ 2 Mb range and the y-axis the −log10(P)-value. The horizontal line shows the P-value threshold of 10−6 (dotted) and 10−8 (dashed). Top and bottom window have annotated summary statistics: In the bottom window we mark dots as black if it is are part of the 122 reported hits of [13]. In the top window we mark the rs-id of variants that are part of the 122 reported variants of [13] in bold black, and if they are part of the 697 variants of [12] in bold orange font. Variants that are black (plain) are imputed variants (that had the lowest conditional P-value). Variants in orange (plain) are HapMap variants, but were not among the 697 reported hits. Each of the annotated variants is marked for clarity with a bold circle in the respective colour. The genes annotated in the middle window are printed in grey if the gene has a length < 5′000 bp or is an unrecognised gene (RP-). (ZIP) [file pgen.1007371.s007.zip › locuszoomplot/LOCUSZOOM_locusnbr-11_11-46084677_rs80171383.pdf]

## Candidate locus # 24

## HapMap and imputation results

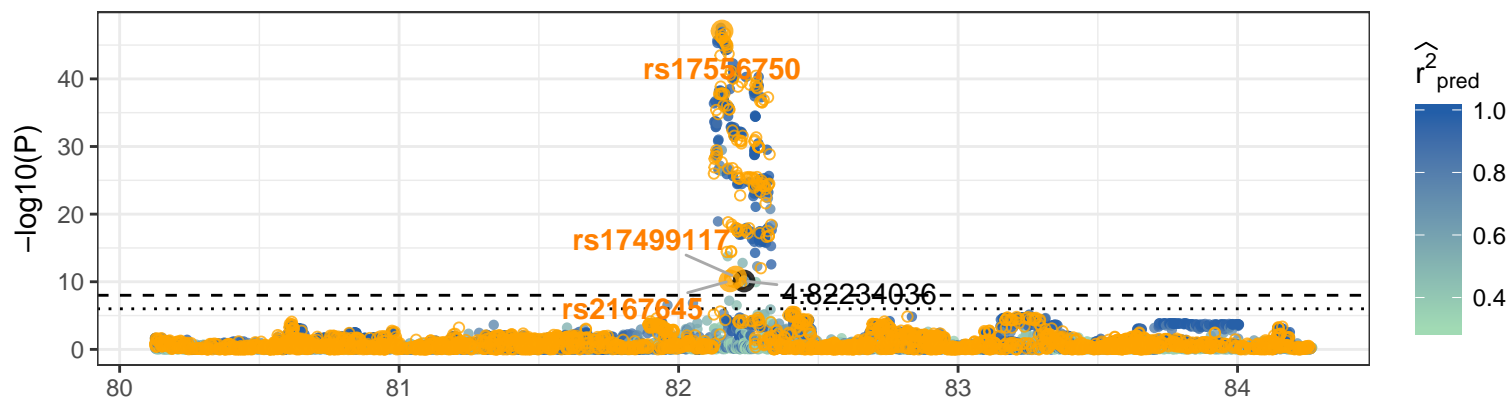

## Genes

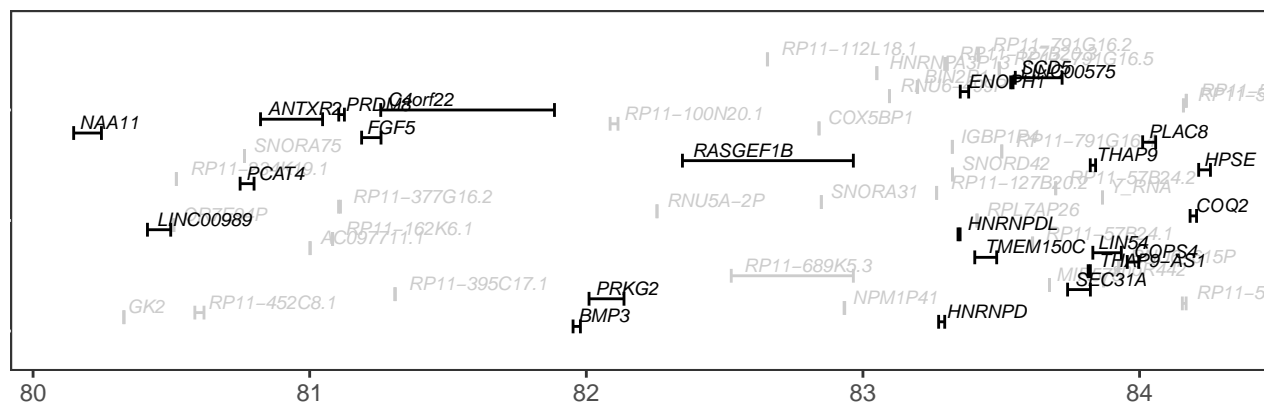

## Exome results

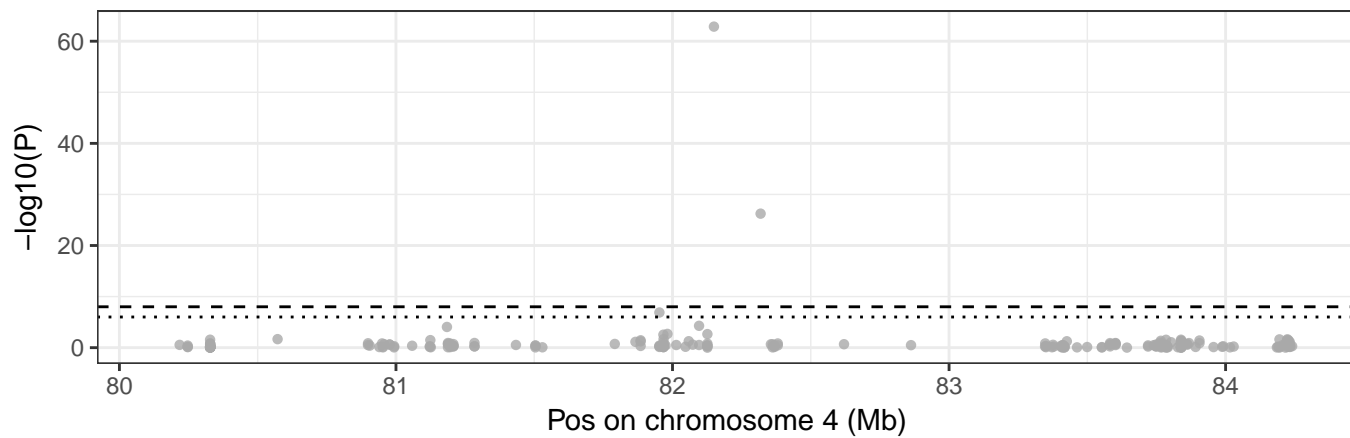

Supplement: S7 Fig — This figure shows three datasets: Results from the HapMap and the exome chip study, and imputed summary statistics. The top window shows HapMap P-values as orange circles and the imputed P-values (using summary statistics imputation) as solid circles, with the colour representing the imputation quality (only r^pred,adj2≥0.3 shown). The bottom window shows exome chip study results as solid, grey dots. Each dot represents the summary statistics of one variant. The x-axis shows the position (in Mb) on a ≥ 2 Mb range and the y-axis the −log10(P)-value. The horizontal line shows the P-value threshold of 10−6 (dotted) and 10−8 (dashed). Top and bottom window have annotated summary statistics: In the bottom window we mark dots as black if it is are part of the 122 reported hits of [13]. In the top window we mark the rs-id of variants that are part of the 122 reported variants of [13] in bold black, and if they are part of the 697 variants of [12] in bold orange font. Variants that are black (plain) are imputed variants (that had the lowest conditional P-value). Variants in orange (plain) are HapMap variants, but were not among the 697 reported hits. Each of the annotated variants is marked for clarity with a bold circle in the respective colour. The genes annotated in the middle window are printed in grey if the gene has a length < 5′000 bp or is an unrecognised gene (RP-). (ZIP) [file pgen.1007371.s007.zip › locuszoomplot/LOCUSZOOM_locusnbr-24_4-82234036_4:82234036.pdf]

# Candidate locus # 23

## HapMap and imputation results

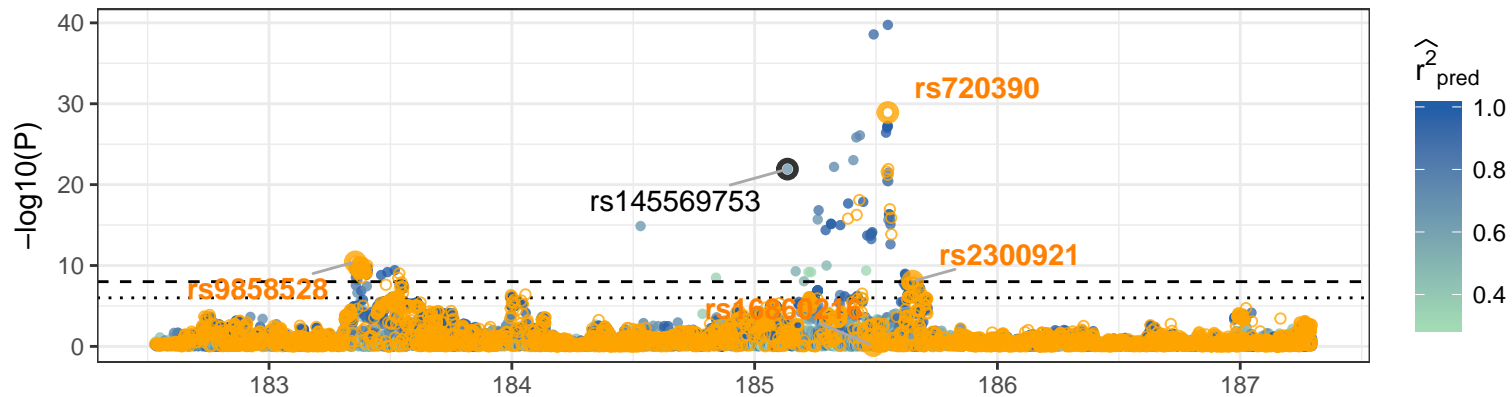

## Genes

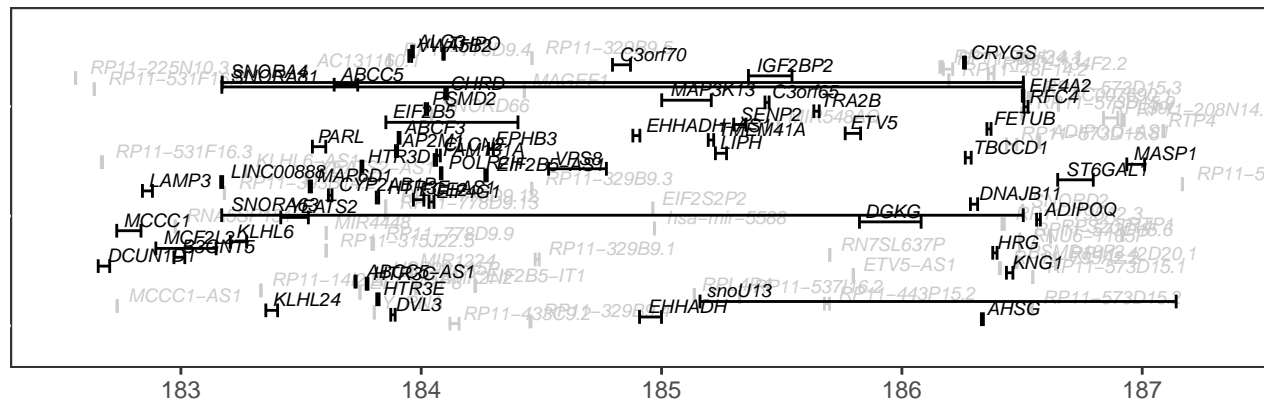

## Exome results

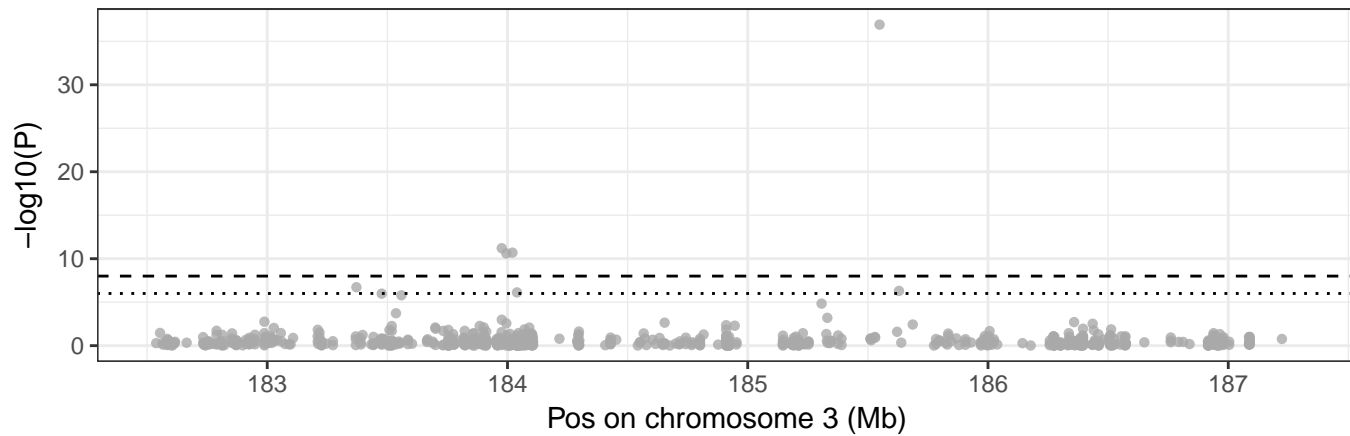

Supplement: S7 Fig — This figure shows three datasets: Results from the HapMap and the exome chip study, and imputed summary statistics. The top window shows HapMap P-values as orange circles and the imputed P-values (using summary statistics imputation) as solid circles, with the colour representing the imputation quality (only r^pred,adj2≥0.3 shown). The bottom window shows exome chip study results as solid, grey dots. Each dot represents the summary statistics of one variant. The x-axis shows the position (in Mb) on a ≥ 2 Mb range and the y-axis the −log10(P)-value. The horizontal line shows the P-value threshold of 10−6 (dotted) and 10−8 (dashed). Top and bottom window have annotated summary statistics: In the bottom window we mark dots as black if it is are part of the 122 reported hits of [13]. In the top window we mark the rs-id of variants that are part of the 122 reported variants of [13] in bold black, and if they are part of the 697 variants of [12] in bold orange font. Variants that are black (plain) are imputed variants (that had the lowest conditional P-value). Variants in orange (plain) are HapMap variants, but were not among the 697 reported hits. Each of the annotated variants is marked for clarity with a bold circle in the respective colour. The genes annotated in the middle window are printed in grey if the gene has a length < 5′000 bp or is an unrecognised gene (RP-). (ZIP) [file pgen.1007371.s007.zip › locuszoomplot/LOCUSZOOM_locusnbr-23_3-185135198_rs145569753.pdf]

# Candidate locus # 3

## HapMap and imputation results

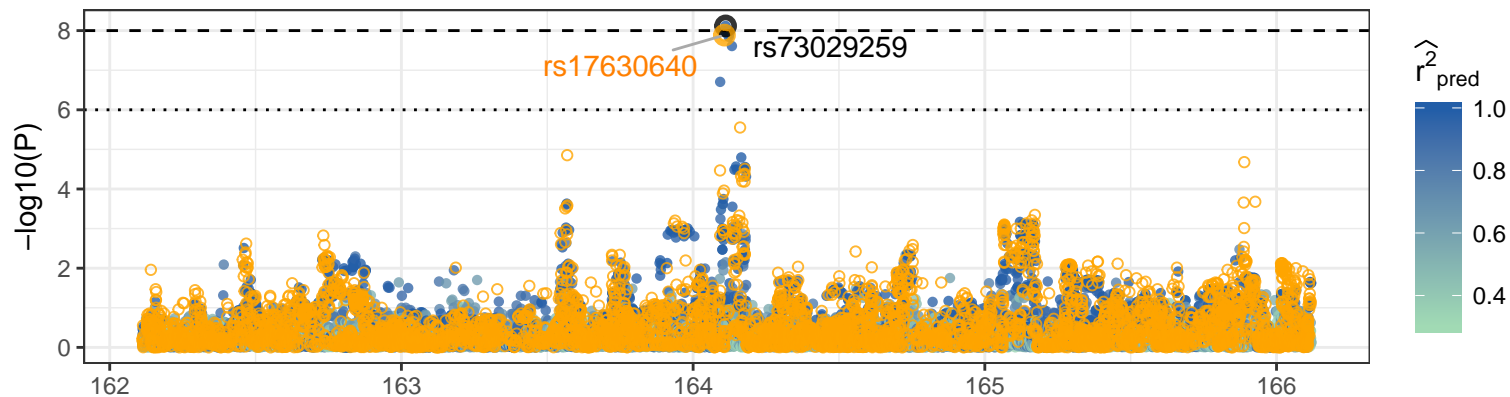

## Genes

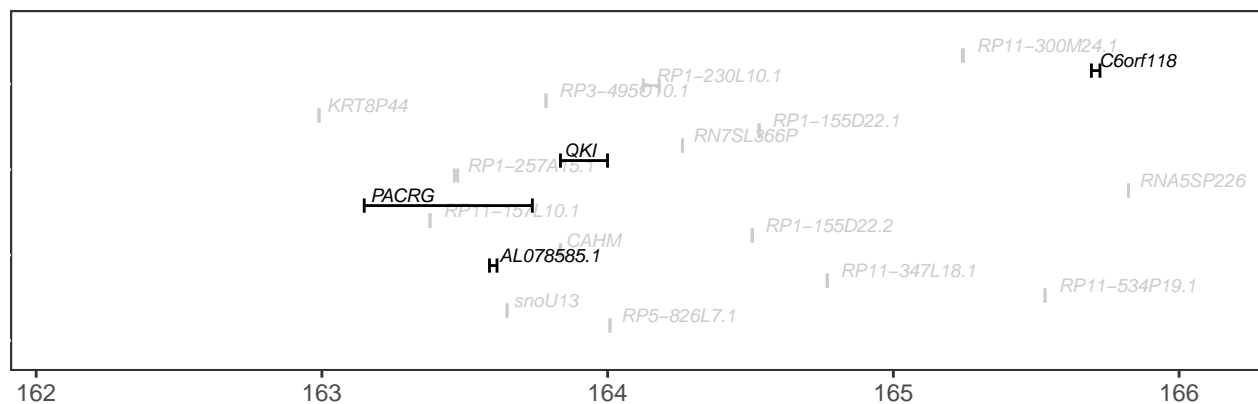

## Exome results

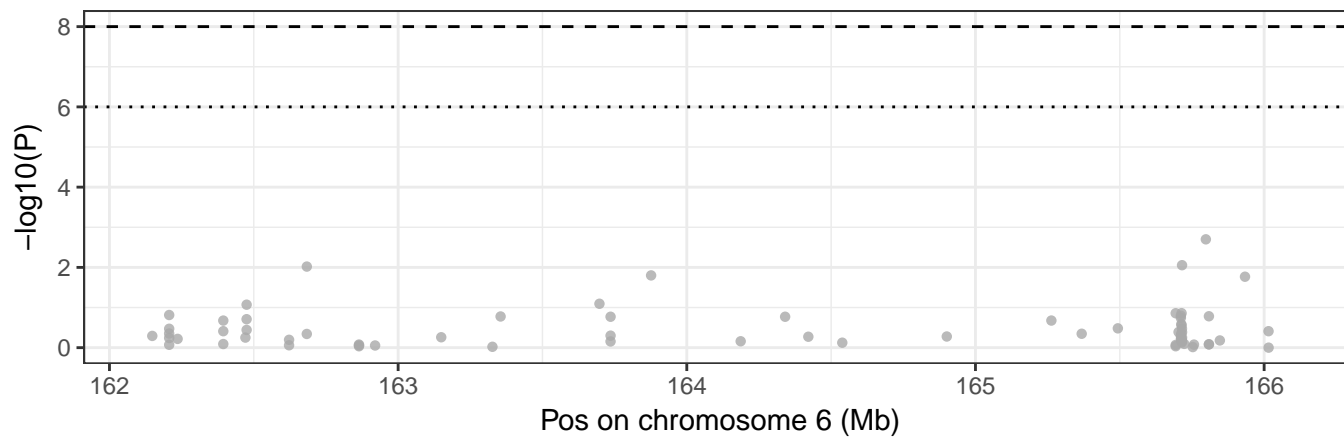

Supplement: S7 Fig — This figure shows three datasets: Results from the HapMap and the exome chip study, and imputed summary statistics. The top window shows HapMap P-values as orange circles and the imputed P-values (using summary statistics imputation) as solid circles, with the colour representing the imputation quality (only r^pred,adj2≥0.3 shown). The bottom window shows exome chip study results as solid, grey dots. Each dot represents the summary statistics of one variant. The x-axis shows the position (in Mb) on a ≥ 2 Mb range and the y-axis the −log10(P)-value. The horizontal line shows the P-value threshold of 10−6 (dotted) and 10−8 (dashed). Top and bottom window have annotated summary statistics: In the bottom window we mark dots as black if it is are part of the 122 reported hits of [13]. In the top window we mark the rs-id of variants that are part of the 122 reported variants of [13] in bold black, and if they are part of the 697 variants of [12] in bold orange font. Variants that are black (plain) are imputed variants (that had the lowest conditional P-value). Variants in orange (plain) are HapMap variants, but were not among the 697 reported hits. Each of the annotated variants is marked for clarity with a bold circle in the respective colour. The genes annotated in the middle window are printed in grey if the gene has a length < 5′000 bp or is an unrecognised gene (RP-). (ZIP) [file pgen.1007371.s007.zip › locuszoomplot/LOCUSZOOM_locusnbr-3_6-164111348_rs73029259.pdf]

# Candidate locus # 25

## HapMap and imputation results

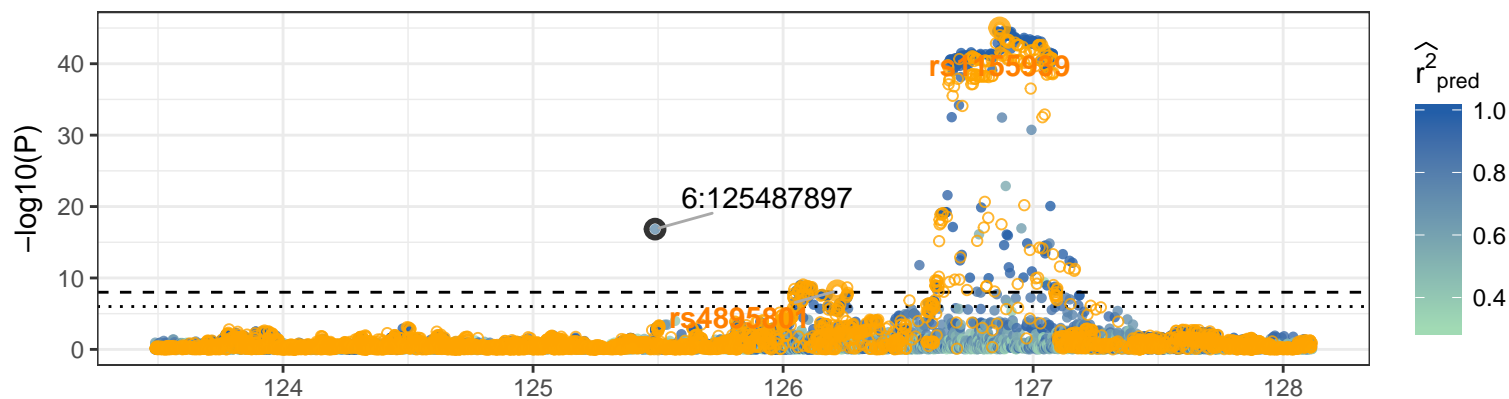

## Genes

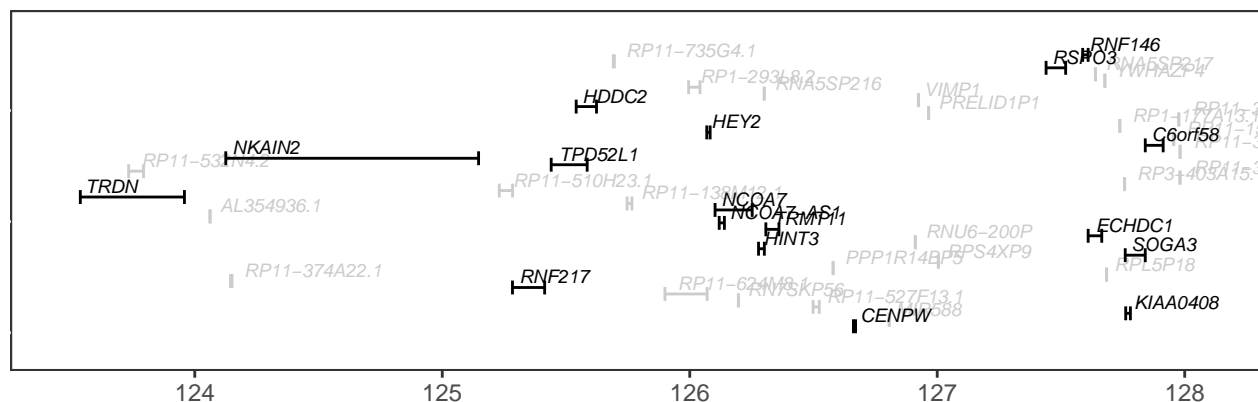

## Exome results

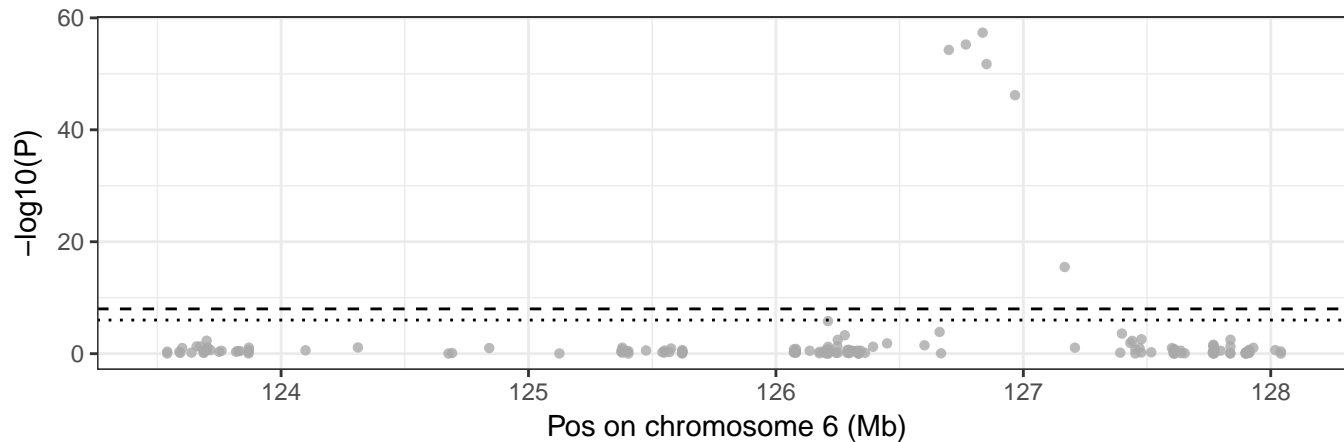

Supplement: S7 Fig — This figure shows three datasets: Results from the HapMap and the exome chip study, and imputed summary statistics. The top window shows HapMap P-values as orange circles and the imputed P-values (using summary statistics imputation) as solid circles, with the colour representing the imputation quality (only r^pred,adj2≥0.3 shown). The bottom window shows exome chip study results as solid, grey dots. Each dot represents the summary statistics of one variant. The x-axis shows the position (in Mb) on a ≥ 2 Mb range and the y-axis the −log10(P)-value. The horizontal line shows the P-value threshold of 10−6 (dotted) and 10−8 (dashed). Top and bottom window have annotated summary statistics: In the bottom window we mark dots as black if it is are part of the 122 reported hits of [13]. In the top window we mark the rs-id of variants that are part of the 122 reported variants of [13] in bold black, and if they are part of the 697 variants of [12] in bold orange font. Variants that are black (plain) are imputed variants (that had the lowest conditional P-value). Variants in orange (plain) are HapMap variants, but were not among the 697 reported hits. Each of the annotated variants is marked for clarity with a bold circle in the respective colour. The genes annotated in the middle window are printed in grey if the gene has a length < 5′000 bp or is an unrecognised gene (RP-). (ZIP) [file pgen.1007371.s007.zip › locuszoomplot/LOCUSZOOM_locusnbr-25_6-125487897_6:125487897.pdf]

## Candidate locus # 28

## HapMap and imputation results

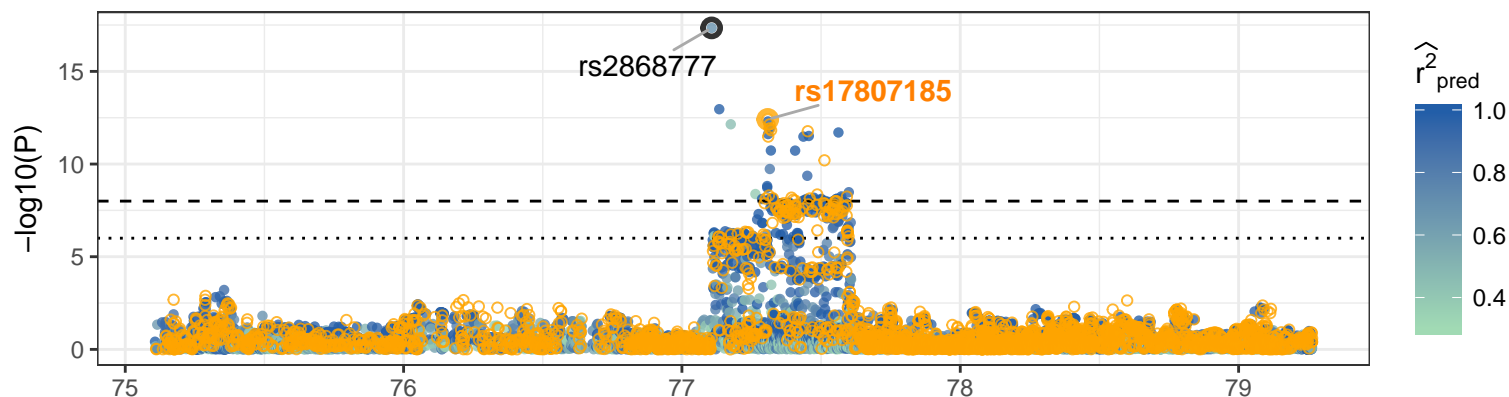

## Genes

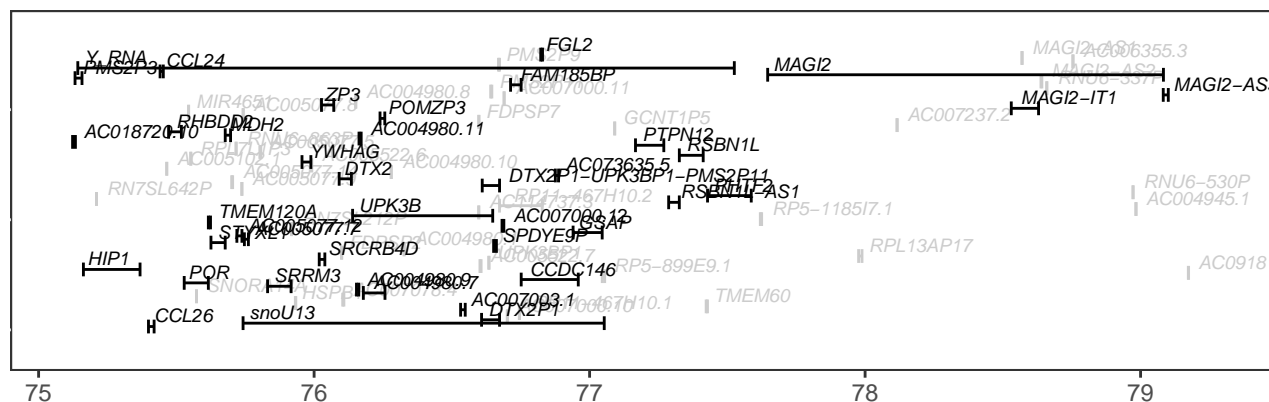

## Exome results

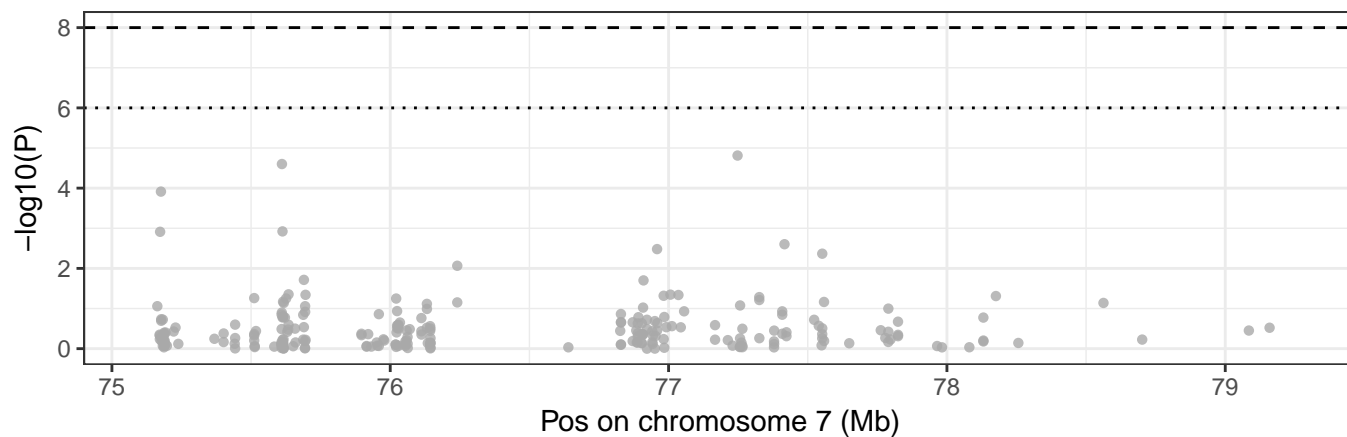

Supplement: S7 Fig — This figure shows three datasets: Results from the HapMap and the exome chip study, and imputed summary statistics. The top window shows HapMap P-values as orange circles and the imputed P-values (using summary statistics imputation) as solid circles, with the colour representing the imputation quality (only r^pred,adj2≥0.3 shown). The bottom window shows exome chip study results as solid, grey dots. Each dot represents the summary statistics of one variant. The x-axis shows the position (in Mb) on a ≥ 2 Mb range and the y-axis the −log10(P)-value. The horizontal line shows the P-value threshold of 10−6 (dotted) and 10−8 (dashed). Top and bottom window have annotated summary statistics: In the bottom window we mark dots as black if it is are part of the 122 reported hits of [13]. In the top window we mark the rs-id of variants that are part of the 122 reported variants of [13] in bold black, and if they are part of the 697 variants of [12] in bold orange font. Variants that are black (plain) are imputed variants (that had the lowest conditional P-value). Variants in orange (plain) are HapMap variants, but were not among the 697 reported hits. Each of the annotated variants is marked for clarity with a bold circle in the respective colour. The genes annotated in the middle window are printed in grey if the gene has a length < 5′000 bp or is an unrecognised gene (RP-). (ZIP) [file pgen.1007371.s007.zip › locuszoomplot/LOCUSZOOM_locusnbr-28_7-77106797_rs2868777.pdf]

# Candidate locus # 34

## HapMap and imputation results

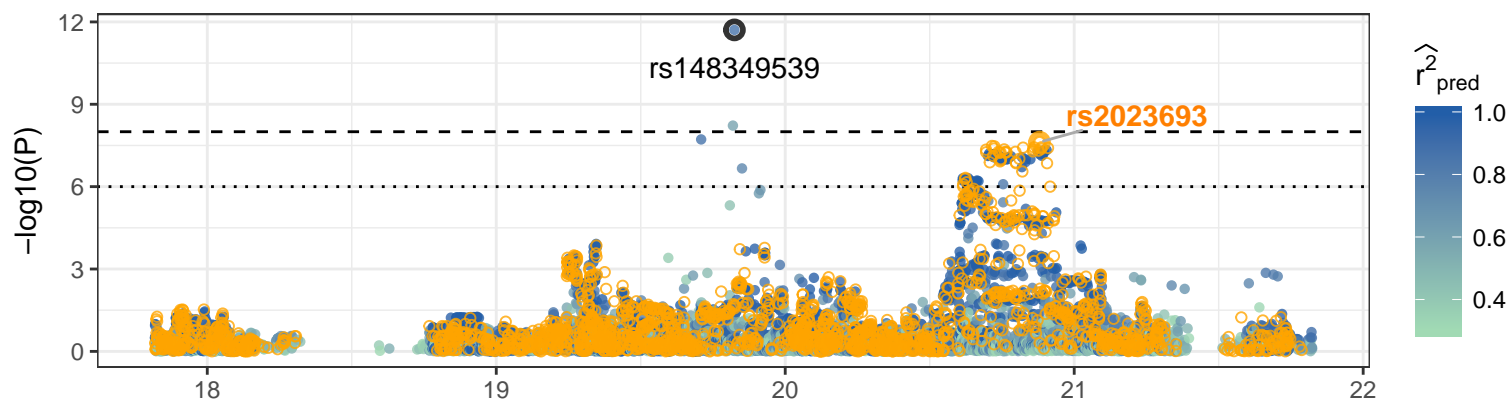

## Genes

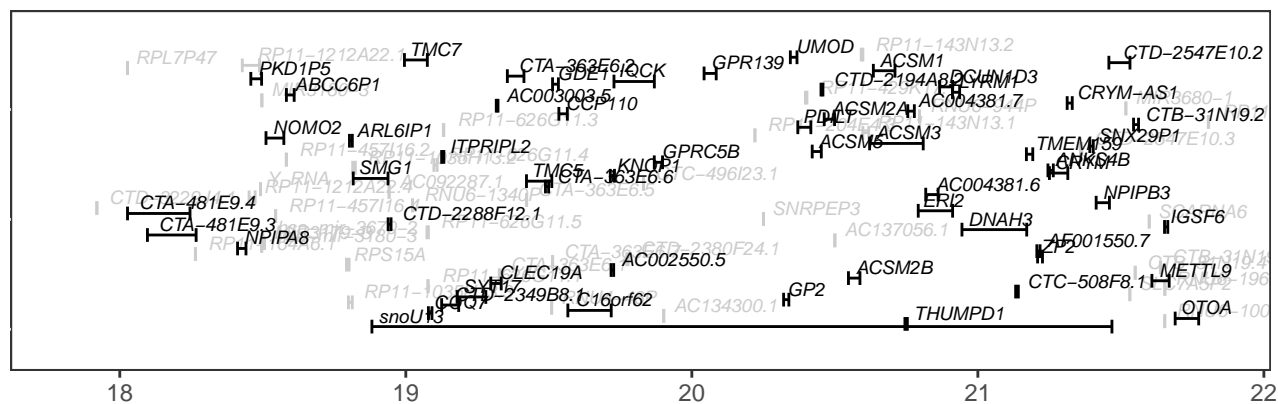

## Exome results

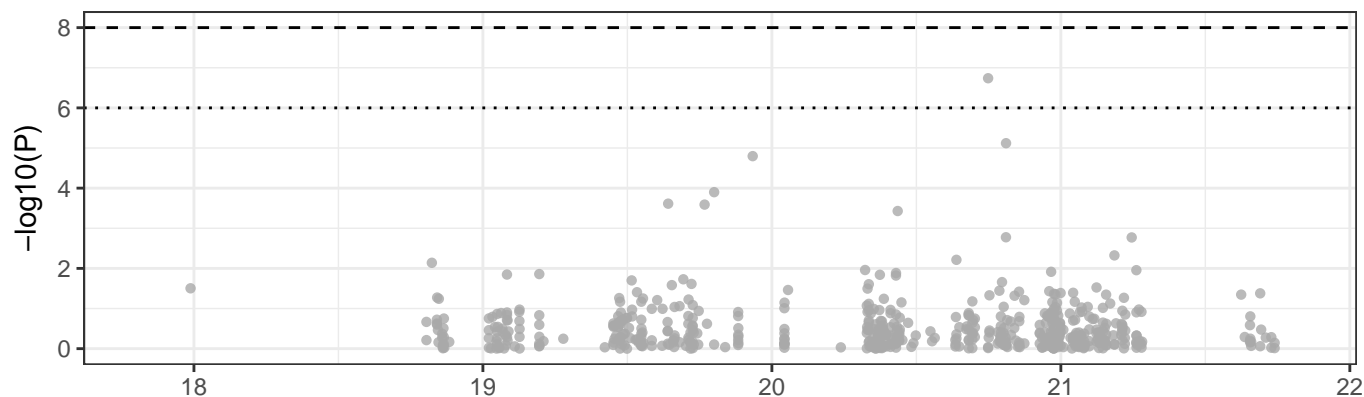

Pos on chromosome 16 (Mb)

Supplement: S7 Fig — This figure shows three datasets: Results from the HapMap and the exome chip study, and imputed summary statistics. The top window shows HapMap P-values as orange circles and the imputed P-values (using summary statistics imputation) as solid circles, with the colour representing the imputation quality (only r^pred,adj2≥0.3 shown). The bottom window shows exome chip study results as solid, grey dots. Each dot represents the summary statistics of one variant. The x-axis shows the position (in Mb) on a ≥ 2 Mb range and the y-axis the −log10(P)-value. The horizontal line shows the P-value threshold of 10−6 (dotted) and 10−8 (dashed). Top and bottom window have annotated summary statistics: In the bottom window we mark dots as black if it is are part of the 122 reported hits of [13]. In the top window we mark the rs-id of variants that are part of the 122 reported variants of [13] in bold black, and if they are part of the 697 variants of [12] in bold orange font. Variants that are black (plain) are imputed variants (that had the lowest conditional P-value). Variants in orange (plain) are HapMap variants, but were not among the 697 reported hits. Each of the annotated variants is marked for clarity with a bold circle in the respective colour. The genes annotated in the middle window are printed in grey if the gene has a length < 5′000 bp or is an unrecognised gene (RP-). (ZIP) [file pgen.1007371.s007.zip › locuszoomplot/LOCUSZOOM_locusnbr-34_16-19824164_rs148349539.pdf]

# Candidate locus # 12

## HapMap and imputation results

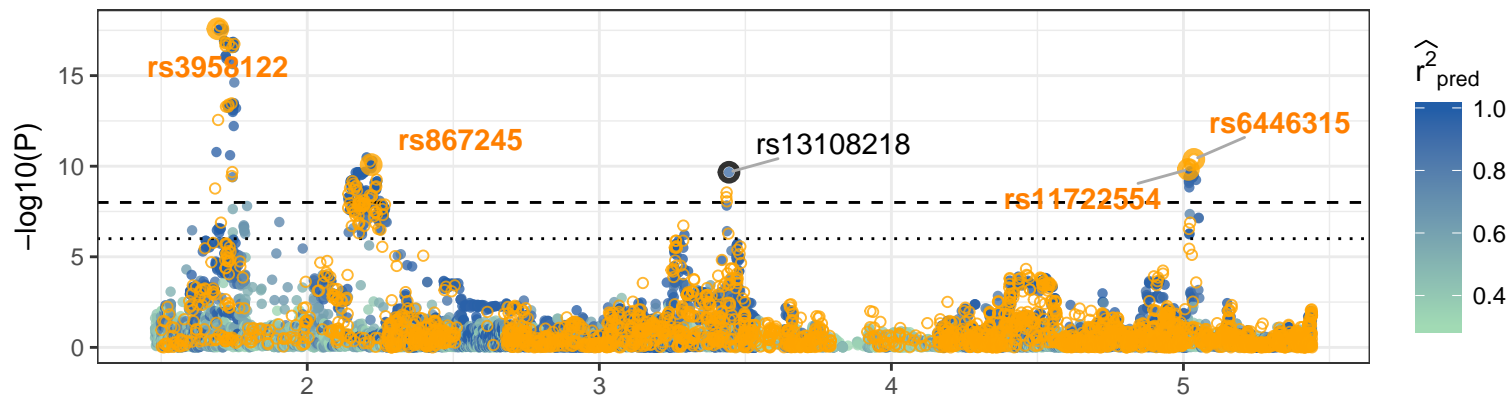

## Genes

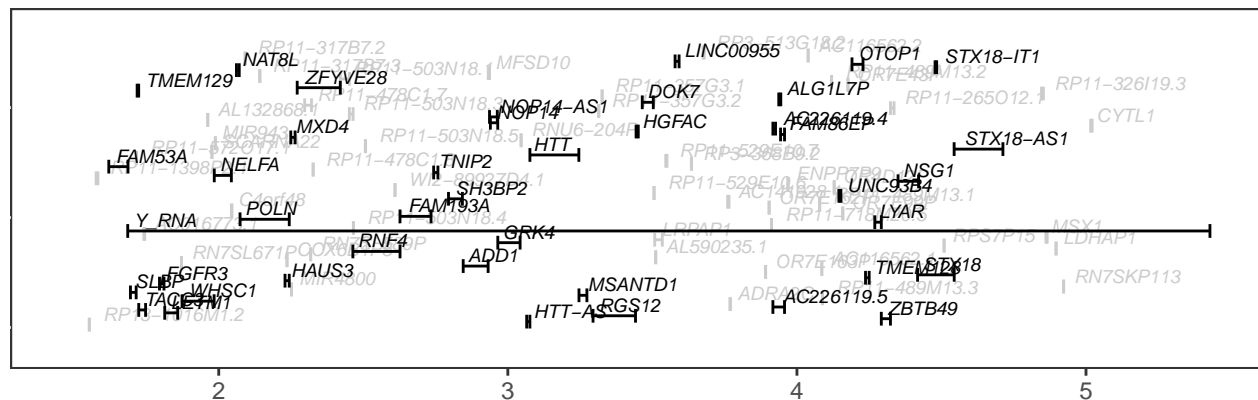

## Exome results

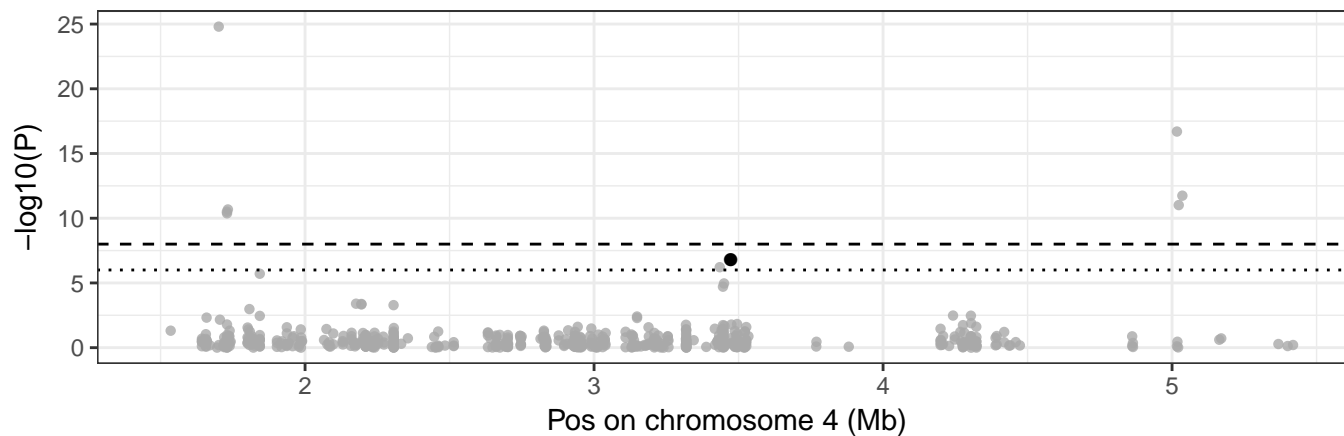

Supplement: S7 Fig — This figure shows three datasets: Results from the HapMap and the exome chip study, and imputed summary statistics. The top window shows HapMap P-values as orange circles and the imputed P-values (using summary statistics imputation) as solid circles, with the colour representing the imputation quality (only r^pred,adj2≥0.3 shown). The bottom window shows exome chip study results as solid, grey dots. Each dot represents the summary statistics of one variant. The x-axis shows the position (in Mb) on a ≥ 2 Mb range and the y-axis the −log10(P)-value. The horizontal line shows the P-value threshold of 10−6 (dotted) and 10−8 (dashed). Top and bottom window have annotated summary statistics: In the bottom window we mark dots as black if it is are part of the 122 reported hits of [13]. In the top window we mark the rs-id of variants that are part of the 122 reported variants of [13] in bold black, and if they are part of the 697 variants of [12] in bold orange font. Variants that are black (plain) are imputed variants (that had the lowest conditional P-value). Variants in orange (plain) are HapMap variants, but were not among the 697 reported hits. Each of the annotated variants is marked for clarity with a bold circle in the respective colour. The genes annotated in the middle window are printed in grey if the gene has a length < 5′000 bp or is an unrecognised gene (RP-). (ZIP) [file pgen.1007371.s007.zip › locuszoomplot/LOCUSZOOM_locusnbr-12_4-3443931_rs13108218.pdf]

# Candidate locus # 7

## HapMap and imputation results

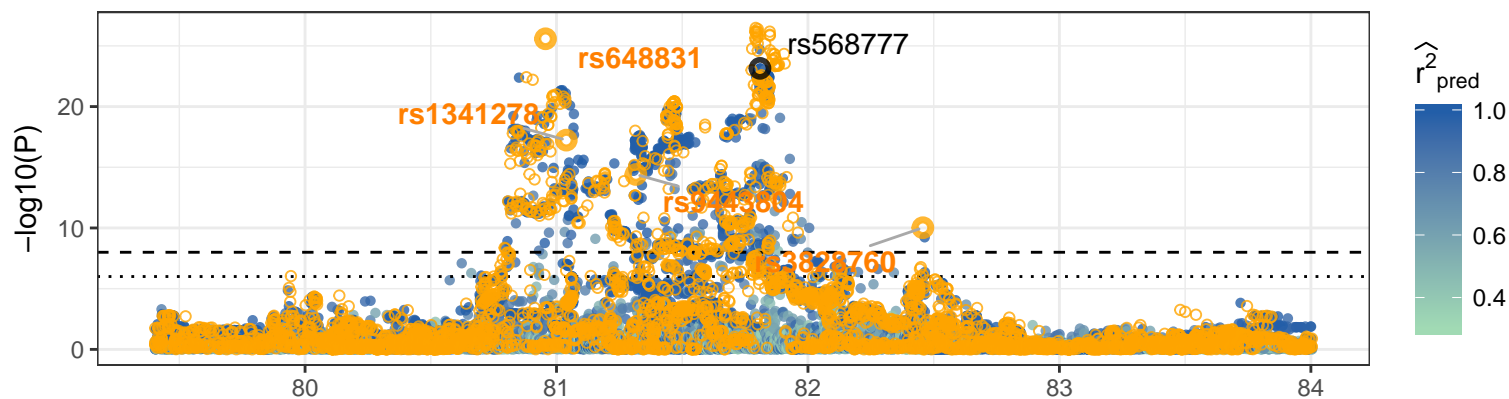

## Genes

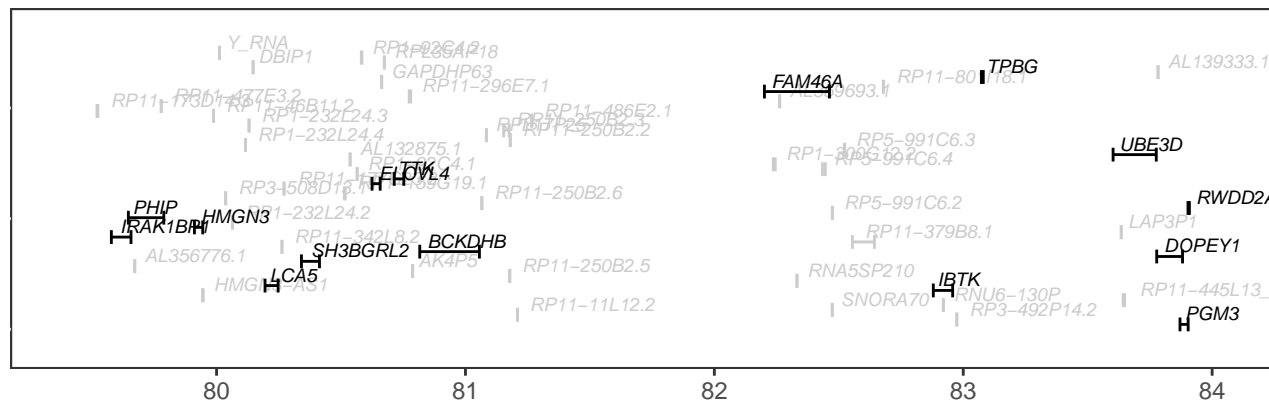

## Exome results

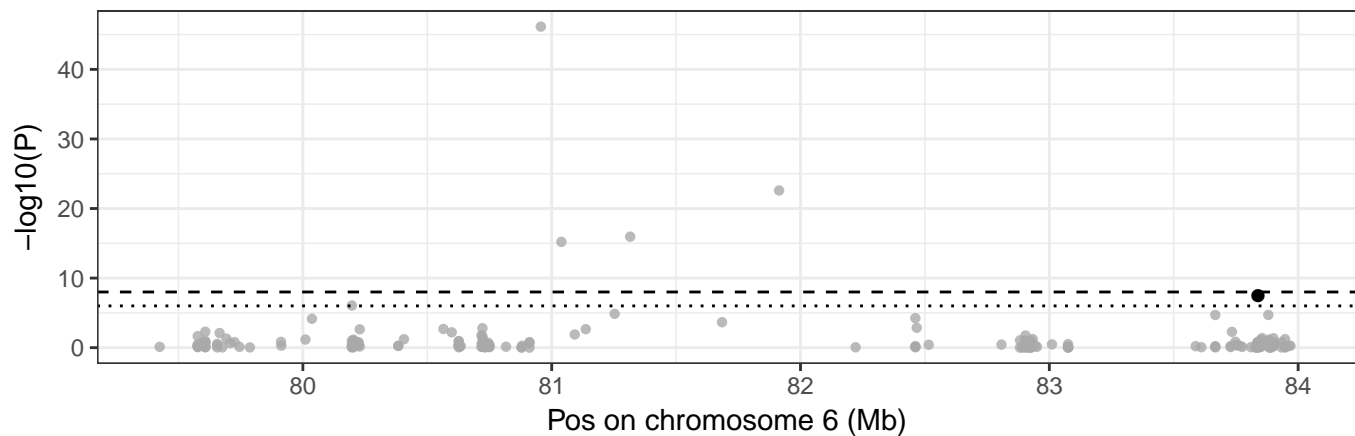

Supplement: S7 Fig — This figure shows three datasets: Results from the HapMap and the exome chip study, and imputed summary statistics. The top window shows HapMap P-values as orange circles and the imputed P-values (using summary statistics imputation) as solid circles, with the colour representing the imputation quality (only r^pred,adj2≥0.3 shown). The bottom window shows exome chip study results as solid, grey dots. Each dot represents the summary statistics of one variant. The x-axis shows the position (in Mb) on a ≥ 2 Mb range and the y-axis the −log10(P)-value. The horizontal line shows the P-value threshold of 10−6 (dotted) and 10−8 (dashed). Top and bottom window have annotated summary statistics: In the bottom window we mark dots as black if it is are part of the 122 reported hits of [13]. In the top window we mark the rs-id of variants that are part of the 122 reported variants of [13] in bold black, and if they are part of the 697 variants of [12] in bold orange font. Variants that are black (plain) are imputed variants (that had the lowest conditional P-value). Variants in orange (plain) are HapMap variants, but were not among the 697 reported hits. Each of the annotated variants is marked for clarity with a bold circle in the respective colour. The genes annotated in the middle window are printed in grey if the gene has a length < 5′000 bp or is an unrecognised gene (RP-). (ZIP) [file pgen.1007371.s007.zip › locuszoomplot/LOCUSZOOM_locusnbr-7_6-81809121_rs568777.pdf]

# Candidate locus # 13

## HapMap and imputation results

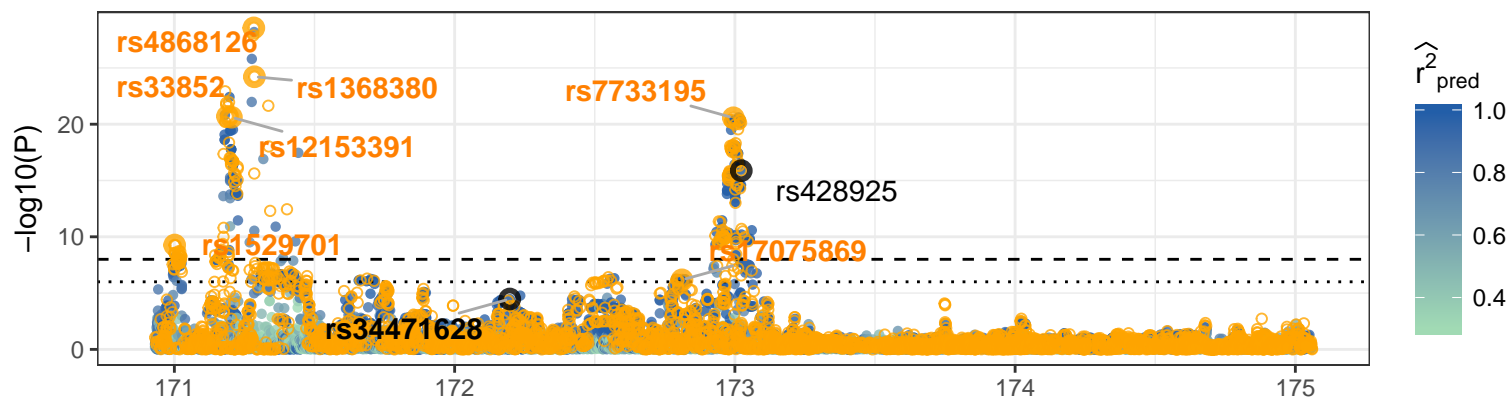

## Genes

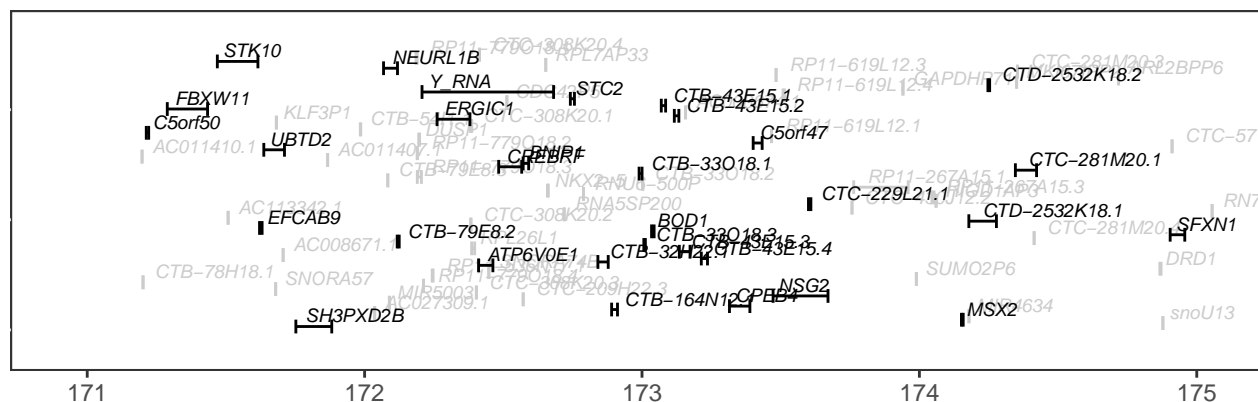

## Exome results

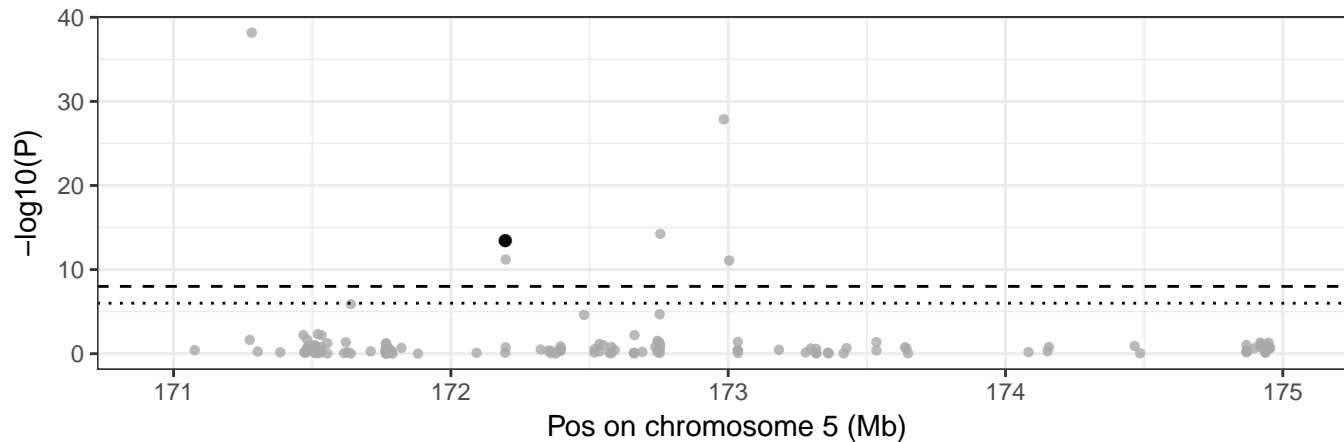

Supplement: S7 Fig — This figure shows three datasets: Results from the HapMap and the exome chip study, and imputed summary statistics. The top window shows HapMap P-values as orange circles and the imputed P-values (using summary statistics imputation) as solid circles, with the colour representing the imputation quality (only r^pred,adj2≥0.3 shown). The bottom window shows exome chip study results as solid, grey dots. Each dot represents the summary statistics of one variant. The x-axis shows the position (in Mb) on a ≥ 2 Mb range and the y-axis the −log10(P)-value. The horizontal line shows the P-value threshold of 10−6 (dotted) and 10−8 (dashed). Top and bottom window have annotated summary statistics: In the bottom window we mark dots as black if it is are part of the 122 reported hits of [13]. In the top window we mark the rs-id of variants that are part of the 122 reported variants of [13] in bold black, and if they are part of the 697 variants of [12] in bold orange font. Variants that are black (plain) are imputed variants (that had the lowest conditional P-value). Variants in orange (plain) are HapMap variants, but were not among the 697 reported hits. Each of the annotated variants is marked for clarity with a bold circle in the respective colour. The genes annotated in the middle window are printed in grey if the gene has a length < 5′000 bp or is an unrecognised gene (RP-). (ZIP) [file pgen.1007371.s007.zip › locuszoomplot/LOCUSZOOM_locusnbr-13_5-173022921_rs428925.pdf]

# Candidate locus # 2

## HapMap and imputation results

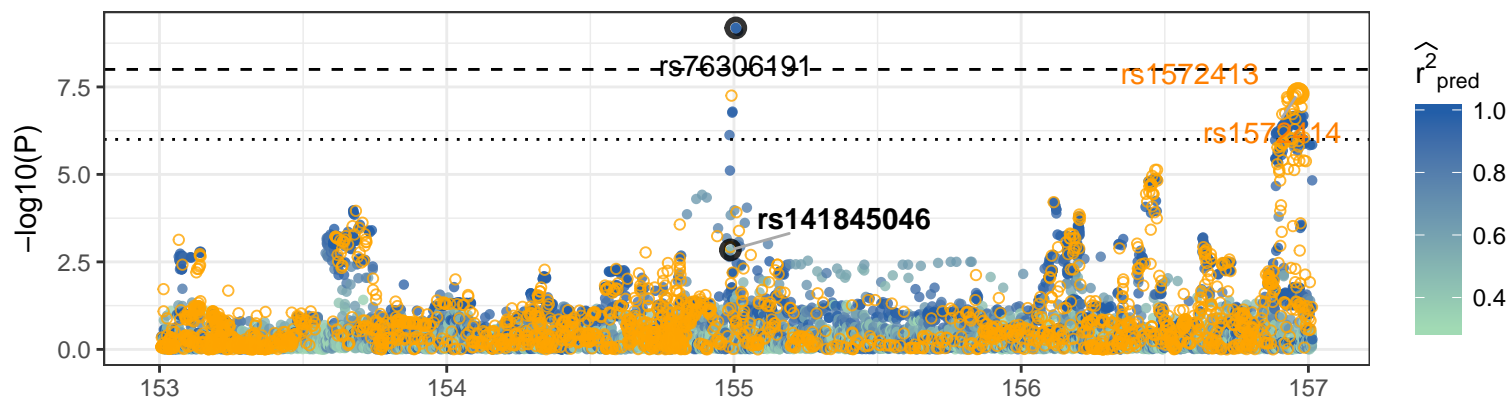

## Genes

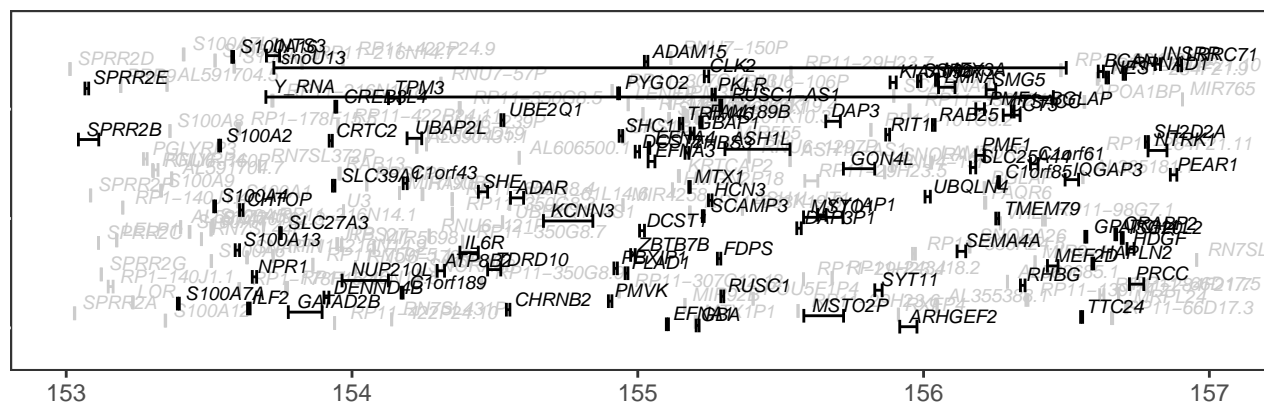

## Exome results

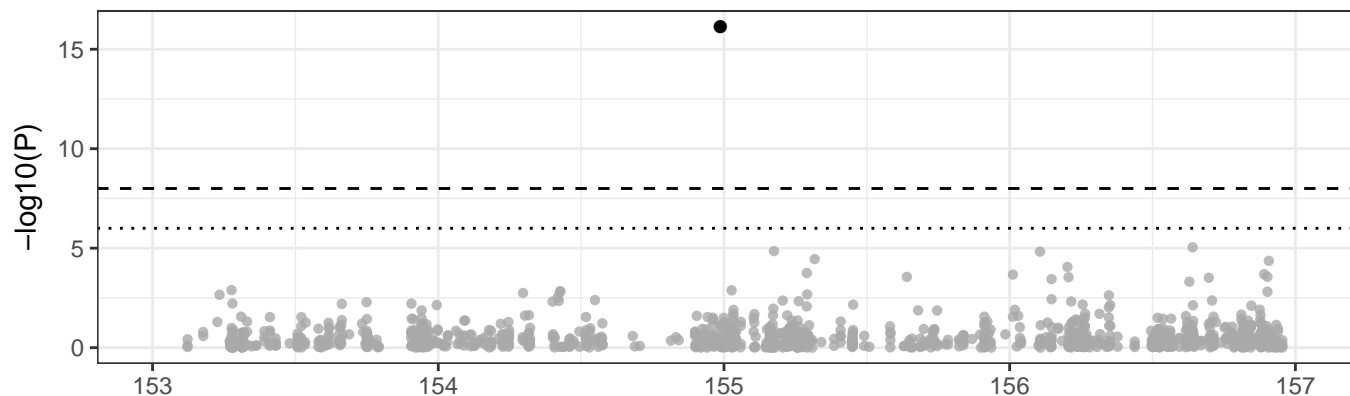

Pos on chromosome 1 (Mb)

Supplement: S7 Fig — This figure shows three datasets: Results from the HapMap and the exome chip study, and imputed summary statistics. The top window shows HapMap P-values as orange circles and the imputed P-values (using summary statistics imputation) as solid circles, with the colour representing the imputation quality (only r^pred,adj2≥0.3 shown). The bottom window shows exome chip study results as solid, grey dots. Each dot represents the summary statistics of one variant. The x-axis shows the position (in Mb) on a ≥ 2 Mb range and the y-axis the −log10(P)-value. The horizontal line shows the P-value threshold of 10−6 (dotted) and 10−8 (dashed). Top and bottom window have annotated summary statistics: In the bottom window we mark dots as black if it is are part of the 122 reported hits of [13]. In the top window we mark the rs-id of variants that are part of the 122 reported variants of [13] in bold black, and if they are part of the 697 variants of [12] in bold orange font. Variants that are black (plain) are imputed variants (that had the lowest conditional P-value). Variants in orange (plain) are HapMap variants, but were not among the 697 reported hits. Each of the annotated variants is marked for clarity with a bold circle in the respective colour. The genes annotated in the middle window are printed in grey if the gene has a length < 5′000 bp or is an unrecognised gene (RP-). (ZIP) [file pgen.1007371.s007.zip › locuszoomplot/LOCUSZOOM_locusnbr-2_1-155006451_rs76306191.pdf]

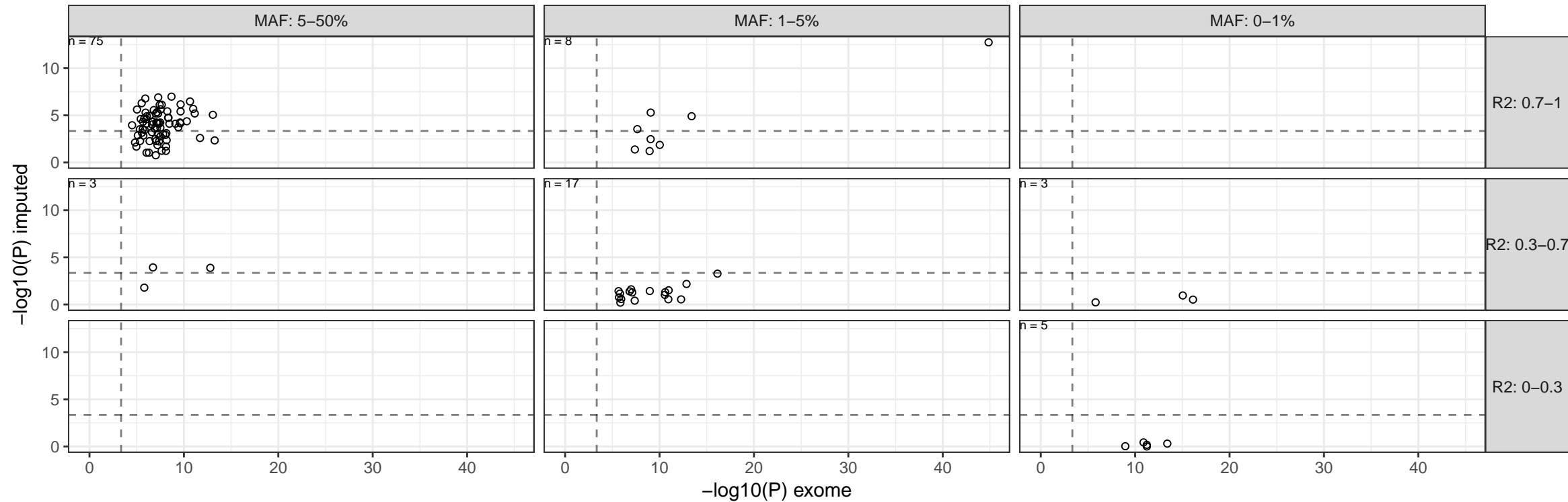

Supplement: S8 Fig — This graph shows for all 111 variants the −log10(p)-value of the exome chip study on the x-axis and the imputed −log10(p)-value on the y-axis. The first row refers to the highest imputation quality (between 0.7 and 1), with the columns as the different allele frequency categories. The number of dots in each window is marked top left. The vertical and horizontal dotted lines mark the significance threshold of −log10(0.05/111) (dashed). The width of the x-axis is proportional to the range of the y-axis. For MAF and r^pred,adj2 notation, the lower bound is excluded while the upper bound is included. For example, 1 − 5% is equivalent to 1 < MAF ≤ 5. (PDF) [file pgen.1007371.s008.pdf]

Null SNVs  
P-value distribution (SSimp)

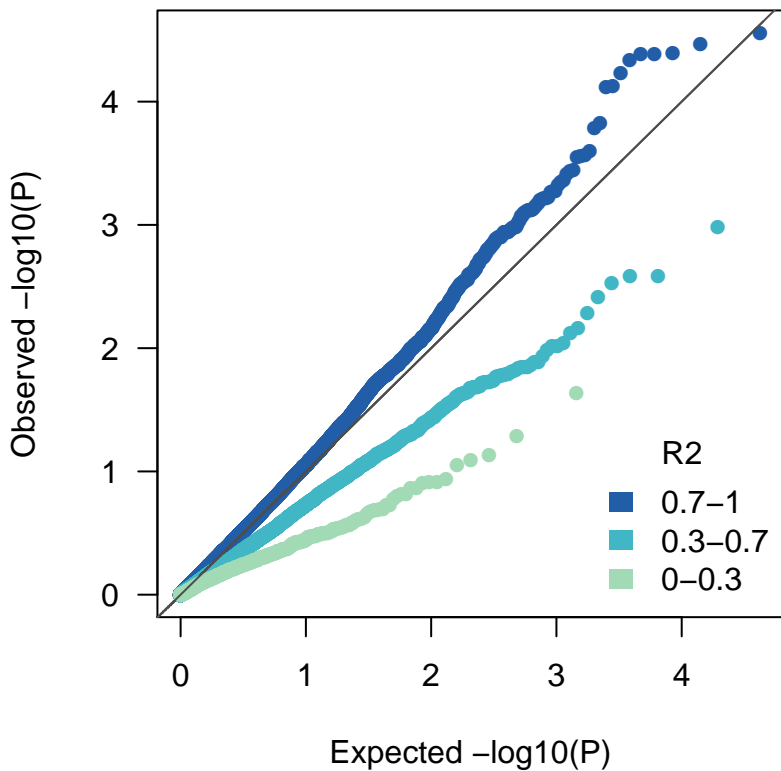

Supplement: S9 Fig — These QQ-plots show the distribution of p-values resulting from summary statistics imputation, for associated variants (left window), null variants (right window). The colours refer to the imputation quality categories. Note that the P-value in these plots are not λGC corrected. (PDF) [file pgen.1007371.s009.pdf]

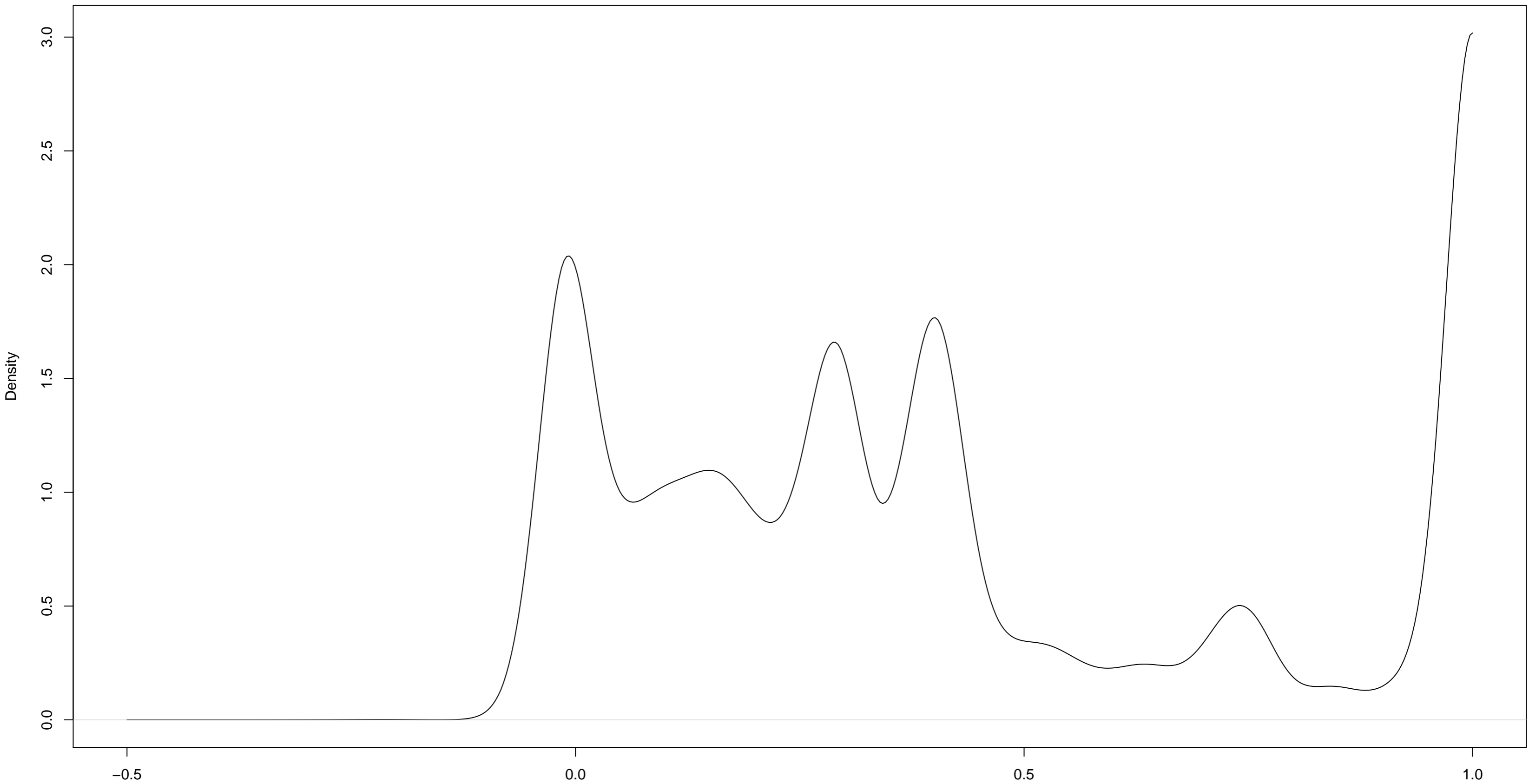

Supplement: S10 Fig — In the GIANT meta-analysis (BMI, women over 50 years of age) the set of SNVs is different in each cohort, allowing us to create a binary ‘missingness’ vector for each SNV recording whether a given individual in the combined population was genotyped for this SNV. For 10′000 randomly selected pairs of nearby SNVs, we compute the correlation between these missingness vectors and plot the density plot. The correlations are usually greater than zero, and often quite close to one, confirming that a ‘missing independently at random’ assumption is not appropriate. (PDF) [file pgen.1007371.s010.pdf]

# GTimp vs SSimp (associated SNVs)

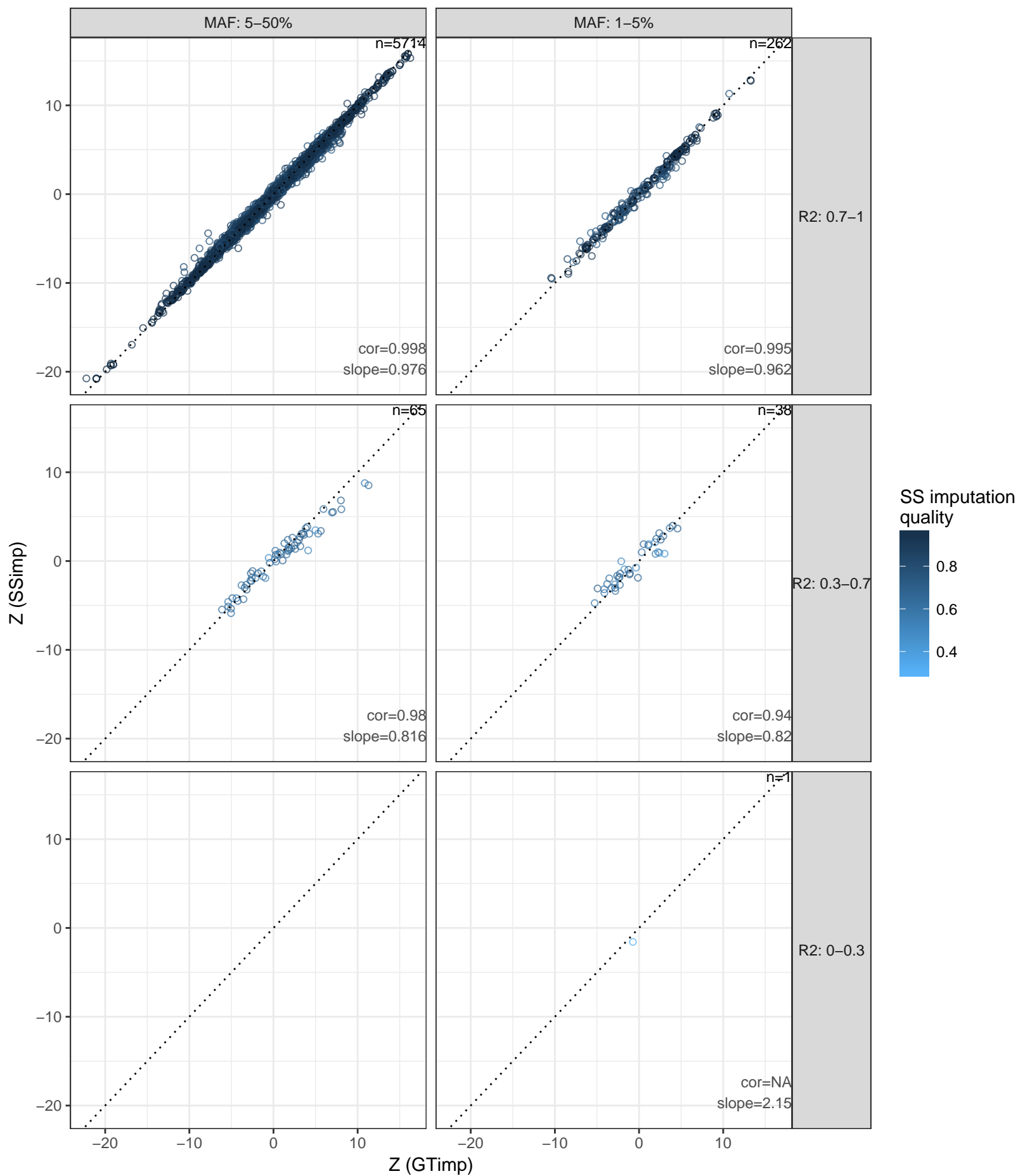

Supplement: S11 Fig — The x-axis shows the Z-statistics of the genotype imputation summary statistics, while the y-axis shows the Z-statistics from summary statistics imputation. The color of each point refers to the imputation quality of summary statistics imputation. Results are grouped according to MAF (columns) and imputation quality (rows) categories and the numbers top-right in each window refers to the number of SNVs represented. The identity line is indicated with a dotted line. The estimation for correlation and slope are noted in the bottom-right corner. (PDF) [file pgen.1007371.s011.pdf]

# GTime vs SSimp (null SNVs)

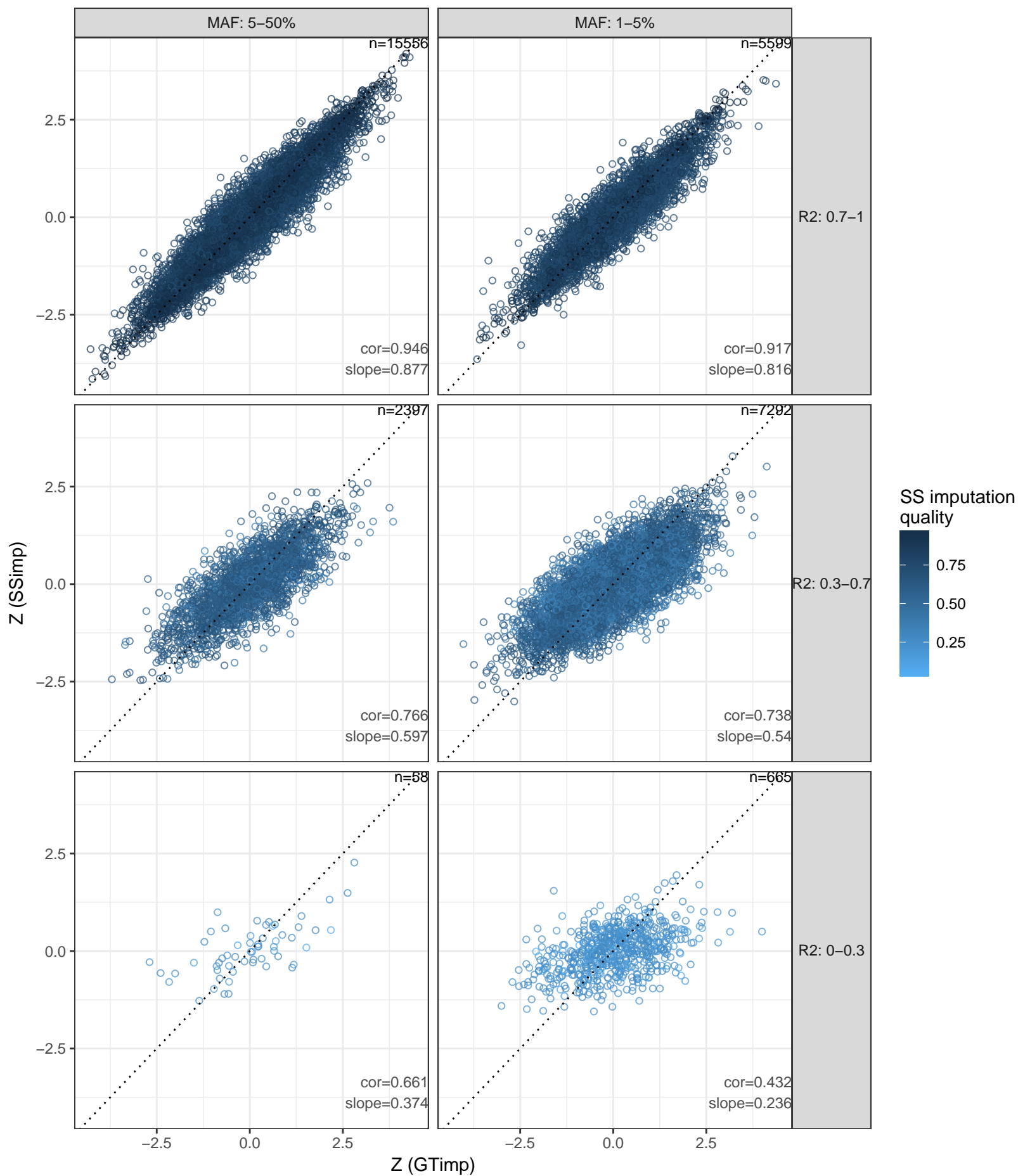

Supplement: S12 Fig — The x-axis shows the Z-statistics of the genotype imputation summary statistics, while the y-axis shows the Z-statistics from summary statistics imputation. The color of each point refers to the imputation quality of summary statistics imputation. Results are grouped according to MAF (columns) and imputation quality (rows) categories and the numbers top-right in each window refers to the number of SNVs represented. The identity line is indicated with a dotted line. The estimation for correlation and slope are noted in the bottom-right corner. (PDF) [file pgen.1007371.s012.pdf]

# GTimp vs SSimp (associated SNVs)

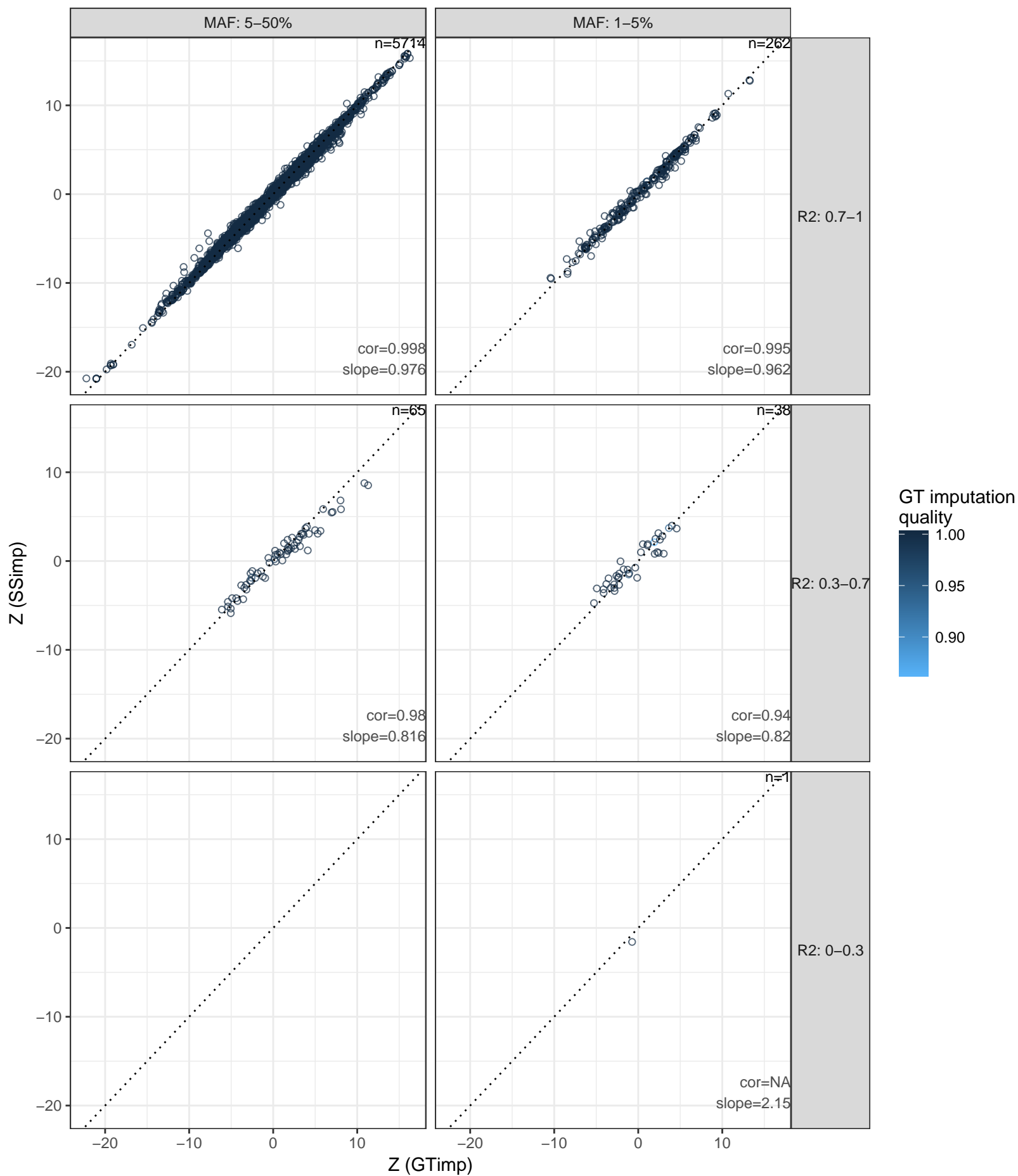

Supplement: S13 Fig — The x-axis shows the Z-statistics of the genotype imputation summary statistics, while the y-axis shows the Z-statistics from summary statistics imputation. The color of each point refers to the imputation quality of genotype imputation. Results are grouped according to MAF (columns) and imputation quality (rows) categories and the numbers top-right in each window refers to the number of SNVs represented. The identity line is indicated with a dotted line. The estimation for correlation and slope are noted in the bottom-right corner. (PDF) [file pgen.1007371.s013.pdf]

# GTime vs SSimp (null SNVs)

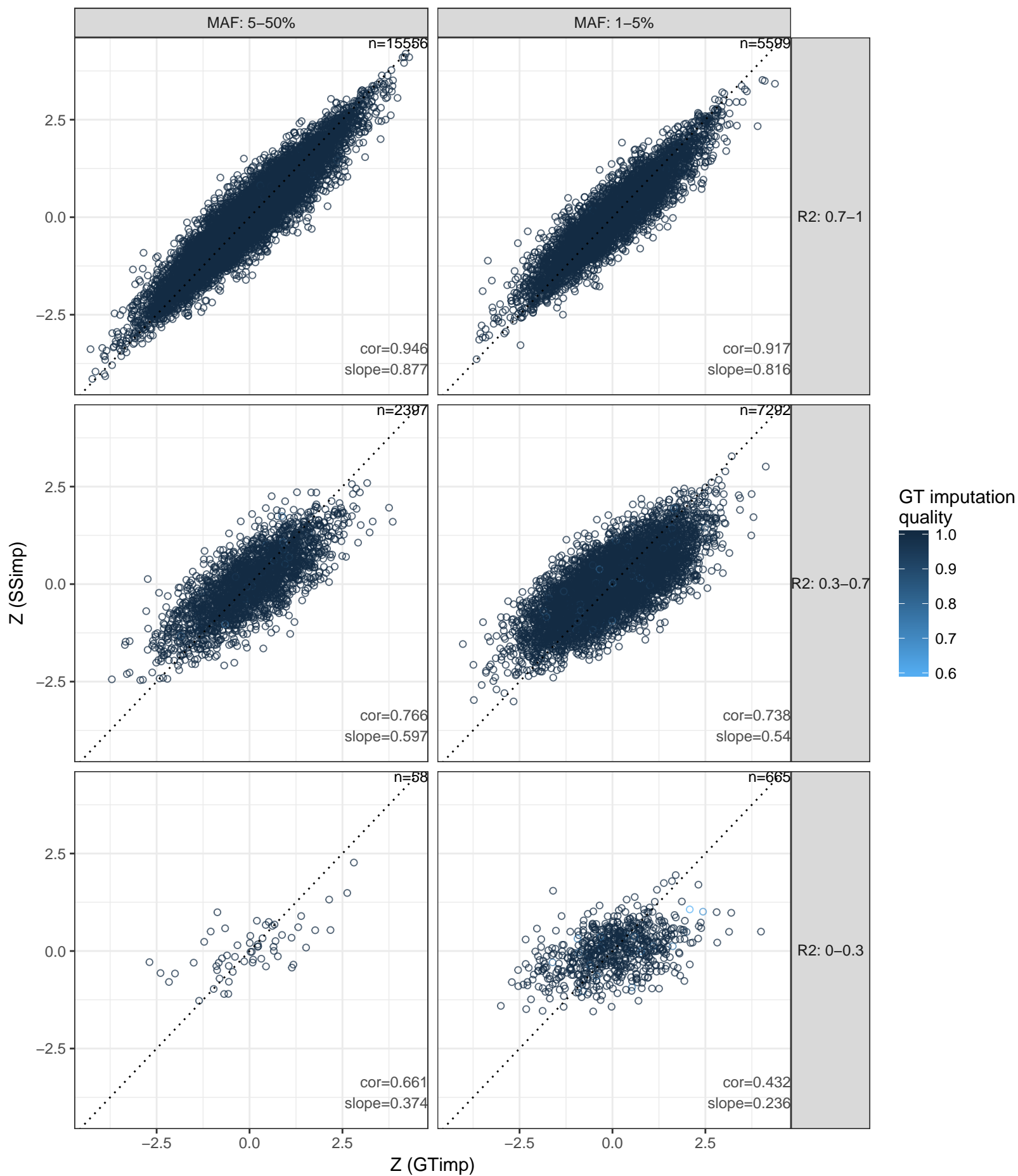

Supplement: S14 Fig — The x-axis shows the Z-statistics of the genotype imputation summary statistics, while the y-axis shows the Z-statistics from summary statistics imputation. The color of each point refers to the imputation quality of genotype imputation. Results are grouped according to MAF (columns) and imputation quality (rows) categories and the numbers top-right in each window refers to the number of SNVs represented. The identity line is indicated with a dotted line. The estimation for correlation and slope are noted in the bottom-right corner. (PDF) [file pgen.1007371.s014.pdf]

GIANT imputation: C vs. D

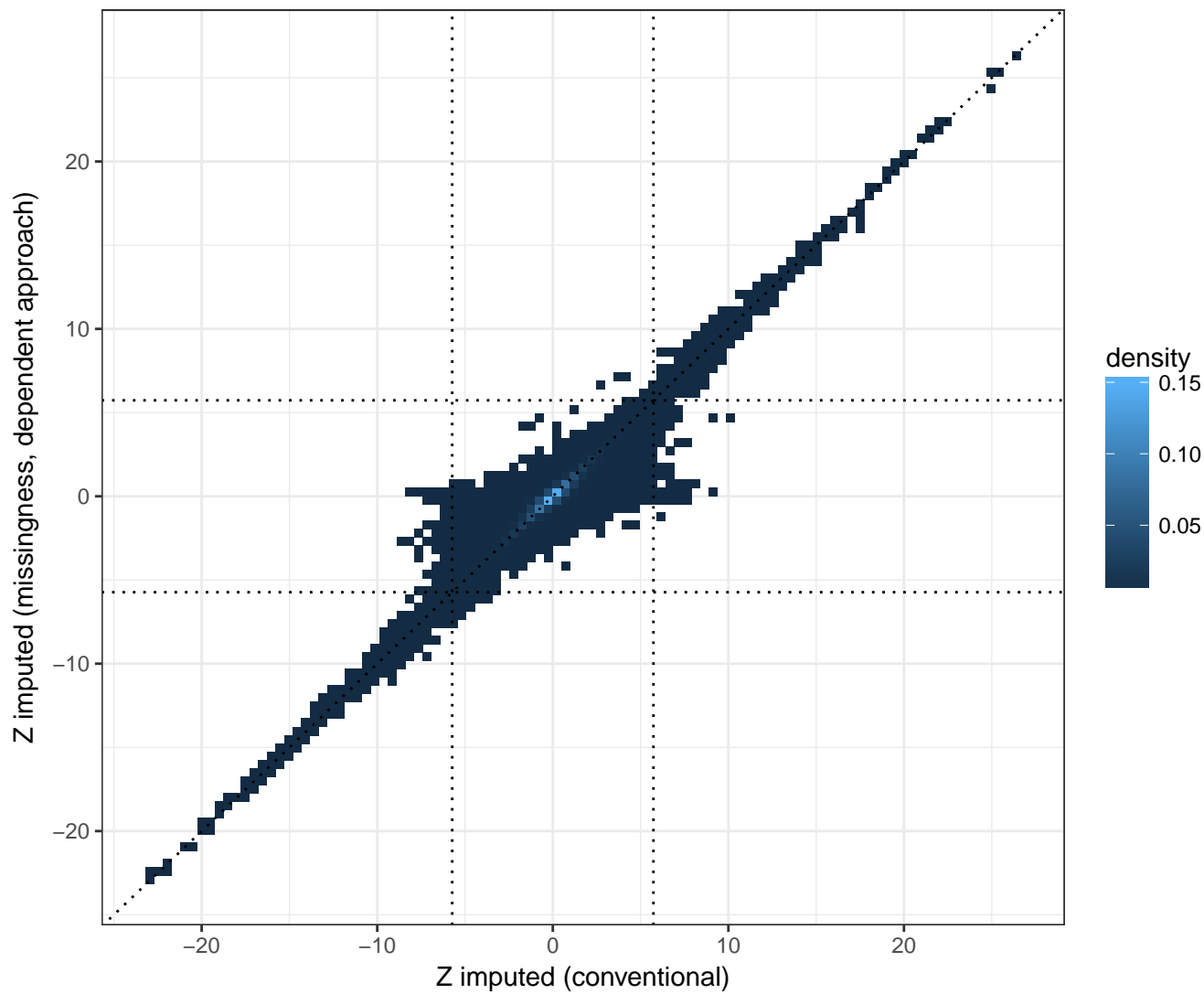

Supplement: S15 Fig — The x-axis shows the Z-statistics of the conventional estimate, the y-axis the Z-statistics when accounting for missingness (dependent approach). The dotted line marks the genome-wide threshold. There are 11′200′403 variants displayed in a binned fashion. (PDF) [file pgen.1007371.s015.pdf]
