# Supplementary material for: Evaluation and application of summary statistic imputation to discover new height-associated loci
Source: PLoS Genet. 2018 May 21;14(5):e1007371. doi: 10.1371/journal.pgen.1007371 (PMC5983877; doi:10.1371/journal.pgen.1007371)
Supplement: S7 Fig — This figure shows three datasets: Results from the HapMap and the exome chip study, and imputed summary statistics. The top window shows HapMap P-values as orange circles and the imputed P-values (using summary statistics imputation) as solid circles, with the colour representing the imputation quality (only r^pred,adj2≥0.3 shown). The bottom window shows exome chip study results as solid, grey dots. Each dot represents the summary statistics of one variant. The x-axis shows the position (in Mb) on a ≥ 2 Mb range and the y-axis the −log10(P)-value. The horizontal line shows the P-value threshold of 10−6 (dotted) and 10−8 (dashed). Top and bottom window have annotated summary statistics: In the bottom window we mark dots as black if it is are part of the 122 reported hits of [13]. In the top window we mark the rs-id of variants that are part of the 122 reported variants of [13] in bold black, and if they are part of the 697 variants of [12] in bold orange font. Variants that are black (plain) are imputed variants (that had the lowest conditional P-value). Variants in orange (plain) are HapMap variants, but were not among the 697 reported hits. Each of the annotated variants is marked for clarity with a bold circle in the respective colour. The genes annotated in the middle window are printed in grey if the gene has a length < 5′000 bp or is an unrecognised gene (RP-). (ZIP) [file pgen.1007371.s007.zip › locuszoomplot/LOCUSZOOM_locusnbr-33_13-33119541_rs11617933.pdf]

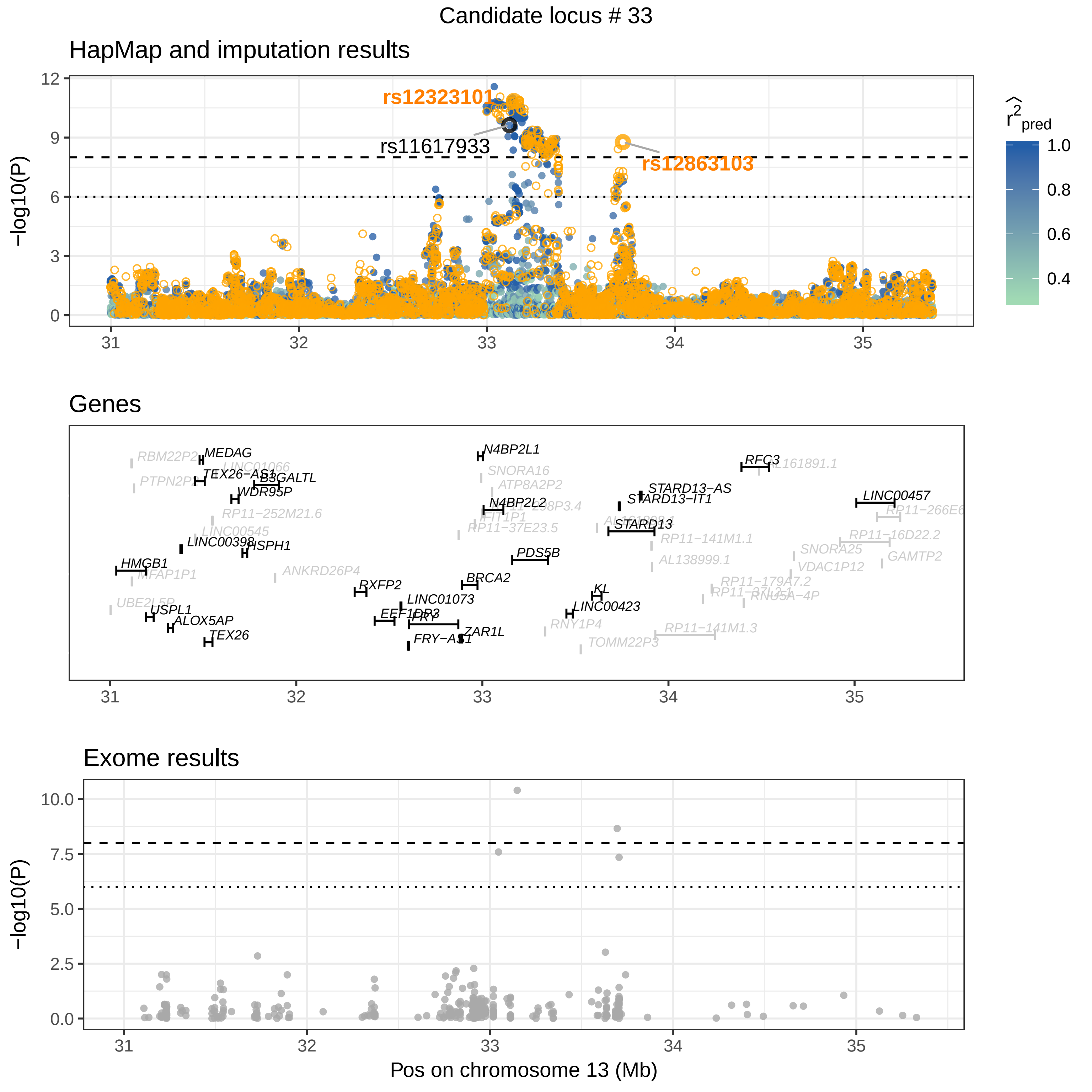
Candidate locus # 33
HapMap and imputation results
rs12323101
rs11617933
rs12863103
−log10(P)
$\widehat{r^2_{pred}}$
1.0
0.8
0.6
0.4
12
9
6
3
0
31
32
33
34
35
Genes
RBM22P2
MEDAG
LINC01066
PTPN2P1
TEX26−AS1
B3GALTL
WDR95P
RP11−252M21.6
LINC00545
LINC00398
HSPH1
HMGB1
MFAP1P1
ANKRD26P4
UBE2L5P
USPL1
ALOX5AP
TEX26
RXFP2
LINC01073
EEF1DP3
FRY
FRY−AS1
ZAR1L
BRCA2
N4BP2L1
SNORA16
ATP8A2P2
N4BP2L2
IFIT1P1
RP11−37E23.5
PDS5B
STARD13−AS
STARD13−IT1
STARD13
RP11−141M1.1
AL138999.1
KL
LINC00423
RNY1P4
TOMM22P3
RP11−141M1.3
RFC3
RP11−179A7.2
RNU6A−4P
LINC00457
RP11−266E6
RP11−16D22.2
SNORA25
GAMTP2
VDAC1P12
Exome results
−log10(P)
10.0
7.5
5.0
2.5
0.0
Pos on chromosome 13 (Mb)
