# Supplementary material for: Evaluation and application of summary statistic imputation to discover new height-associated loci
Source: PLoS Genet. 2018 May 21;14(5):e1007371. doi: 10.1371/journal.pgen.1007371 (PMC5983877; doi:10.1371/journal.pgen.1007371)
Supplement: S1 Appendix — (PDF) [file pgen.1007371.s019.pdf]

Evaluation and application of summary statistic imputation to discover new height-associated loci

Sina Rüeger, Aaron McDaid and Zoltán Kutalik

March 13, 2018

S1 Appendix: Simulation of GWAS summary statistics

For simulation of GWAS summary statistics we used data from the five European subpopulations `CEU, GBR, FIN, TSI` and `IBR` of the 1000 Genomes project (1KG). We chose to up-sample chromosome 15 using HAPGEN2 (Su et al. 2011) to $5'000$ individuals for each subpopulation, yielding a total of $25'000$ individuals. Of these 5000 individuals per population we used half each to generate a GWAS with an *in silico* phenotype. The remaining $12'500$ individuals were used as reference panel for summary statistic imputation. The HapMap SNVs were taken as tag SNVs.
We split chromosome 15 into 82 disjoint regions of 1.5 Mb, of which we used 40 for the simulation. In each region we chose a causal variant $\boldsymbol{g}$ randomly from all SNVs with minor allele frequency between 0.05 and 0.2. We simulated an *in silico* phenotype $\boldsymbol{y}$ using a normal linear model $\boldsymbol{y} = \beta \boldsymbol{g} + \varepsilon$ with $\varepsilon \sim \mathcal{N}(0, 1 - \beta^2 \cdot 2q(1-q))$, where q is the allele frequency of the causal SNV and β was selected such that the explained variance $\beta^2 \cdot 2q(1-q)$ is set to 0.02. For the specific scenario of null SNVs, β was set to 0. To obtain the association summary statistics we ran linear regression for each variant k in the 1.5 Mb region, yielding effect size and standard error estimates a_k, s_k. We did not include regions at the very start of the chromosome, as it had ambiguous correlation structure.

Simulations with variable missingness patterns

The same strategy as described above was used here. To assign sample sizes to the tag SNVs, the sample sizes were taken from the largest HDL study (Global Lipids Genetics Consortium 2013) (bottom row of Fig. 3) or a large T2D study (Morris et al. 2012) (top row), after those sample sizes were scaled to the range 0-12'500; Where a tag SNV was not present in the HDL/T2D study, a sample size was copied from another SNV at random.

For each SNV, a binary 'missingness' vector of length 12'500 was constructed randomly to decide which individuals are simulated as 'missing' for this SNV. This requires a missingness correlation parameter, θ_{miss}. Among the first N_k elements of the vector for SNV k , $(1{-}\theta_{miss}) \times N_k \times \frac{N_{max}-N_k}{N_{max}}$ (rounded down) were selected randomly to be 'missing'. Among the remaining $N_{max}{-}N_k$ individuals,

$$\left(\theta_{miss} \times (N_{max}{-}N_k) + (1 - \theta_{miss}) \times (N_{max}{-}N_k) \times \frac{N_{max} - N_k}{N_{max}}\right)$$

(rounded up) were selected randomly to be 'missing'. These two numbers are constructed such that the total missing is $N_{max}{-}N_k$. When $\theta_{miss}{=}0$, the missingness rate is the same in both portions ($\frac{N_{max}-N_k}{N_{max}}$), which corresponds to the simple missing-at-random model. $\theta_{miss}{=}1$ gives us the maximum possible correlation between the missingness vectors corresponding to pairs of SNVs, and therefore the maximum possible sample overlap.

The phenotype was generated as described before, however the Z-statistics for tag SNVs are no longer computed on all $12'500$ individuals and are instead computed via a regression on only the non-missing subset of the 12'500 individuals. Also the (partial) standardised effect estimates $\boldsymbol{a}'_{\mathcal{M}}$ were computed only on this subset of individuals; it is only these partial standardised effect estimates that are available to the estimators that we evaluate.

References

Global Lipids Genetics Consortium (2013). Discovery and refinement of loci associated with lipid levels. *Nature genetics*, 45(11):1274–83.

Morris, A. P. et al. (2012). Large-scale association analysis provides insights into the genetic architecture and pathophysiology of type 2 diabetes. *Nature Genetics*, 44(9):981–990.

Su, Z., Marchini, J., and Donnelly, P. (2011). HAPGEN2: simulation of multiple disease SNPs. *Bioinformatics*.
