# Supplementary material for: Evaluation and application of summary statistic imputation to discover new height-associated loci
Source: PLoS Genet. 2018 May 21;14(5):e1007371. doi: 10.1371/journal.pgen.1007371 (PMC5983877; doi:10.1371/journal.pgen.1007371)
Supplement: S2 Appendix — (PDF) [file pgen.1007371.s020.pdf]

Evaluation and application of summary statistic imputation to discover new height-associated loci

Sina Rüeger, Aaron McDaid and Zoltán Kutalik

March 13, 2018

S2 Appendix: Imputation quality

Imputation quality, r^2, is defined as the the squared correlation between the imputed and true genotype. Therefore, to verify Eq. 8 we followed a similar idea. We used the simulation data as presented in Appendix S1 under a null model. Along with the true effect size and the imputed effect size, we calculated for each SNV four different imputation qualities: (1) Eq. 7 with $\lambda = 0.1$ Pasaniuc et al. (2014), (2) Eq. 7 with $\lambda = 2/\sqrt{n}$, (3) Eq. 7 with $\lambda = 10^{-6}$, (4) Eq. 8 with $\lambda = 2/\sqrt{n}$. We then categorised each imputation quality into 20 bins ($[0, 0.05), [0.05, 1), \ldots, [0.95, 1]$). Next, we calculated within each reference panel size, imputation quality metrics (1-4) and imputation quality group the median imputation quality, and the squared correlation between the true and imputed effect size ($\text{cor}(\boldsymbol{b}, \boldsymbol{\beta})^2$). These two measures are plotted against each other in Fig. S1.

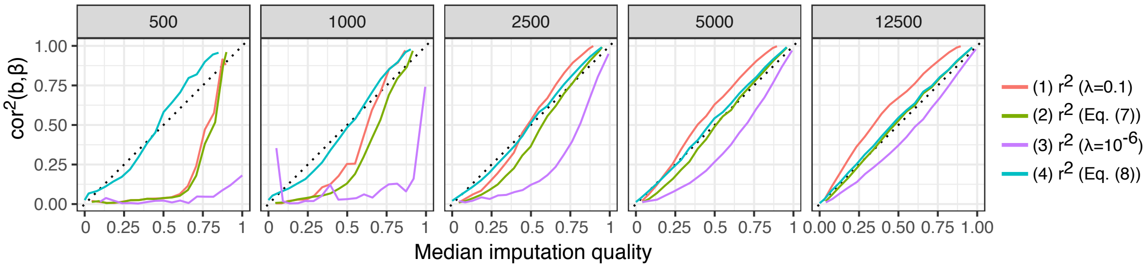

Figure S1: This figure shows the median imputation quality within each imputation quality interval (x-axis) versus the squared correlation between true and imputed effect size (y-axis). Each line consists of 20 points. The colors correspond to imputation quality measures: (1) Eq. 7 with $\lambda = 0.1$ Pasaniuc et al. (2014), (2) Eq. 7 with $\lambda = 2/\sqrt{n}$, (3) Eq. 7 with $\lambda = 10^{-6}$, (4) Eq. 8 with $\lambda = 2/\sqrt{n}$. The dashed line represents the identity line. The windows represent reference panel sizes in increasing order from left to right.

We observe that for all reference panel sizes, Eq. 8 (turquoise) offers the best concordance with the true imputation quality definition ($\text{cor}(\boldsymbol{b}, \boldsymbol{\beta})^2$).

References

Pasaniuc, B., Zaitlen, N., Shi, H., Bhatia, G., Gusev, A., Pickrell, J., Hirschhorn, J., Strachan, D. P., Patterson, N., and Price, A. L. (2014). Fast and accurate imputation of summary statistics enhances evidence of functional enrichment. *Bioinformatics*, 30(20).
