# Supplementary material for: Evaluation and application of summary statistic imputation to discover new height-associated loci
Source: PLoS Genet. 2018 May 21;14(5):e1007371. doi: 10.1371/journal.pgen.1007371 (PMC5983877; doi:10.1371/journal.pgen.1007371)
Supplement: S3 Appendix — (PDF) [file pgen.1007371.s021.pdf]

# Evaluation and application of summary statistic imputation to discover new height-associated loci

Sina Rüeger, Aaron McDaid and Zoltán Kutalik

March 13, 2018

## S3 Appendix: Accounting for varying sample size and missingness

All previously published methods assume that all effect estimates are based on the same set of $N$ individuals. This assumption does not always hold, for example when meta-analysing studies use different genotyping chips or different imputation panels. As a result, the covariance between effect estimates will change. In the extreme case when effect estimates are computed in two non-overlapping samples, the correlation will be zero even if there is very high LD between the two SNVs.

$\boldsymbol{C}$, as defined above, is an estimate of $\Gamma_{\mathcal{M}\mathcal{M}}$, which is the correlation matrix due to LD among the tag SNVs in the current region. We define $\boldsymbol{N}$ as a vector recording the sample size of each tag SNV, $N_{max}$ as the maximum in $\boldsymbol{N}$, and assume that every tag SNV $k$ the sample of individuals is a subset of a complete sample of $N_{max}$ individuals.

Unlike the situation where the sample size is the same for all tag SNVs, varying sample size requires to first impute the Z-statistic $z$ before computing the standardised effect size $a$. For each tag SNV $k$, we have observed a 'partial' Z-statistic, $z_k^{\circ}$, computed over the $N_k$ individuals. For a SNV $u$, our goal is to impute a 'complete' Z-statistic, $z_u$, estimated from a complete sample of $N_{max}$ people. In other words, for any SNV $u$ we wish to impute $z_u|\boldsymbol{z}_{\mathcal{M}}^{\circ}$.

To perform imputation, we require the correlation between any target complete Z-statistic, $z_u$, and any observed partial Z-statistic, $z_k^{\circ}$, (with $k \in \mathcal{M}$),

$$\boldsymbol{d}_k := \mathrm{Cor}[z_u, z_k^{\circ}] = c_{uk}\sqrt{\frac{N_k}{N_{max}}}$$

where $c_{uk} = \widehat{\Gamma}_{uk}$ is our estimate of the LD-correlation between the two SNVs. We also require the correlations among the partial Z-statistics, $\boldsymbol{z}_{\mathcal{M}}^{\circ}$. For any two SNVs $k, l \in \mathcal{M}$, the correlation of their observed partial Z-statistics can be calculated as:

$$\mathrm{Cor}[z_k^\circ, z_l^\circ] = c_{kl}\frac{N_{k\cap l}}{\sqrt{N_k N_l}}$$

where $N_k$ and $N_l$ are the number of individuals for which SNV $k$ and $l$ are available, respectively, and $N_{k\cap l}$ is the number of individuals that contributed to the calculation of the effect estimates for both SNVs $k$ and $l$. The estimation of $N_{k\cap l}$ is discussed in the next section.
We can use this to adjust the correlation matrices $\boldsymbol{C}$ and $\boldsymbol{c}$, respectively $\boldsymbol{D}$ and $\boldsymbol{d}$, the elements of which are

$$\boldsymbol{D}_{k,l} = c_{kl}\delta_{kl}\ ,$$

By defining $\delta_{kl} := \frac{N_{k\cap l}}{\sqrt{N_k N_l}}$, we can calculate the adjusted (estimated) correlation matrix $\boldsymbol{D}$, where each element is calculated as follows:

$$\boldsymbol{D}_{kl} = c_{kl}\delta_{kl}\ .$$

$\boldsymbol{D}$ and $\boldsymbol{d}$ are therefore defined as adjusted versions of $\boldsymbol{C}$ and $\boldsymbol{c}$ respectively. $\boldsymbol{C}$ and $\boldsymbol{D}$ are $q\times q$ matrices, where $q$ is the number of tag SNVs, and they are recomputed in each region. $\boldsymbol{c}$ and $\boldsymbol{d}$ are vectors of length $q$, and are recomputed for each target SNV $u$.
The conditional distribution is

$$(z_u - \mathbb{E}[z_u])|\boldsymbol{z}_{\mathcal{M}}^\circ \sim \mathcal{N}\big(\boldsymbol{d}'\boldsymbol{D}^{-1}(\boldsymbol{z}_{\mathcal{M}}^\circ - \mathbb{E}[\boldsymbol{z}_{\mathcal{M}}^\circ])\ ,\ 1 - \boldsymbol{d}'\boldsymbol{D}^{-1}\boldsymbol{d}\big).$$

Applying the simplifying assumption that $\mathbb{E}[z_u] \approx \boldsymbol{d}'\boldsymbol{D}^{-1}\mathbb{E}[\boldsymbol{z}_{\mathcal{M}}^\circ]$, similar to the assumption that took us from 1 to 2,

$$z_u|\boldsymbol{z}_{\mathcal{M}}^\circ \sim \mathcal{N}\big(\boldsymbol{d}'\boldsymbol{D}^{-1}\boldsymbol{z}_{\mathcal{M}}^\circ\ ,\ 1 - \boldsymbol{d}'\boldsymbol{D}^{-1}\boldsymbol{d}\big)$$

and therefore we impute $z_u|\boldsymbol{z}_{\mathcal{M}}^\circ$ as

$$\hat{z}_u = \mathbb{E}[z_u|\boldsymbol{z}_{\mathcal{M}}^\circ] = \boldsymbol{d}'\boldsymbol{D}^{-1}\boldsymbol{z}_{\mathcal{M}}^\circ\ . \tag{S1}$$

In order to convert $\hat{z}_u$ into the corresponding estimate of the standardised effect, we consider the (hypothetical) process of imputing each individual genotype.
If we had the individual-level genetic data, with $j$ to index individuals, each element of the $N_{max}$-element vector $\boldsymbol{g}^u$ for SNV $u$ could be imputed using genotypes from the tag SNVs $G^{\mathcal{M}}$ via $\widehat{g}_j^u = \boldsymbol{c}'_{\mathcal{M}_{(j)},u}\boldsymbol{C}^{-1}_{\mathcal{M}_{(j)},\mathcal{M}_{(j)}}G_j^{\mathcal{M}}$, where the set $\mathcal{M}_{(j)}$ can be different for each individual as each individual has a different set of tagged SNVs. The corresponding standardised effect estimate, based on linear regression, would be

$$\widehat{a}_u = \mathbb{E}[a_u|\boldsymbol{a}_{\mathcal{M}}] = \frac{(\widehat{\boldsymbol{g}}^u)'\boldsymbol{y}}{(\widehat{\boldsymbol{g}}^u)'\widehat{\boldsymbol{g}}^u}$$

The denominator of this is $(\widehat{\boldsymbol{g}}^u)'\widehat{\boldsymbol{g}}^u = N_{max}\boldsymbol{d}'\boldsymbol{D}^{-1}\boldsymbol{d}$, as opposed to $(\boldsymbol{g}^u)'\boldsymbol{g}^u = N_{max}$, and

we define the *effective sample size* as $N_{max}\boldsymbol{d}'\boldsymbol{D}^{-1}\boldsymbol{d}$. Therefore, even though we do not have the per-individual genetic data, we can impute the standardised effect $a_u$ via

$$\widehat{a}_u = \mathbb{E}[a_u|\boldsymbol{z}^{\circ}_{\mathcal{M}}] = \frac{\hat{z}_u}{\sqrt{N_{max}\boldsymbol{d}'\boldsymbol{D}^{-1}\boldsymbol{d}}} \; . \tag{S2}$$

## Estimating overlap $N_{k\cap l}$ and $\delta$

Typically, we do not know the details of the exact sample overlap for every pair of SNVs, $n_{k\cap l}$, and instead simply know $N_{max}$ and the vector $\boldsymbol{N}$. Therefore, we must derive the sample overlap based on assumptions about the dependence structure of missingness.

If each SNV has a corresponding binary missingness vector, the correlation between these missingness vectors will be maximised when the sample overlap is at its maximum, $N_{k\cap l} = \min(N_k, N_l)$. To enable the *dependent* approach, we construct a $\boldsymbol{D}$ matrix by replacing $N_{k\cap l}$ with $\min(N_k, N_l)$,

$$\boldsymbol{D}^{(dep)}_{kl} = \boldsymbol{C}_{kl}\hat{\delta}^{(dep)}_{kl} = \boldsymbol{C}_{kl}\min\left(\frac{\sqrt{N_k}}{\sqrt{N_l}}, \frac{\sqrt{N_l}}{\sqrt{N_k}}\right) \; . \tag{S3}$$

and plug $\boldsymbol{D}^{(dep)}$ into Eqs. (S1) and (S2).

If the missingness vectors are *independent* of each other, the expected overlap can be estimated as

$$\boldsymbol{D}^{(ind)}_{kl} = \boldsymbol{C}_{kl}\hat{\delta}^{(ind)}_{kl} = \boldsymbol{C}_{kl}\frac{\sqrt{N_k N_l}}{N_{max}} \; . \tag{S4}$$
